# All-Glass 100 mm Diameter Visible Metalens for Imaging the Cosmos

**DOI:** 10.1021/acsnano.3c09462

**Published:** 2024-01-17

**Authors:** Joon-Suh Park, Soon Wei Daniel Lim, Arman Amirzhan, Hyukmo Kang, Karlene Karrfalt, Daewook Kim, Joel Leger, Augustine Urbas, Marcus Ossiander, Zhaoyi Li, Federico Capasso

**Affiliations:** † John A. Paulson School of Engineering and Applied Sciences, Harvard University, Cambridge, Massachusetts 02138, United States; ‡ Wyant College of Optical Sciences, The University of Arizona, Tucson, Arizona 85721, United States; § Air Force Research Laboratory, Wright-Patterson Air Force Base, Dayton, Ohio 45433, United States; ∥ Institute of Experimental Physics, Graz University of Technology, 8010 Graz, Austria

**Keywords:** large-area, monolithic, visible metalens, astrophotography, DUV lithography

## Abstract

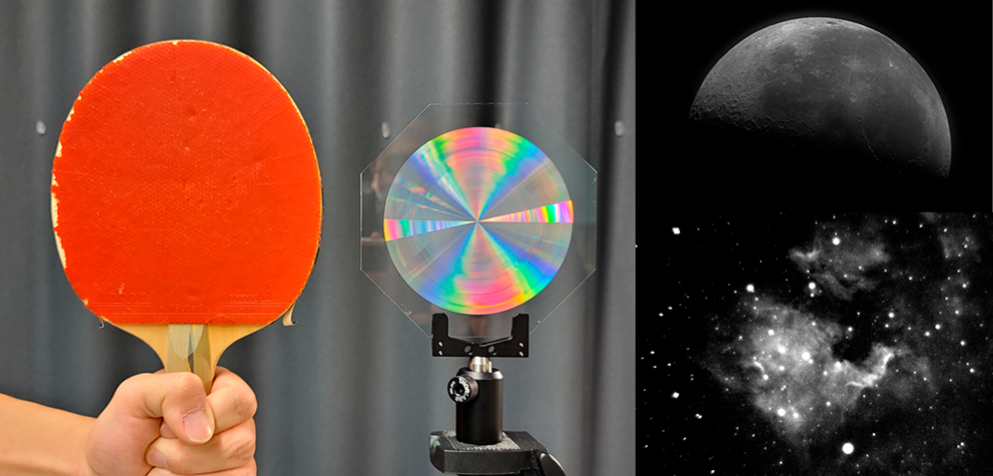

Metasurfaces, optics made from subwavelength-scale nanostructures, have been limited to millimeter-sizes by the scaling challenge of producing vast numbers of precisely engineered elements over a large area. In this study, we demonstrate an all-glass 100 mm diameter metasurface lens (metalens) comprising 18.7 billion nanostructures that operates in the visible spectrum with a fast *f*-number (*f*/1.5, NA = 0.32) using deep-ultraviolet (DUV) projection lithography. Our work overcomes the exposure area constraints of lithography tools and demonstrates that large metasurfaces are commercially feasible. Additionally, we investigate the impact of various fabrication errors on the imaging quality of the metalens, several of which are specific to such large area metasurfaces. We demonstrate direct astronomical imaging of the Sun, the Moon, and emission nebulae at visible wavelengths and validate the robustness of such metasurfaces under extreme environmental thermal swings for space applications.

Large objective apertures are necessary for optical systems that collect weak or rapidly evolving signals, such as in astronomical imaging and remote airborne surveillance, as well as in high-power laser applications to decrease the incident power density. Due to the optomechanical mounting limitations and the volumetric-and-weight scaling of refractive objective lenses with the diameter, large aperture optics must often be reflective instead of transmissive. Dielectric metasurfaces, which consist of subwavelength-spaced nanostructures with micron-scale thickness, have the potential to serve as ultralight and thin transmissive optics if they can be fabricated at scale over large areas. Although many early demonstrations of metasurfaces and binary subwavelength diffractive optics utilized electron-beam (e-beam) lithography^1−4^ or other serial point-by-point fabrication techniques, such as two-photon optical lithography,^5−7^ which led to skepticism about their practical scalability, metasurfaces are now produced at larger scales using modern semiconductor foundry photolithography^8−10^ and nanoimprinting techniques,^11−15^ alleviating such scaling concerns.

Increasing the metasurface diameter to accommodate large-aperture applications, however, does have physical and economic manufacturing limitations. E-beam lithography, which involves nanostructure-by-nanostructure writing, is not a practical method for producing metasurface lenses (metalenses) larger than several centimeters in diameter. Even with state-of-the-art tools equipped with ultrafast 100 MHz-scale e-beam oscillators and multibeam settings,^16^ the fabrication process takes several hours per sample, rendering it inefficient for large-scale production. Recent developments in e-beam writing techniques using variable-shaped beam (VSB) and cell-projection (CP) allow metasurface structures to be written over a 280 mm diameter circle area in 36 hours.^17^ Nanoimprint lithography (NIL) is a viable technique for mass-producing metasurface optics or continuous metasurface films using metasurface patterns fabricated using e-beam lithography as molds.^11,12,18^ A list of recent notable developments in scaling the diameter of metalenses is provided in Table S1.

The use of modern semiconductor foundry technology, which has supported the exponential growth in transistor density in integrated circuits since 1965,^19^ is one of the most promising approaches for mass-producing metalenses. Previous works demonstrated that *i*-line and deep-ultraviolet (DUV) projection lithography techniques, which enable high throughput nanofabrication by optically projecting the desired patterns onto a photoresist film, can be used to create centimeter-scale metalenses.^8,9,11,12,20^ However, such lithography tools have an exposure size limit of around 20–30 mm, precluding single-shot fabrication of larger area metasurfaces (Figure S1a).

Here, we experimentally demonstrate and characterize a submeter scale large diameter metalens operating at visible wavelengths, fabricated using fully CMOS compatible processes and materials. The metalens is manufactured by using DUV projection lithography and has an area that exceeds the single shot exposure size limit of the lithography tool. We do this by stitching multiple exposure fields using different photolithography reticles in an exposure cycle. The metalens is polarization-insensitive, has a 100 mm diameter and 150 mm focal length for 632.8 nm wavelength light, corresponding to a numerical aperture (NA) of 0.32 or an *f*-number of 1.5. We choose fused silica (SiO_2_) as the sole constituent material for this monolithic metalens not only for its low absorption in the visible and CMOS process compatibility but also for its high laser-induced damage threshold, which is suitable for high-energy applications. To better meet the design and the choice of material, we have developed a vertical fused silica etching process, which we have studied and refined over the years since our initial efforts in 2019.^8^ Fused silica is better tolerated in foundry conditions; although there exist other optical glass materials with higher refractive indices and thus less stringent design constraints, many of them contain high vapor pressure materials or plasma-chamber incompatible heavy metals (e.g., Zn, Ca, K, Na, Mg, Ba, Pb) which could contaminate processing tools and other wafers during post processing. Also, the thermal robustness enables various coatings (e.g., antireflection or antifouling coats) to be deposited directly onto the metalens or its backside, which is an essential requirement for such metalenses to be utilized in versatile astronomical applications. We show that this photolithography-based manufacturing approach for metalenses larger than the exposure size limit, which was previously limited to the infrared spectrum^21−23^ and to silicon wafers, can be extended to the visible spectrum^8,12^ and to more robust materials. The approach introduced in this study can be generalized to other materials with different refractive indices, although the process compatibility and the dry etch chemistry will need to be adapted accordingly. This is also a major manufacturing leap forward from our earlier work^8^ in two notable aspects: first, by increasing the size scale tenfold, which translates to a hundredfold increase in terms of the total number of nanopillars (18.7 billion); and second, by developing a much improved vertical glass etching process, resulting in a greatly reduced sidewall tapering and therefore reducing the discrepancy between the design geometry and the fabricated results. In addition, we show the feasibility of mass-production by fabricating several 100 mm diameter metalenses. The metalens’ imaging quality is evaluated through its point-spread-function (PSF), modulation transfer function (MTF), focusing efficiency, and transmitted wavefront error via full-aperture interferometry. Then, we further simulate various fabrication error scenarios and quantify their individual impacts on the wavefront and imaging quality. These simulations demonstrate that large area metasurfaces fabricated with multiple exposures and field stitching face fundamentally different aberration and efficiency challenges as compared to single-shot photolithography metalenses and other ground refractive elements. We also show the survivability of the meta-optical device in extreme environments by evaluating its optical performance after thermal shock and temperature cycling across a 400 °C range. Finally, we illustrate the remote and high-throughput imaging capabilities of the large diameter metalens by photographing celestial objects (the Sun, the Moon, and an emission nebula) in the visible,^24,25^ using the 100 mm diameter metalens as the only imaging lens.

In summary, the key contributions of this study are the realization of a significant number of nanostructures (18.7 billion) in a visible-spectrum metasurface optic, elimination of the sidewall taper with an improved vertical glass etch process, systematic simulation study of fabrication error tolerance with respect to optical aberrations, an experimental study of optical performance after thermal shocks, and remote imaging of both the bright and dim celestial objects with the metasurface optic.

## Results and Discussion

### Design and Fabrication of Metalens

Metasurfaces comprise nanostructures that impart a designed local wavefront or polarization transformation to incident light and can demonstrate unconventional functionality and performance with a very thin form factor that is difficult to achieve with conventional bulk optics. For instance, full Stokes polarimetry imaging has been realized with a single metasurface coupled to a commercial camera sensor,^26−28^ and an ultrathin perforated membrane metalens has been used to focus extreme ultraviolet radiation in transmission.^29^ With precision design and manufacturing, metalenses have achieved diffraction-limited, high-efficiency performance,^30,31^ and have attained broadband aberration correction when used in conjunction with existing refractive optics.^32,33^

To design a 100 mm diameter metalens, we first divide the 100 mm diameter region into 25 square sections (5 × 5 square array). Each section occupies a 20 × 20 mm square area (Figure S1b), which is smaller than the exposure size limit of the DUV lithography tool used in this study (22 × 22 mm). We intentionally chose an odd number of arrays to ensure that the center of the metalens coincides with the center of a discretized section. This prevents unwanted scattering of light along the optic axis that may occur due to the stitching of the discretized fields near the metalens center, which may degrade the imaging quality. Since the metalens is rotationally symmetric, the full 25 sections can be represented with just seven distinct sections, six of which are repeated at four rotation angles (0°, 90°, 180°, and 270°) and one which is located at the metalens’ center.

The metalens is composed of fused silica nanopillars with diameters ranging from 250 to 600 nm, which have a constant edge-to-edge spacing of 250 nm and a height of 1.5 μm. This keeps both the pattern and the gap dimensions above the 200 nm single-exposure feature size limit of the DUV lithography tool used (ASML PAS 5500/300C DUV Wafer Stepper, λ = 248 nm). Choosing the edge-to-edge spacing to match the photolithography wavelength is a straightforward heuristic for metalens design that does not require the use of specialized proximity effect correction techniques; the manufacturable nanostructure critical dimension can be improved further with multiple exposure processes or reticle optimization, which is beyond the scope of this study. Performing DUV lithography with a shorter illumination wavelength or using immersion DUV can also further reduce this edge-to-edge spacing design constraint for a single shot exposure process down to 40 nm. The simulated transmitted phases and amplitudes versus the nanopillar diameter are plotted in Figure S2a, which demonstrates phase coverage between π/2 and 3π/2 radians for the pillars alone, and 0 to 3π/2 when the empty areas are included as a part of the design. Although full 0 to 2π radian phase coverage can be achieved if the nanopillar height is increased to 2.1 μm and the minimum diameter is decreased to 100 nm, this increased width-to-height aspect ratio significantly decreases the structural integrity and hence manufacturability. We have thus limited the nanopillars to a maximum aspect ratio of 1:6. We designated the empty area (i.e., absence of a nanopillar) as a zero-phase element and included such empty areas in the design of the metalens to compensate for the limited library phase coverage. A library with reduced phase coverage can still be used to produce a focusing metalens at the expense of reduced focusing efficiency and increased background scattered light.^8^ The focusing efficiency is reduced since there are empty areas of the lens without nanostructures where the light can pass straight through and not be deflected to the focus. Unwanted diffraction orders are produced at the transverse interface between the filled and empty radial region. This effect is more pronounced at the edges of the lens with higher spatial frequencies, which can reduce the effective NA of focusing for extremely fast lenses. While both effects increase the background zeroth order transmission of the metalens, they generally do not degrade the focusing quality of large-area lenses with finely sampled high spatial frequency zones, as measured by the modulation transfer function, or equivalently the focal spot quality. The focal spot is much brighter than the transmitted background beyond typically measurable sensor dynamic ranges, and the lens edges still deflect light to constructively interfere at the focus.

The diameter profile along the metalens radial direction is determined by selecting the nanopillar element at each radial position *r* with a transmitted phase φ(*r*) that most closely matches the desired ideal hyperbolic focusing profile:^34,35^
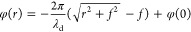
1where λ_d_ is the design wavelength, *f* is the focal length of the metalens, and φ(0) is the phase at the center of the metalens. When eq 1 is satisfied, the transmitted light from each point on the metalens interferes constructively at the focal point; this is equivalent to having a spherical wavefront that converges at the focal point. We set φ(0) to that of the transmitted phase of the nanopillar with the largest diameter in the library (600 nm) so that the phase wrapping zone transition between the largest and smallest nanostructures is located the furthest from the optical axis. The nanopillars at each radial position are placed uniformly across the azimuthal direction with a minimum edge-to-edge spacing of 250 nm. The full metalens design comprises 18.7 billion nanopillars.

The seven individual stitching sections of the metalens are indexed as reticles 1 to 7 (Figure S1b), respectively. The center three reticles (reticles 1–3) were made by an industry-grade photomask manufacturer. The outer four reticles (reticles 4–7) were made in-house using a laser photomask writer with a larger critical feature size compared to that of the photomask manufacturer (Figure S1c). The detailed process of the metalens design file generation, as well as the strategies employed to reduce file size and write times, can be found in the Supporting Information.

**Figure 1:**
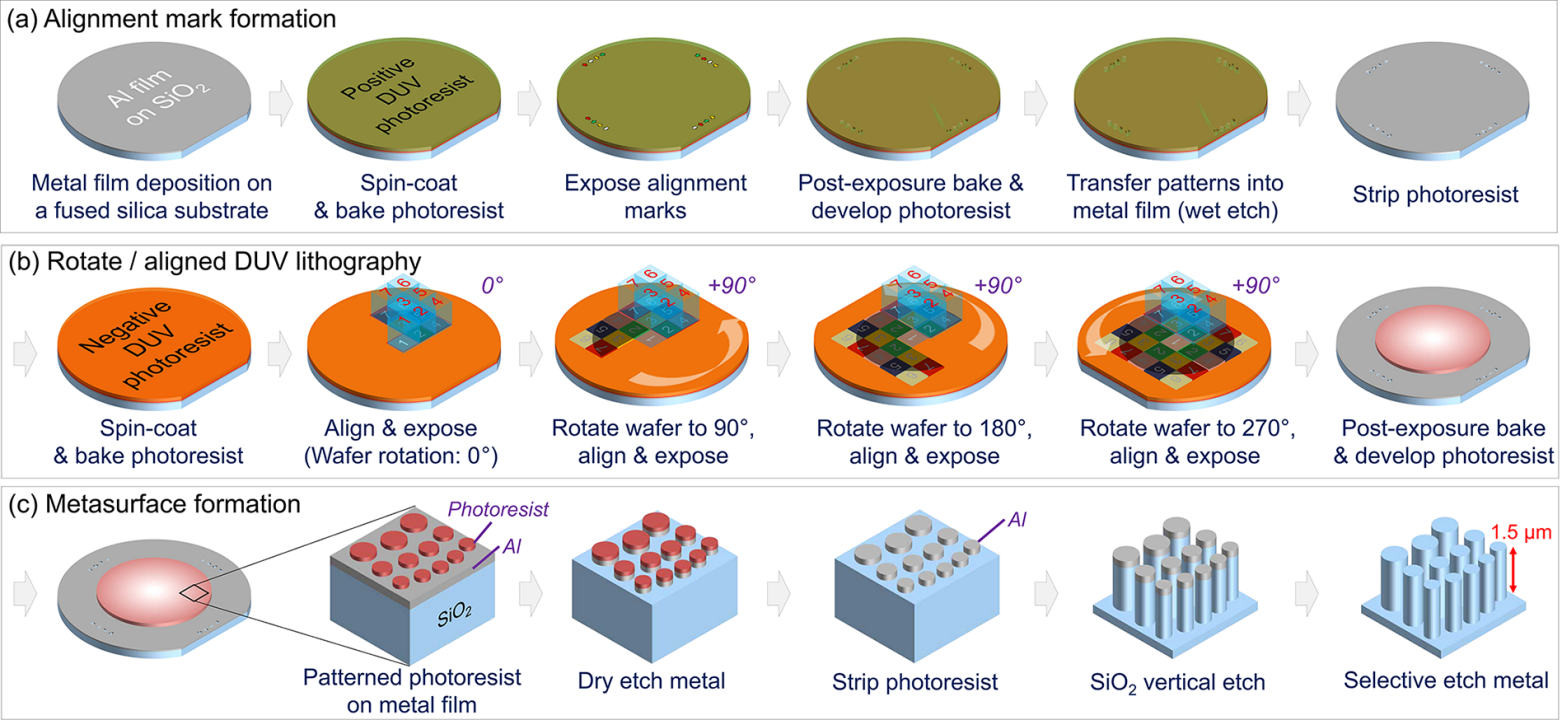
Fabrication process of the 100 mm diameter, all-glass metalens. (a) Global alignment marks are etched into aluminum film on a fused silica substrate. (b) A 100 mm diameter metalens pattern, segmented into 25 sections, is formed on the aluminum film using a negative DUV photoresist with 7 photomasks. (c) Using the photoresist pattern as an etch mask, the metalens pattern is transferred to the aluminum film with an Ar/Cl_2_ plasma etch. Using the patterned aluminum as a hard etch mask, we perform a vertical SiO_2_ etch into the fused silica substrate until the desired pillar height of 1.5 μm is reached. The residual Al film is removed using a Cl_2_ plasma etch, leaving only the fused silica nanopillars.

As the first fabrication step, we coat a 150 mm (6-in.) fused silica wafer with a 150 nm-thick aluminum (Al) film (Figure 1a), antireflective coating (ARC), and positive DUV photoresist layers (Figure S3a). We then create global alignment marks for the DUV lithography process. The alignment marks (i.e., fiducials) are positioned and oriented such that the four alignment marks remain at the same location when the wafer is rotated by 0°, 90°, 180°, and 270° (Figures S3b,c).

We then coat the wafer with another ARC layer and a negative DUV photoresist layer, which serves as the etch mask for metalens pattern creation into Al. We opt to use a negative photoresist due to its advantageous process window compared to positive photoresist when creating isolated structures: overexposure of positive resist can cause shrinkage or delamination of the resist patterns, while patterns on negative resist simply become enlarged. As shown in Figure 1b, the wafer is first loaded into the DUV lithography tool at 0° orientation, and reticles 1–7 are aligned and exposed. The alignment error of the used stepper lithography tool is less than 45 nm, which is about 7% of the target wavelength and thus not expected to significantly distort the transmitted wavefront. State-of-the-art photolithography tools can achieve overlay errors of down to 1 nm. The wafer is then rotated to 90°, and reticles 2–7 are exposed. The same process is repeated for the 180° and 270° wafer orientations (Figure 1b). The alignment and exposure of the entire metalens is fully automated and takes less than 20 min per wafer.

After the metalens pattern is formed in the photoresist, it is transferred to the ARC layer by using an Ar/O_2_ reactive ion etch (RIE) (Figure S4b) and into the Al layer using Ar/Cl_2_ inductively coupled plasma reactive ion etching (ICP-RIE), as shown in Figure 1c. SiO_2_ is thermodynamically protected against chemical etching by Cl_2_ plasma^8^ and acts as an etch stop layer. Both the ARC and resist layers are then stripped using a downstream oxygen plasma ashing process (Figure S4c). Using the patterned Al as a hard etch mask, we then vertically etch into the fused silica substrate with ion-enhanced inhibitor etching using our optimized octafluoropropane (C_3_F_8_) ICP-RIE process (Figure S5a) until the etch depth reaches 1.5 μm. The measured uniformity of the etching speed across the 100 mm diameter region is provided in Figure S6, which shows that the maximum resulting etch depth difference across the entire 100 mm diameter metalens is approximately 80 nm, corresponding to 5% of the target pillar height. This height deviation is associated with a phase error smaller than the quarter-wave deviation acceptable under Maréchal criterion (Figure S2d). Once the desired etch depth is reached, the residual Al film on top of the nanopillars is selectively etched away using an Ar/Cl_2_ ICP-RIE process, leaving the fused silica nanopillars. More details on the fabrication process can be found in the Materials and Methods section and the Supporting Information.

### Optical Performance Characterization

**Figure 2:**
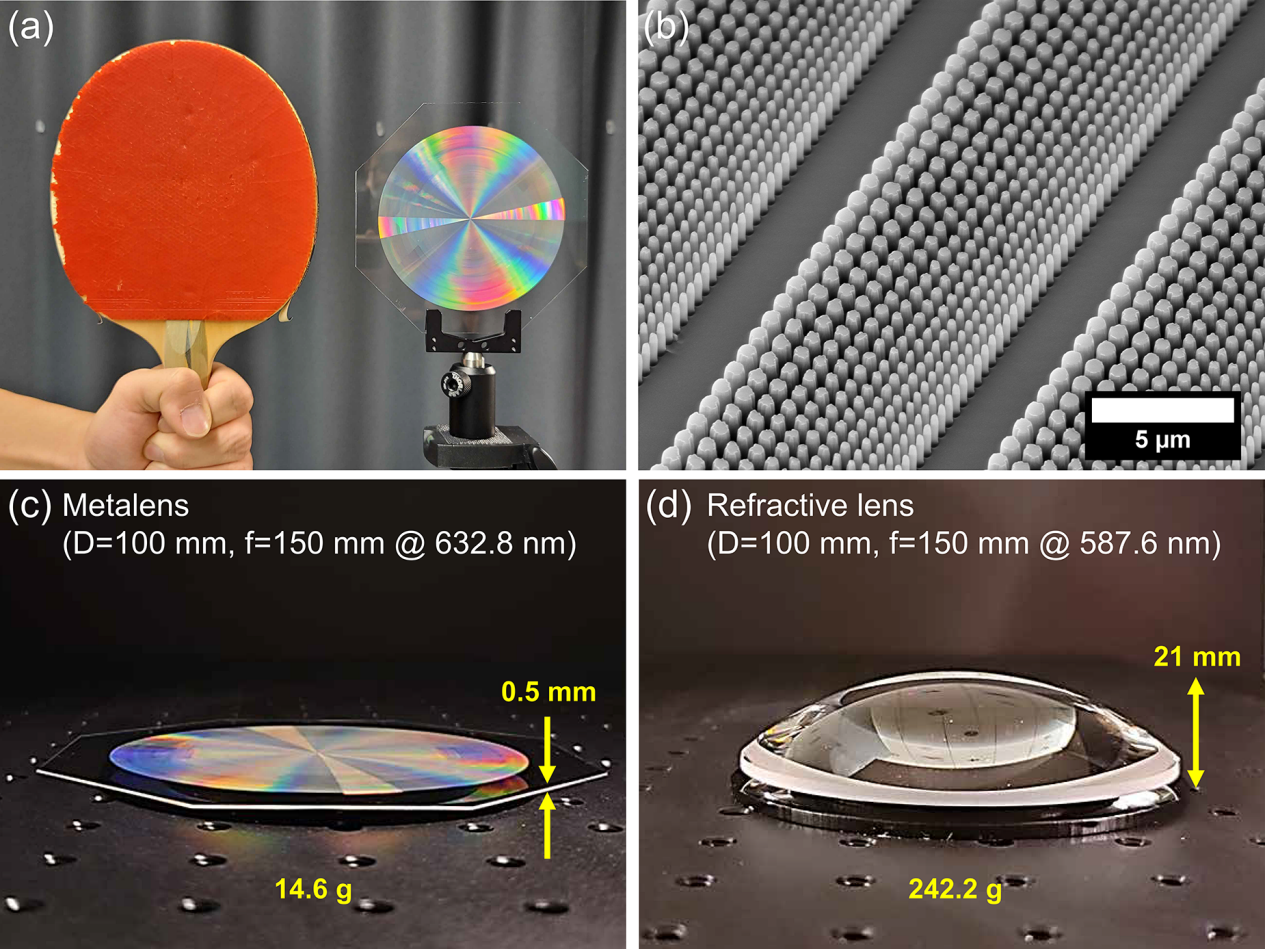
Photographs and SEM images of the 100 mm diameter, all-glass metalens. (a) Photograph of the metalens compared with a table tennis racket. (b) SEM image of the fused silica nanopillars comprising the metalens. More SEM images are provided in Supporting Information (Figure S7). Photographs taken from the side of (c) the metalens (*f* = 150 mm at λ = 632.8 nm) and (d) a plano-convex lens made of N-BK7 glass with a similar diameter (100 mm) and focal length (*f* = 150 mm at λ = 587.6 nm, Edmund Optics #19-904).

A photograph and a tilted scanning electron microscope (SEM) image of the 100 mm diameter metalens are shown in Figure 2a,b, respectively. The vertical and smooth sidewalls of the etched fused silica nanopillars are visible, which is a notable improvement from previous fused silica metalens work that resulted in tapered and rough sidewalls.^8^ More detailed SEM images of the metalens are provided in Supplementary Figure S7. Figure 2c,d present a side-by-side comparison between the fabricated metalens and an off-the-shelf plano-convex refractive lens made of N-BK7 glass with MgF_2_ antireflection coating (Edmund Optics, #19-904) with a similar diameter (*D* = 100 mm) and focal length (*f* = 150 mm at λ = 587.6 nm). The metalens including the substrate is 42 times thinner (0.5 mm vs 21 mm) and 16.5 times lighter (14.6 g vs 242.2 g) than the refractive lens counterpart. This scalable technology enables mass-production of all-glass 100 mm diameter metalenses, as presented in Figure S27.

**Figure 3:**
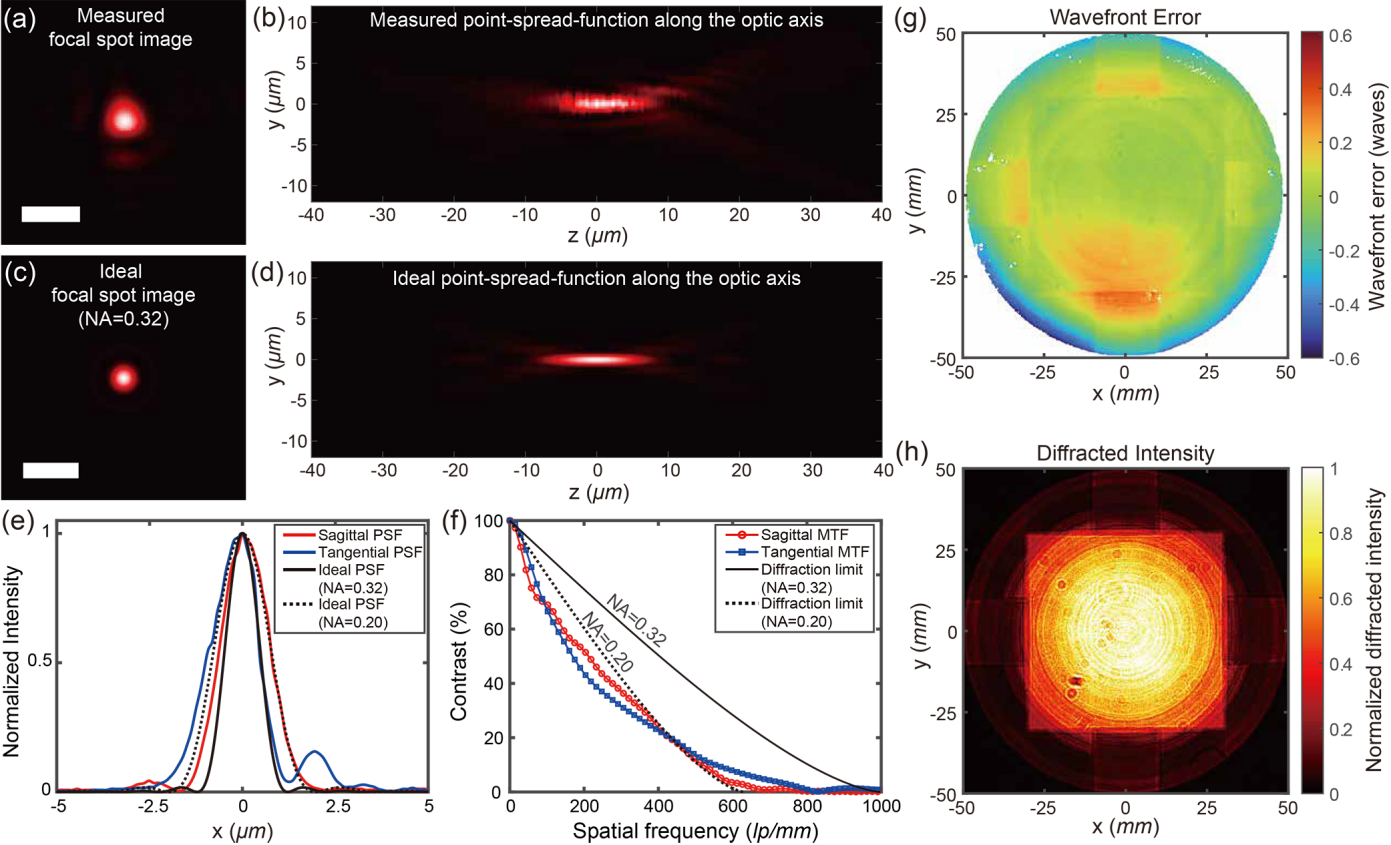
Optical characterization of the 100 mm diameter, all-glass metalens. (a) Focal spot image of the fabricated metalens at the design wavelength (λ = 632.8 nm). Scale bar: 3 μm. (b) Point-Spread-Function (PSF) of the metalens along the optic axis. *z* = 0 μm represents the focal plane (*f* = 150 mm). (c) Simulated focal spot image of an ideal lens with NA = 0.32 (scale bar: 3 μm), and (d) simulated PSF of an ideal lens along the optic axis. (e) Focusing profiles along the sagittal (vertical) and the tangential (horizontal) plane of the metalens’ focus shown in (a) and simulated focusing profiles of diffraction-limited lenses with NAs of 0.32 (100 mm aperture, 150 mm focal length) and 0.20 (60 mm aperture, 150 mm focal length), respectively. (f) Modulation transfer function (MTF) of the metalens, and diffraction-limited MTFs of lenses with NA of 0.32 and 0.20, respectively. (g) Measured wavefront error map of 100 mm diameter metalens, obtained from full-aperture interferometry. The interferometry alignment terms (i.e., piston, tip, tilt, and power) are removed. (h) Intensity image of the 1st-order diffracted beam from the metalens, which corresponds to the intensity contribution from each point of metalens to the focal point. The diffracted intensity is higher in the central 6 × 6 cm region, at which industrial-grade photomasks were used (reticles 1–3), compared to the outer region at which lab-written photomasks were used (reticles 4–7). The mismatch in diffraction efficiency between the inner and outer regions causes a broadening of the observed focusing behavior from that of an ideal 100 mm diameter lens.

To evaluate the focusing quality of the metalens, we illuminate the flat backside of the metalens with the expanded and collimated beam of a Helium–Neon laser (λ = 632.8 nm). We then acquire the focal profile along the optical axis separately using a horizontal microscope (Figure S8) and a point-source microscope attached to a coordinate measuring machine (Figure S9). The measured distance to the focal plane from the metalens is 149.97 ± 0.18 mm, which agrees well with the designed focal length of 150 mm (Figure S9). Figure 3a,b show a transverse and a longitudinal cut of the measured point-spread function (PSF). For comparison, the simulated transverse and longitudinal focusing profiles for a diffraction-limited metalens with NA = 0.32 are provided in Figure 3c,d.

The Strehl ratio of the fabricated metalens is 0.6. Furthermore, the diameter of the first ring of minimum intensity around the focal point of the metalens is 3.4 μm, whereas the ideal Airy disk diameter for a diffraction-limited lens with NA = 0.32 would be 2.3 μm, indicating the presence of aberration in the metalens. The measured PSF, degraded by the wavefront aberration, resembles that of a lens with an effective NA of 0.2, which corresponds to a lens with a diameter of 60 mm and a focal length of 150 mm that has an Airy disk diameter of 3.86 μm (Figure 3e). This similarity becomes more apparent when the 2D modulation transfer functions (MTFs) are calculated from the PSF images and compared with the ideal MTFs for lens NAs of 0.32 and 0.2, as shown in Figure 3f.

The primary cause of the aberration is the reticle quality difference between the industry-grade center sections (reticles 1–3) and the lab-made outer sections (reticles 4–7). In the inner region, all nanopillars are present as per the design, but in the outer regions, the small diameter pillars are missing due to those features not being resolved in the reticles fabricated with the lower resolution in-house laser writer. Both errors can be avoided by using reticles from a single manufacturing source.

The effect of the missing pillars on the PSF becomes more apparent when the transmitted phase and the diffracted amplitude information are decoupled by using interferometry and diffraction intensity imaging. The deviation of the transmitted wavefront phase of the metalens from the ideal, i.e., wavefront aberration function (WAF), is obtained using full-aperture interferometry. For this, a commercial Fizeau interferometer (Zygo VeriFire ATZ, Zygo Corporation) is used in a double-pass nulling interferometry configuration, in which the metalens-focused beam is reflected back by a return sphere (i.e., high-accuracy spherical ball), and produces an interferogram at the interferometer aperture (Figure S10). More details of the interferometric procedure are provided in the Supporting Information. The map of the metalens WAF is shown in Figure 3g, with the root-mean-square (RMS) error over the entire 100 mm aperture of 0.08 λ and a peak-to-valley (PV) error of 0.64 λ (after interferometry alignment terms, i.e., piston, tip/tilt, and power). The inner 60 × 60 mm section, fabricated with industry-grade photomasks, displays lower RMS and PV errors of 0.05 λ and 0.28 λ, respectively. These values indicate that the industry-grade fabrication technique can attain phase error values close to the diffraction-limit criterion of an RMS error below 0.075 λ and a PV error below 0.25 λ.^35−37^ The fitted Zernike polynomial coefficient values to the WAF are provided in Figure S11 and Table S2, which indicate that the optical aberrations are dominated by second- and fourth-degree contributions. The expected tilt dependence of the as-manufactured metalens’ wavefront (i.e., coma) was also successfully evaluated and confirmed interferometrically (Figure S15). The RMS wavefront error rises to 1 λ for tilts around 10 arcseconds.

The diffracted intensity after the metalens (Figure 3h), i.e., light contributing to the focus, shows the stark difference between the efficiency of the inner (reticles 1–3) and outer (reticles 4–7) lens sections. The diffracted image is captured at a traverse plane 11 mm in front of the focal plane, in the direction toward the metalens. We find that 86% of the power that contributes to the focus comes from the inner region, which comprises only 46% of the total metalens area, causing the focal spot to resemble that of a square 60 × 60 mm lens instead. As discussed earlier, the reduced diffraction intensity in the outer region is mainly due to missing small diameter pillars in the in-house fabricated photomasks. The measured focusing efficiency of the entire metalens, relative to that of the equivalent off-the-shelf MgF_2_-coated plano-convex refractive lens in Figure 2d, is 40.4%. However, when only the inner region is profiled, the focusing efficiency rises to 63.1%, which agrees with the diffracted intensity profile. Thus, the reticle quality difference across each exposure field is the primary cause of the aberrated PSF and suggests that the metalens performance will be optimized when all of the reticles are industry-grade.

### Fabrication Error Tolerance Study

Exposure field-linked fabrication errors are a specific problem for metasurface optical elements made with CMOS-compatible manufacturing processes since such rectilinear imperfections do not exist for traditional optics manufacturing methods such as diamond turning or other mechanical grinding and polishing techniques. To fully characterize such fabrication errors on the imaging quality degradation and their corresponding tolerances, we conduct a simulation-based study quantifying the following effects: (1) unresolved nanopillars below certain diameters, (2) a uniform shift in nanopillar diameters, and (3) a uniform shift in nanopillar height. Effects (1) and (2) can occur during photomask manufacturing or through shifts in exposure and development conditions, and (3) can occur if the nanopillars are either under- or overetched. We consider the effects of these errors when they are applied to each reticle-linked section individually and across the inner and outer sections (Figure S12).

Since the full-lens simulation of 18.7 billion nanostructures is impractical on full-wave Maxwell equation solvers, we employ the locally periodic assumption^38^ and take the transmitted field to be equal to the stitched fields from individual nanopillars. We numerically discretize the metalens into 100,000 annular rings, each divided into 100 sections evenly in the azimuthal direction, resulting in a total of 10 million arc sections. This discretization choice allows for fine resolution of the rapidly varying radial transmission behavior without expending additional elements to resolve the slowly varying azimuthal behavior. We then propagate the transmitted electromagnetic field from each arc section toward the focal plane using a vectorial propagator,^39^ weighting each arc section with its area. The focal plane complex fields are used to calculate the MTF, Strehl ratio (volume under the 2D MTF relative to that of the diffraction limit), and focusing efficiency (fraction of incident power that passes through 3 Airy disk diameters centered on the optic axis). Further details of the simulation are provided in the Supporting Information.

**Figure 4:**
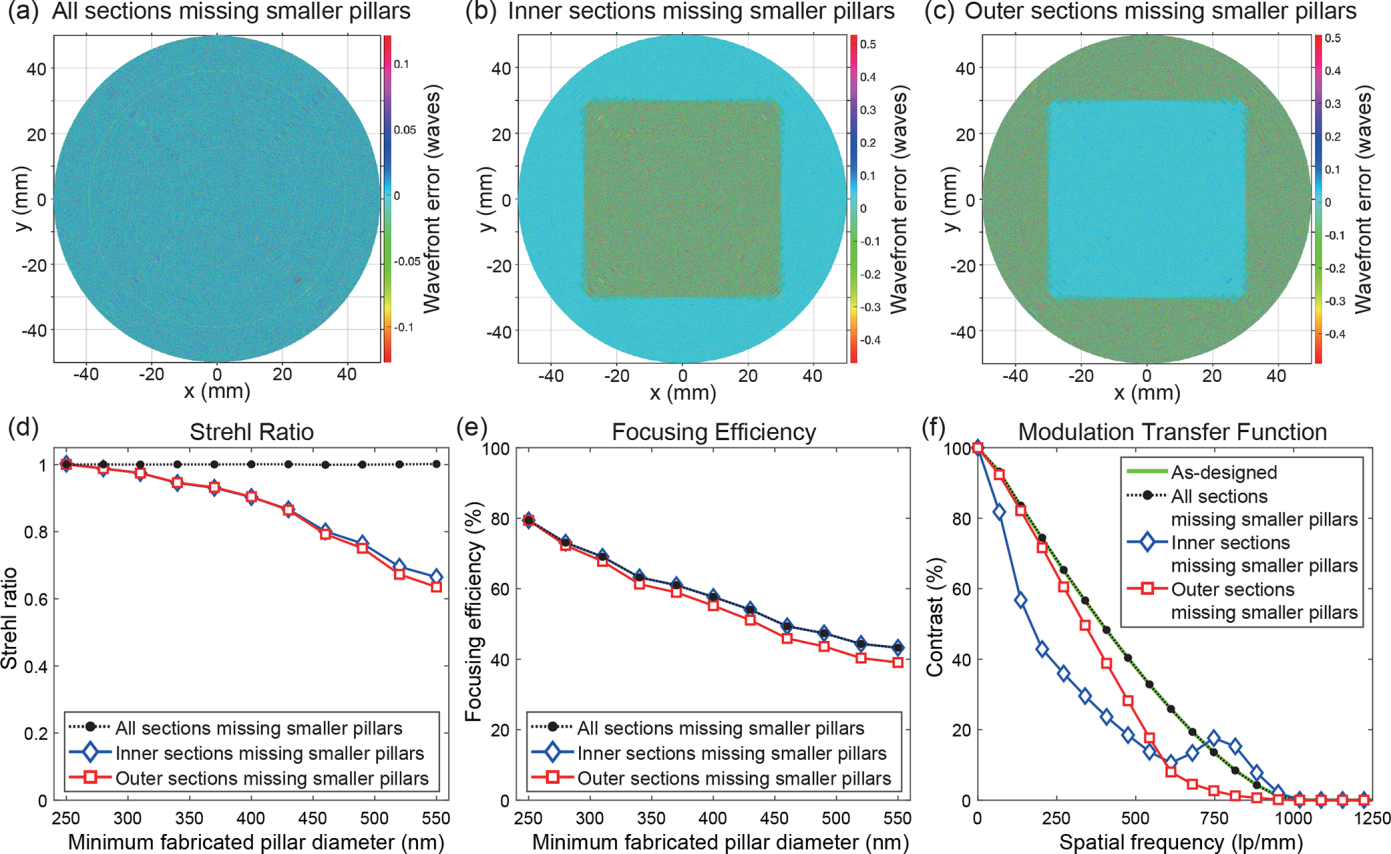
Simulation-based study on the effects of missing smaller pillars in different regions of metalens on its imaging quality and efficiency. Simulated wavefront error of a metalens missing small pillars (a) in all sections (reticles 1–7), (b) in the inner sections only (reticles 1–3), and (c) in the outer sections only (reticles 4–7), respectively. The surface plots show the resulting wavefront error when the smallest fabricated nanopillar diameter is 550 nm. (d) Simulated Strehl ratio versus the minimum fabricated nanopillar diameter for all sections, inner sections only, and outer sections only, respectively. The Strehl ratio remains diffraction-limited when the nanopillars are missing uniformly across the entire metalens, while a mismatch in the loss of nanopillars result in poorer imaging quality due to the resulting wavefront error. (e) Simulated focusing efficiencies when all sections, inner sections only, and outer sections only are missing smaller pillars. All cases exhibit degradation of overall focusing efficiencies due missing smaller diffracting elements. (f) Normalized modulation transfer function (MTF) for each scenario of missing pillars when the smallest fabricated nanopillar diameter is 550 nm. The MTF contrast curves are normalized to their respective zero-frequency values.

Figure 4a–c present the simulated wavefront of a metalens where nanopillars smaller than a given threshold diameter are unresolved for (a) all 7 sections, (b) only the inner sections (reticles 1–3), and (c) only the outer sections (reticles 4–7), respectively. The simulated Strehl ratios and focusing efficiencies of each case with respect to the minimum fabricated pillar diameters are shown in Figure 4d,e, respectively. The Strehl ratio degrades when either the inner or outer sections’ smaller pillars are missing but remains near unity for the case when the pillars below a given diameter are equally missing over all sections. This reduction in the Strehl ratio occurs due to incomplete constructive interference near the focus due to a mismatch in diffracted intensity contributions from different parts of the lens, which broadens the transverse peak. However, when all sections have similar amounts of missing pillars, such diffracted intensity variations are eliminated and one obtains diffraction-limited focusing. In comparison, the focusing efficiency in all three cases decreases monotonically as the minimum fabricated pillar diameter increases since more light passes straight through the metalens and is not deflected to the focus. This is consistent with previous findings, in which metalenses with unfabricated smaller structures were still able to produce diffraction-limited focusing albeit at reduced efficiencies.^8^ The MTFs for the three scenarios when the minimum fabricated nanopillar diameter is 550 nm are shown in Figure 4f. The MTF curves are normalized to unity at their zero-frequency values, respectively. When all sections have missing smaller diameter pillars, the MTF remains diffraction-limited. When the inner sections have missing pillars, the MTF at lower spatial frequencies is reduced and vice versa for outer sections having unfabricated pillars. More detailed plots of the MTF behavior under other missing pillar conditions are provided in Figure S13a–c.

For fabrication errors other than section-linked missing nanopillars, the Strehl ratio remains diffraction-limited, although the focusing efficiency decreases. The calculated Strehl ratios and focusing efficiencies resulting from nanopillar diameter shifts are provided in Figure S13d,e, respectively. The metalens retains a diffraction-limited performance for nanopillar diameter shifts less than 100 nm. Similarly, an error in nanopillar height, which can occur during the final SiO_2_ etching process, does not significantly affect the Strehl ratio (Figure S13f) but reduces the focusing efficiency. This retention of high Strehl ratio focusing arises because the phase relationship between nanopillars of different diameters remains largely unchanged with respect to systematic diameter and height variations (Figure S2a,d). However, as the relative phase between the empty areas and the nanopillars shifts, the efficiency is reduced due to out-of-phase interference at the focal plane. The Strehl ratio and focusing efficiencies remained nearly unaffected by individual reticle misalignment errors, also known as “stitching errors”, up to 100 nm in both lateral and vertical directions. These errors caused only a negligible shift in the Strehl ratio, approximately on the order of 10^–6^ from unity for each nanometer of reticle overlay error.

Beyond fabrication errors, we investigate the role of imperfections in the measurement apparatus, which arise easily due to the challenges of aligning and characterizing large aperture optical elements. If the illuminating light is nonuniform, for example, with a Gaussian intensity profile (Figure S14a), the measured PSF becomes aberrated and the Strehl ratio falls below unity, even for a perfectly fabricated metalens (Figure S14b). However, the Gaussian width of the illumination must be substantially smaller than the lens diameter (20 mm Gaussian width) to reduce the Strehl ratio below the diffraction-limited level of 0.8. Tilts in the illuminating field (i.e., off-axis illumination) can also introduce field-dependent aberrations in the PSF. Figure S16 exhibits the simulated reduction in measured Strehl ratio as a function of incident angle tilts. The illuminating field must be normally incident to within 10 arc seconds to provide an accurate measurement of the metalens focusing quality, which agrees with interferometric measurements shown in Figure S15. Our PSF characterization setup (Figure S8) has an illumination uniformity and tilt tolerance below these thresholds.

Table S3 summarizes the main conclusions of the fabrication and measurement error simulation study. Only a mismatch between the unfabricated pillars in the inner and outer sections produces the Strehl ratio reduction observed in the experimental focal spot.

### Meta-Imaging the Cosmos

**Figure 5:**
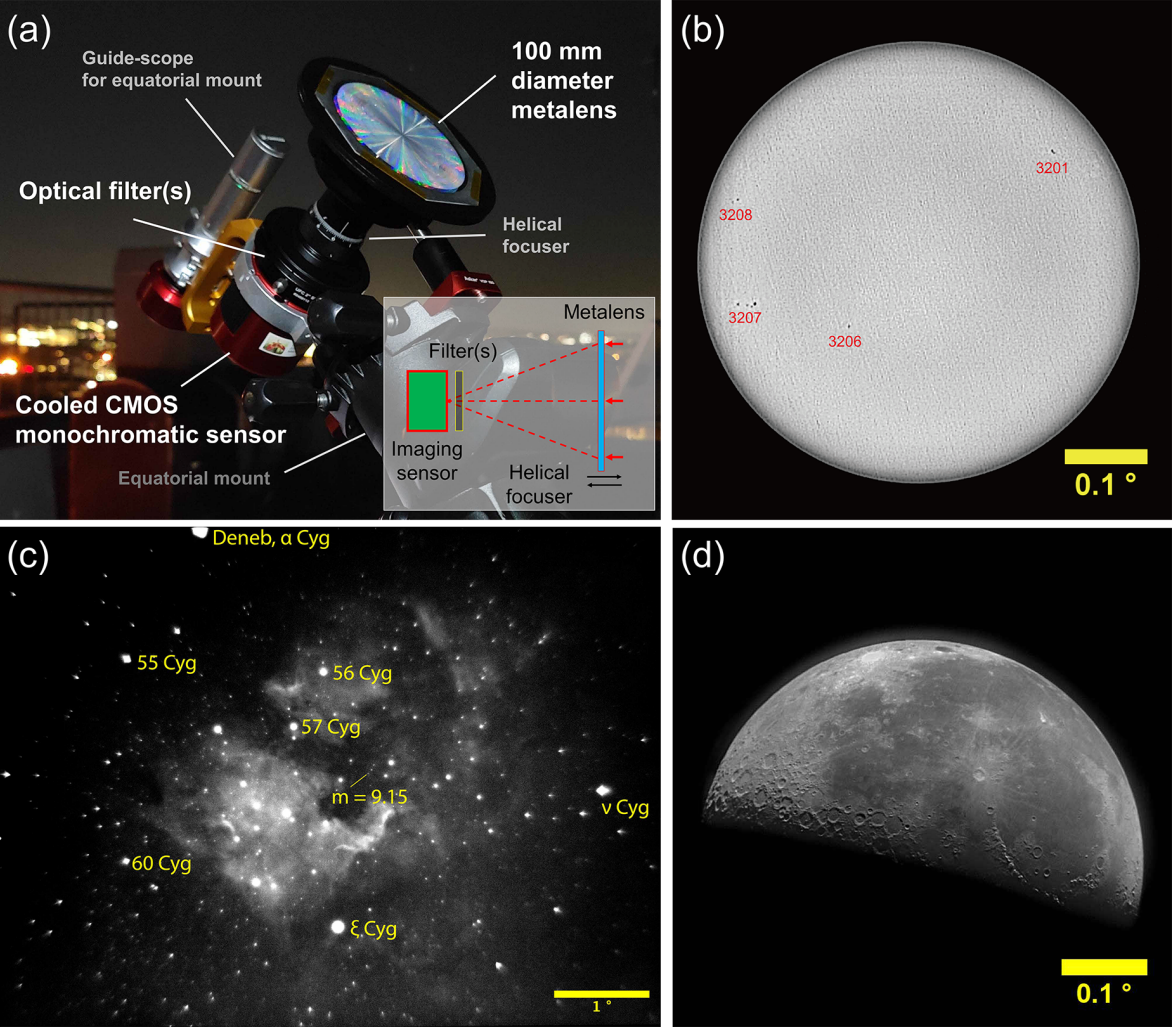
Imaging the cosmos in the visible with 100 mm diameter wide field-of-view (larger than 0.5°) meta-astrophotography apparatus. (a) Photograph of the astro-imager comprising only a 100 mm diameter metalens, an exchangeable optical filter, and a cooled CMOS monochromatic sensor, mounted on an equatorial mount guided by a guide-scope. (b) Acquired image of the Sun with a neutral density filter (OD 3.0) and a 1 nm bandwidth bandpass filter centered at 632.8 nm. Image taken on Feb. 01, 2023, Cambridge, Massachusetts, USA. Identified sunspot group numbers are labeled. (c) Acquired image of the North America Nebula (NGC 7000, in the constellation Cygnus) with a 7 nm bandwidth *H*-α filter (λ = 656.28 nm). Image taken on May 13, 2022, Cambridge, Massachusetts, USA. Notable celestial objects are labeled. The imaging system can detect stars down to an apparent magnitude of 9.15. (d) Acquired image of the Moon at its last quarter phase, taken with a 1 nm bandwidth bandpass filter centered at 632.8 nm. Image taken on Aug. 18, 2022, Cambridge, Massachusetts, USA. High-resolution images are provided in the Supporting Information, and unprocessed and processed images are archived in a public data repository.^49^

To illustrate the imaging performance of the fabricated metalens, we built a wide field-of-view (i.e., larger than 0.5°) meta-astrophotography apparatus using only the 100 mm diameter metalens (without any other optical lenses or mirrors), a narrowband color filter, and a cooled CMOS imaging sensor placed at the metalens focal plane (Figure 5a). The distance between the metalens and the imaging sensor is controlled by a helical focuser and the apparatus is enclosed to eliminate stray light. This is not a telescope, as there is no second lens and no magnification involved. The meta-astrophotography system is mounted on an equatorial mount with a guide-scope for real-time sky tracking and is used to capture images of sunspots on the Sun (with a 633 nm bandpass filter, Figure 5b), the North America Nebula (with a 656.28 nm Hydrogen alpha bandpass filter, Figure 5c), and the Moon (with a 633 nm bandpass filter, Figure 5d). Higher resolution images, unprocessed images, the details of the image acquisition methods, and more photographs of the metalenses are provided in Figures S17–S19, Figures S26–S30.

Such imaging of celestial objects in the visible wavelengths illustrates that the 100 mm diameter all-glass metalens is suitable for remote imaging applications. To deploy meta-optics in remote imaging platforms such as high-altitude UAV platforms, low-earth orbit satellites, or off-world exploration vehicles, the nanostructures must withstand extreme environmental stresses such as large temperature swings, cosmic radiation, and vibration.^40−42^ To test whether the all-glass metalens can survive harsh environments, we devised a thermal shock and heat stress cycling test resembling that of the United States Department of Defense Test Method Standard (MIL-STD-883F). The subject in the testing was cyclically moved between a cold reservoir (liquid nitrogen, −195.8 °C) and a hot reservoir (hot plate, 200 °C). The subject remained in each thermal reservoir for 10 min to reach thermal equilibrium, while the sample transfer time between the two thermal baths was less than 5 s to induce thermal shock. More details of the devised test method are provided in Figure S20. After performing the stress test on a 10 mm diameter all-glass metalens^8^ for 10 cycles and returning the sample to room temperature, we do not observe significant change in the optical performance (Figure S21) nor physical damage after 15 cycles (Figure S22). This is due to fused silica having a low-level of impurities and also a near-zero thermal expansion coefficient (α ≈ 0.5 × 10^–6^/*K*)^43^ which would result in an approximately 0.01% shift of the metalens radius over the 400 °C temperature range. As the thermo-optic coefficient (*dn*/*dT*) of fused silica is also very low (<10^–6^/*K*),^44^ we expect that the change in the transmitted wavefront with respect to the temperature will be low as well. Therefore, we anticipate the impact of thermal variations on the optical performance of all-glass metalens to be insignificant within the given test temperature range. The metalens also does not exhibit noticeable physical damage under the vibrational stress induced by immersing the sample in an ultrasonication bath for 20 min (Figure S23). These results suggest that the all-glass metasurfaces can survive extreme environment conditions and therefore is well-positioned for space applications requiring launch survival. Similar tests conducted on TiO_2_ metasurfaces comprising 700 nm tall nanopillars^45^ also show promising results as well (Figures S24, S25). The thermal resilience also allows various optical coatings to be applied onto the metalens after fabrication since such modifications require special thermal environments during coating.

## Conclusions

In summary, we fabricate an all-glass 100 mm diameter metalens capable of operating in the visible wavelength range using DUV lithography, surpassing the tool’s exposure size limit. We realize a significant number of nanostructures (18.7 billion) in a visible-spectrum metasurface optic while eliminating the sidewall taper with an improved vertical etch process. The diameter of the metalens can be further increased to about 290 mm, as 300 mm diameter wafers and corresponding CMOS foundry tools have become increasingly available in the industry. In addition to PSF measurements, full-aperture interferometry offers valuable insights into the optical wavefront-based characterization of metalenses by decoupling the phase and amplitude error information. Through simulation, we also show the effects of various exposure field-linked systematic fabrication errors on imaging quality, which provide tolerance windows for the manufacturing processes. Additionally, we demonstrate that the single metalens is capable of imaging celestial objects in the visible wavelength range.

Large-area optics, in general, play a crucial role in a wide range of scientific and technological applications. They offer increased light-gathering power, making them suitable for remote imaging and sensing applications in which maximizing the amount of collected light while minimizing the effect of noise is essential. Additionally, large-area optics are useful for directional energy transport by dispersing the energy density and mitigating the risk of structural damage or overheating of the element. We anticipate that the ability to produce large-area metasurface optics using existing CMOS manufacturing processes will augment the inventory of precision large optics available to the optics community.

The efficiency of the presented metalens can be improved by using all-industrial grade reticles and higher resolution manufacturing techniques such as immersion DUV lithography (λ = 193 nm), so that even smaller nanopillars can be fabricated.^9,12^ Dispersion engineering with anisotropic pillar shapes can also be employed to achieve broadband high efficiency.^30^ As fused silica has relatively high laser-induced damage threshold (LIDT) compared to most optical glasses,^46^ we anticipate that the all-glass metasurface platform will be useful in versatile coating options and high-power laser applications not only for focusing but also for polarization manipulation^28^ and pulse compression.^47,48^ Furthermore, their resilience under extreme environmental conditions highlights their suitability for remote imaging in harsh environments. Additionally, the demonstrated fabrication process holds promise for creating large-diameter aberration-correcting meta-optics.^32,33^ These meta-optics may replace or enhance optical components in existing multielement optics systems, providing a path toward low-weight, high-performance, large-aperture imaging devices.

## Materials and Methods

### Fabrication Method

150 mm diameter, double-side polished, 500 μm thick JGS2 fused silica wafers (WaferPro LLC) are used in this study. During the fabrication process, the creation of a photoresist edge-bead, either by recoil or surface tension, can introduce unreliable fabrication conditions. This includes challenges such as difficulties in wafer height detection or achieving plasma etch uniformity at the edge of the wafer. In addition, some plasma etching tools mechanically clamp down on edge of the wafer during processing that the edge of the area should be avoided. Therefore, it is generally a good rule of thumb to exclude a few millimeters around the rim of a wafer during wafer-based manufacturing. As conventional semiconductor foundries typically use 100 mm (4-in.), 150 mm (6-in.), 200 mm (8-in.), or 300 mm (12-in.) wafers, here we choose 150 mm diameter wafer as a substrate for a 100 mm diameter metalens.

The wafers are cleaned using a MOS clean process involving an organic cleaning step by immersing the wafers in 1:1:6 volumetric ratio H_2_O_2_:NH_4_OH:H_2_O solution at 85 °C for 10 min, followed by an ionic clean step by immersing in 1:1:6 H_2_O_2_:HCl:H_2_O solution at 85 °C for 10 min. After each cleaning step, the wafers are thoroughly rinsed in a deionized (DI) water bath by filling and dumping the rinse bath 3 times. The MOS cleaned wafers are then spin-rinsed and dried (Superclean 1600 Spin Rinse Dryer, Verteq). The 150 nm thick Al film is deposited on the cleaned wafers using an e-beam evaporator with planetary wafer holders (CHA MARK 50 E-beam Evaporator, CHA Industries).

The Al-coated wafers are first spin-coated with a 62 nm thick DUV antireflection coating (ARC) layer (DUV 42P, Brewer Science), followed by spin-coating of 600 nm thick positive DUV resist (UV210-0.6, Shipley). The alignment marks are exposed at the designated positions as shown in Figure S3 using a DUV projection lithography tool (PAS 5500/300C DUV Stepper, ASML). The photoresist film is then postexposure baked (PEB) and developed using AZ 726 MIF developer. All spin-coating, baking, and developing processes are performed with a SUSS MicroTech Gamma automatic coat-develop tool for consistent fabrication results. The developed alignment marks are then transferred to the ARC layer using Ar/O_2_ reactive ion etching (RIE, PlasmaLab80Plus, Oxford Instruments), exposing the underlying Al film. Then, the alignment markers are wet-etched into the Al film to a depth of approximately 120 nm to meet the requirement for the phase-contrast detection system in the DUV stepper system. The resist and ARC layer are then stripped using downstream oxygen plasma ashing (YES EcoClean, Yield Engineering Systems).

The wafer with alignment marks (i.e., fiducials) etched into the Al layer is then coated with a 62 nm thick DUV ARC layer, followed by a 500 nm thick layer of negative DUV resist (UVN2300, Dow Electronic Materials). The 100 mm diameter metalens pattern is exposed onto the wafer with the DUV stepper system as depicted in Figure S1, where reticles 1–3 are fabricated by an industry-grade photomask manufacturer and the reticles 4–7 are prepared on chrome coated reticle plates using the DWL2000 Laser Writer (Heidelberg Instruments) followed by developing and chrome wet-etching with the HMP900 Mask Processing System (Hamatech). The DUV photoresists used in this study are chemically amplified resists, hence, it is important to minimize the time between the exposure of the first section and the last to achieve the desired pattern sizes throughout the wafer. In detail, the photoresists used in this study consist of compounds that generate acid upon photoactivation. The shape and size of the developed photoresist profile is determined by the acid-induced reaction that occurs during the postexposure bake step. Since the photogenerated acid diffuses into the photoresist film over time, any significant delay between exposures of the discretized sections can lead to differences in the feature dimensions across those sections. Therefore, reducing the number of sections to be aligned and exposed or the overall time of exposure can help ensure consistency of the feature sizes between the sections. The exposed resist layer then goes through PEB and development processes using the AZ 726 MIF developer solution and the SUSS MicroTech Gamma automatic coat-develop tool. The metalens pattern is then transferred to the ARC layer using Ar/O_2_ RIE (PlasmaLab80Plus, Oxford Instruments).

Using the patterned resist and ARC layer as an etch mask, the metalens pattern is then transferred to Al film using an Ar/Cl_2_ inductively coupled plasma RIE (ICP-RIE, PlasmaPro 100 Cobra 300, Oxford Instruments). The resist and ARC layer are then stripped using downstream plasma ashing (Matrix 105, Matrix Integrated Systems), leaving only the patterned Al on the wafer. We note that the downstream plasma ashing minimally affects the patterned Al layer. Then, the 1.5 μm tall, fused silica nanopillars are formed by vertically etching into the fused silica substrate using C_3_F_8_ ICP-RIE (NLD-570, ULVAC), using the patterned Al as an etch mask. In detail, when C_3_F_8_ is introduced in the etch chamber and is dissociated into plasma, a thin film of fluorocarbon deposits on the exposed area of the substrate, creating an etch inhibitor layer that prohibits fluoride ions from chemically etching SiO_2_ (Figure S5a). The fluoride ions, which are accelerated toward the surface of the substrate, physically bombard and break the fluorocarbon film and chemically etch the SiO_2_ underneath anisotropically. By balancing the growth rate of the fluorocarbon film and the etch speed with temperature, one can control the sidewall tapering angles during the SiO_2_ etch (Figure S5b–i). Details of the vertical etch process is provided in Figures S5 and S6. Then, the residual Al on top of each nanopillar is removed using selective etching with Ar/Cl_2_ ICP-RIE (PlasmaPro 100 Cobra 300, Oxford Instruments).

## Data Availability

Unprocessed and processed astrophotography images are available at DOI: 10.6084/m9.figshare.24531058.

## Supporting Information Available

Supplementary and additional data, including Figures S1–S27 and Tables S1–S3 (PDF)
