# Supplementary material for: All-Glass 100 mm Diameter Visible Metalens for Imaging the Cosmos
Source: ACS Nano. 2024 Jan 17;18(4):3187–98. doi: 10.1021/acsnano.3c09462 (PMC10832996; doi:10.1021/acsnano.3c09462)
Supplement: Supplementary file 1 — nn3c09462_si_001.pdf [file nn3c09462_si_001.pdf]

Supporting Information for

All-glass 100 mm Diameter Visible Metalens for Imaging the Cosmos

Authors: Joon-Suh Park[1]*†, Soon Wei Daniel Lim[1]†, Arman Amirzhan[1], Hyukmo Kang[2], Karlene Karrfalt[2,3], Daewook Kim[2], Joel Leger[3], Augustine Urbas[3], Marcus Ossiander[1,4], Zhaoyi Li[1], and Federico Capasso[1]*

[1]John A. Paulson School of Engineering and Applied Sciences, Harvard University; Cambridge, Massachusetts, 02138, United States.

[2]Wyant College of Optical Sciences, The University of Arizona; Tucson, Arizona, 85721, United States.

[3]Air Force Research Laboratory, Wright-Patterson Air Force Base; Dayton, Ohio 45433, United States.

[4]Institute of Experimental Physics, Graz University of Technology, 8010 Graz, Austria.

†These authors contributed equally to this work.

*Corresponding author. Email: parkj@g.harvard.edu, capasso@seas.harvard.edu

This PDF file includes:

Supplementary Text
Figs. S1 to S27
Tables S1 to S3

Supplementary text:

Generation of metalens design file

In this metalens design file generation, we place the nanopillars in cylindrical coordinates from azimuthal symmetry considerations[1]. The required fused silica nanopillar diameter *D(n)* at each radial position r_n is chosen to satisfy Eq. (1) as closely as possible. The edge-to-edge distance between the nanopillars is fixed at 250 nm. The nth nanopillar, which as a diameter of *D(n)* at each radial position r_n, is then placed evenly along the azimuthal direction so that the angular distance between nanopillars ($\Delta\theta_n$) satisfies the following equation:

$$\Delta\theta_n = \frac{2\pi}{\left\lfloor \frac{2\pi r_n}{D(n)+250\ nm} \right\rfloor}, \qquad \text{(Eq. S1)}$$

where $\lfloor x \rfloor$ is the floor function. This places nanopillars so that they have the same edge-to-edge distance equal to or slightly larger than 250 nm. This resulted in placement of 18,700,751,972 nanopillars for the entire 100 mm diameter metalens. The azimuthal spacing between nanopillars is slightly larger than 250 nm close to the metalens center due to the larger quantization error associated with the small integer number of nanopillars; however, such changes to the edge-to-edge gap have minimal effect on the transmitted phase from each nanostructure (see Fig. S2(b)). The maximum phase error resulting from such edge-to-edge spacing variation is 0.26 radians or 0.04 λ, which is below the Maréchal criterion of 0.25 λ for achieving diffraction-limited performance[2–4].

The created 100 mm diameter metalens design is then discretized into 25 sections, each having an area of 20 × 20 mm. Out of the 25 sections, only 7 sections (see Fig. S1) are selected and written to individual GDSII format CAD files using the GDSPY module on Python. We use 1 nm as the coordinate precision. Several techniques are used to reduce each CAD file size so that it can be later loaded to commercial photomask writers: (1) Each nanopillar with a specific diameter is only created once as a GDS cell, and other positions reference that first cell. (2) Each circle that represents a nanopillar is approximated to be an octagon to reduce the number of vertices. Nanopillars that are within 250 nm distance from the reticle boundaries are removed to avoid structure overlap between the sections. The total file size for all 7 reticle designs is 115.6 GB (19 GB, 20 GB, 20.3 GB, 20.1 GB, 16.5 GB, 3.2 GB, and 16.5 GB for reticles #1-#7, respectively), and all GDSII files are generated simultaneously using a 20-core workstation (two

Intel® Xeon® E5-2690 v2 processors, 768 GB RAM) with a peak memory usage of 500 GB, taking less than 5 hours.

Simulation of metalens performance

The 100 mm metalens is numerically simulated by discretizing the 100 mm aperture into 100,000 annular rings with equal radial thickness of 500 nm, then discretizing each annular ring into 100 equal sections in the azimuthal direction, which produces 10 million annular sections. The radial discretization is chosen to resolve the highest spatial frequency zone at the edge of the metalens using at least four points. Each of the 10 million annular sections is associated with the complex scalar electric field that is transmitted through the nanopillar located at the geometric center of that annular section. The transmitted electric field through each nanopillar is obtained using the locally periodic assumption[5]. Each glass nanopillar on glass substrate geometry combination is individually simulated using the Rigorous Coupled Wave Analysis platform RETICOLO[6] on a periodic square lattice with a nominal edge-to-edge distance of 250 nm, 200 staircase discretization per circular arc quadrant, and 2601 Fourier plane waves. Empty regions without nanopillars are simulated under the same conditions with the nanopillar being replaced with a layer of air that has the same thickness as the nanopillar height. The fused silica refractive index as a function of wavelength is obtained from the dispersion equation published by Malitson[7]. The transmitted electric field associated with each nanopillar is the zeroth diffraction order electric field for normal incidence illumination from the glass substrate. The area-weighted electric fields are propagated to the focal plane using a vectorial propagator[8]. The focal plane is sampled on a 201 × 201 square grid up to a maximum transverse diameter of 6 Airy disk diameters, where one Airy disk diameter is $1.22\lambda/\mathrm{NA}$. The focusing efficiency is defined as the power flux through a circular diameter equal to 3 Airy disk diameters at the focal plane, divided by the incident power at the metalens. This focusing efficiency does not include the Fresnel reflection loss at the first air/glass interface on the back face of the metalens, which is expected to be around 4%. The 2D modulation transfer function (MTF) of the focal spot is computed by Fourier transformation of the intensity profile within 6 Airy disk diameters on the focal plane with no zero padding. The Strehl ratio of the focal spot is calculated by integrating the volume under the 2D MTF and dividing it by the volume under the diffraction limited 2D MTF.

**Publish Date (YYYY. MM.)**	**Diameter (λ)**	**Diameter (mm)**	**λ (nm)**	**Wavelength range**	**Fabrication Method**	**Material**	**Focal length (mm)**	**NA**	***f*/#**	**Pol.-dep. (Y/N)**	**Focusing Eff.**	**Ref.**
1996. 02.	1,580	**1** (square)	632.8	Visible	EBL	Fused quartz	20	0.025	20	N	53%	9
1999. 05.	233	**0.2** (square)	860	NIR	EBL	TiO_2	0.4	20° off axis		N	80%	10
2012. 08.	581	**0.9**	1550	NIR	EBL	Ag	30, 60	0.015, 0.075	33.3, 66.7	Y	1%	2
2013. 04.	6<br>10<br>14	**0.004**<br>**0.007**<br>**0.0094**	676	Visible	FIB	Au	0.025<br>0.005<br>0.007	0.62<br>0.57<br>0.56	0.63<br>0.710<br>.74	Y	10%	11
2014. 12.	10	**305** (square)	30×10^6 (10 GHz)	GHz	Not reported	Metal	300	0.58	0.70	Y	24.7%	12
2016. 06.	451	**0.24**	532	Visible	EBL	TiO_2	0.09	0.80	0.38	Y	73%	13
2017. 10.	75	**0.3**	4000	MIR	EBL	a-Si:H on MgF_2	0.05	0.95	0.17	N	78%	14
2017. 10.	104	**10**	96×10^3 (3.11 THz)	THz	Photolith.	SOI	30	0.16	3.00	N	24%	15
2018. 01.	12,903	**20**	1550	NIR	*i*-line stepper	a-Si	50	0.20	2.50	N	91.8%	16
2018. 02.	839	**0.6**	715	NIR	EBL	a-Si	0.042	0.99	0.07	N	37%	17
2018. 02.	3,871	**6**	1550	NIR	*i*-line stepper	a-Si	50	0.06	8.33	N	62.5%	18
2018. 02.	571 -870	**0.4**	460 -700	Visible	EBL	TiO_2 on SiO_2/Ag	0.98	0.20	2.45	N	16-22.6%	19
2018. 04.	192	**1**	5200	MIR	EBL	PbTe on CaF_2	0.5	0.71	0.50	Y	75%	20
2018. 07.	15,798	**10**	633	Visible	EBL	SiN	-4	0.78	-0.40	N	*Not reported*	21
2018. 11.	30,303<br>37,594<br>42,283	**20**	660,<br>532,<br>473	Visible	EBL	poly-Si	12.9,<br>16,<br>18	0.61,<br>0.53,<br>0.49	0.65,<br>0.8,<br>0.9	Y	79%	22

**Publish Date (YYYY. MM.)**	**Diameter (λ)**	**Diameter (mm)**	**λ (nm)**	**Wavelength range**	**Fabrication Method**	**Material**	**Focal length (mm)**	**NA**	***f*/#**	**Pol.-dep. (Y/N)**	**Focusing Efficiency**	**Ref.**
2019. 08.	2,000 1,200 750	**6** (square)	3000, 5000, 8000	MIR	EBL	Ge	2	0.83	0.33	Y	33%	23
2019. 09.	2,128	**10**	4700	MIR	EBL	Au	120	0.04	12.00	Y	*Not reported*	24
2019. 11.	15,798	**10**	633	Visible	KrF DUV	Fused silica	50	0.10	5.00	N	45.6%	1
2019. 11.	5,639	**3**	532	Visible	EBL	TiO_2	14.4	0.10	4.80	N	*Not reported*	25
2020. 01.	2,128	**2**	940	NIR	ArF immersion DUV	a-Si	1.732	0.50	0.87	N	29.2%	26
2020. 03.	18,797	**10**	532	Visible	EBL	Resist (ma-N)	4	0.78	0.40	Y	80%	27
2020. 05.	19 -30	**6.48**	0.2×10^6-0.3×10^6 (0.9 - 1.4 THz)	THz	Photolith.	Si	15	0.21	2.31	Y	33.9%	28
2020. 09.	1,000	**5.2** (square)	5200	MIR	EBL	PbTe on CaF_2	2.5	0.88	0.27	N	32-45%	29
2021. 01.	4,098 3,759 3,040	**2**	488, 532, 658	Visible	EBL	TiO_2	3.2, 1	0.3 0.7	0.5 1.55	Y	*Not reported*	30
2021. 02.	288	**1.5** (square)	5200	MIR	EBL	GSST	1.5, 2	0.58, 0.47	0.71, 0.94	Y	23.7%, 21.6%	31
2021. 04.	581	**0.9**	1550	NIR	NIL	Si	10	0.04	11.11	Y	26%	32
2021. 08.	7,273	**4**	550	Visible	NIL	TiO_2	9.8	0.20	2.45	N	43%	33
2021. 10.	1,290	**2**	1550	NIR	EBL	a-Si	4	0.24	2.00	N	42.7%	34
2022. 01.	3,160	**2**	633	Visible	EBL	sc-Si	2	0.45	1.00	N	64%	35
2022. 04.	4,887	**2.6**	532	Visible	EBL	GaN	5.03	0.25	1.93	N	*Not reported*	36

Publish Date (YYYY. MM.)	Diameter (λ)	Diameter (mm)	λ (nm)	Wavelength range	Fabrication Method	Material	Focal length (mm)	NA	*f*/#	Pol.-dep. (Y/N)	Focusing Eff.	Ref.
2022. 05.	20,492 18,797 15,198	**10**	488, 532, 658	Visible	EBL	TiO_2	16	0.30	1.60	N	15%	37
2022. 05.	55,172	**80**	1450	NIR	KrF DUV	a-Si	260	0.15	3.25	N	80.8%	38, 39
2022. 05.	4,717	**50**	10600	LWIR	Stepper photolith.	Si	34	0.59	0.68	N	*Not reported*	40
2022. 08	8,000	**80**	10000	LWIR	Contact photolith.	a-Si	80	0.45	1.00	N	*Not reported*	41
2023. 01	4,887	**2.6**	532	Visible	EBL	GaN	10	0.13	3.85	N	74%	42
2023. 03	22,222 18,797 15,748	**10**	450, 532, 635	Visible	NIL, ArF immersion DUV	TiO_2 coated resin	24.5	0.20	2.45	Y	40.9%, 55.6%, 44.6%	43
**This work**	157,978	**100**	633	Visible	KrF DUV	Fused silica	150	0.32	1.50	N	40.4%	

Table. S1. List of notable metalens' diameters and their parameters.

Progression in time of notable metalens diameters with respect to design wavelength (λ and mm), fabrication methods, constituent materials, polarization dependence (pol.-dep.), and their optical parameters (focal length, numerical aperture (NA), f-number (*f*/#), and focusing efficiency. Acronyms in the fabrication method column are as follows:

- EBL: Electron beam lithography
- NIL: Nano-imprint lithography
- FIB: Focused-ion beam lithography
- DUV: Deep-ultraviolet lithography

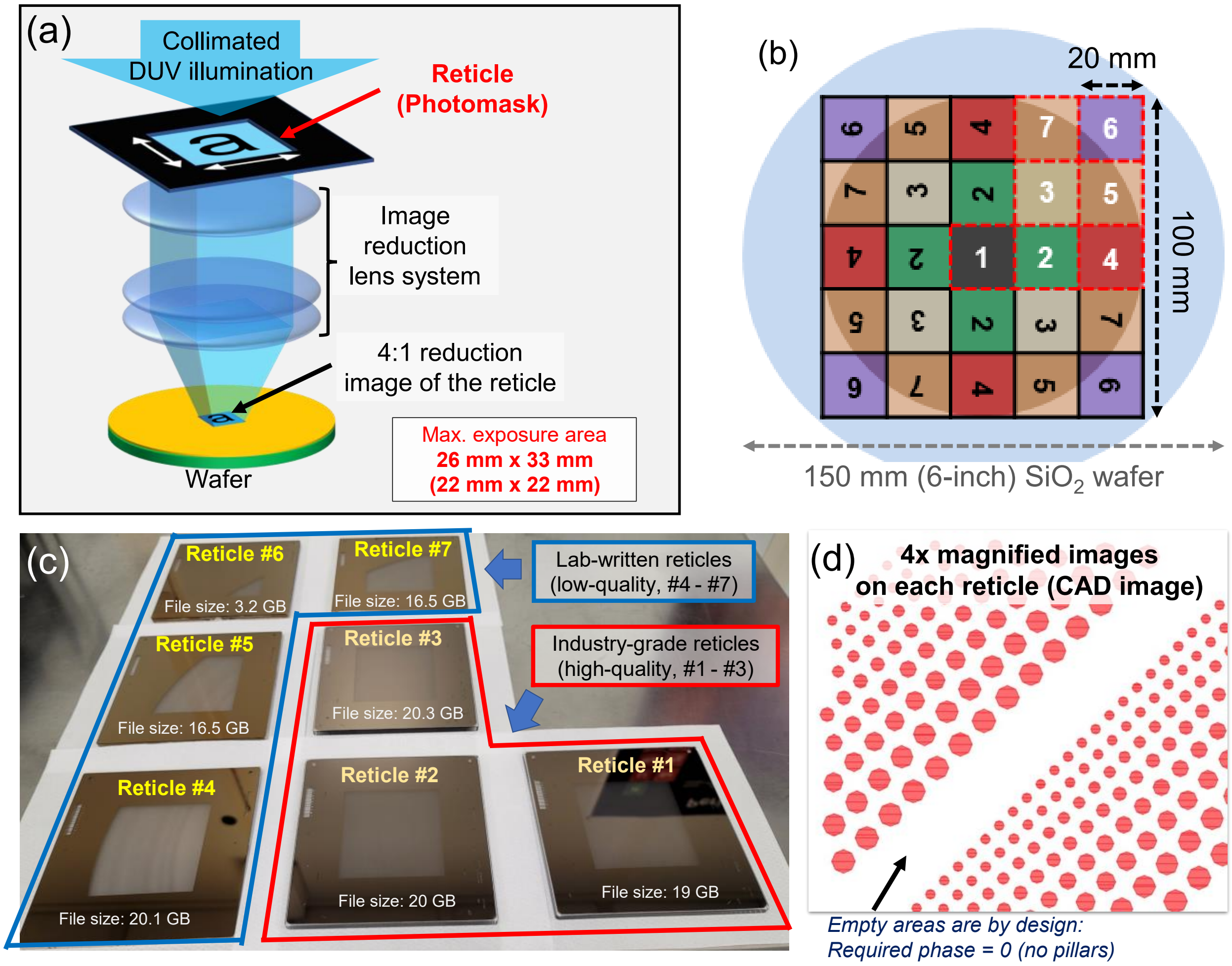

Fig. S1. Fabrication strategy and prepared reticles.

(a) An abbreviated schematic of a DUV projection lithography system. A DUV beam (λ=*248 nm* for KrF excimer laser source) is incident on a reticle (photomask) which has the desired patterns written in chrome. The transmitted light is then passed through an array of image reduction lenses so that the image on the reticle is demagnified and projected onto the surface of a target wafer. Depending on the lithography tool, the demagnification ratio is typically 4:1 or 5:1. This allows the resolution of features smaller than the source wavelength, while keeping the reticles pristine. These image reduction systems, however, limit the maximum exposure area with guaranteed image resolution only on the order of *30 mm* square area on the wafer. In this article, we use an ASML PAS 5500/300C DUV stepper having λ=*248 nm*, demagnification ratio of 4:1, and maximum exposure area of $22 \times 22\ mm$ on a wafer. (b) To realize a 100 mm diameter metalens using the DUV system, we discretize the lens into 25 different sections, each having a $20 \times 20\ mm$ area. We intentionally put reticle #1 at the center of the metalens pattern to avoid stitching errors on the optic axis. As the metalens is azimuthally symmetric, we identify and use only the 7 unique sections from a quadrant to make the whole metalens. As we target a 100 mm (4-in.) diameter metalens, we choose the wafer to be larger than the metalens diameter (150 mm, 6-in.) which allows space for alignment markers and easier handling. (c) Fabricated reticles for the 100 mm metalens. Reticles #1-#3 (inner 60 × 60 mm area) are fabricated by industry-grade

photomask manufacturer (Photronics Inc.) and reticles #4-#7 are fabricated in-house using a Heidelberg DWL2000 laser mask writer. Reticles #4-#7 have poorer quality (unresolved small patterns, incorrect feature sizes) compared to reticles #1-#3 due to imperfection in the optimization of in-house mask fabrication processes. The prepared CAD file size (GDSII format) is marked on each reticle. (d) Image of the reticle patterns on a top left edge of the reticle #1. Filled areas (red) represent cleared areas on the reticle, while the unfilled (white) area is opaque with chrome mask. Empty areas along the azimuth are part of the design that contributes to the wavefront. The circular patterns are approximated as octagons to reduce the number of vertices, and therefore reduce the overall CAD file size. The patterns are 4X magnified on the reticle. Structures that overlap with the reticle boundaries are intentionally omitted to avoid multiple exposure between the reticles.

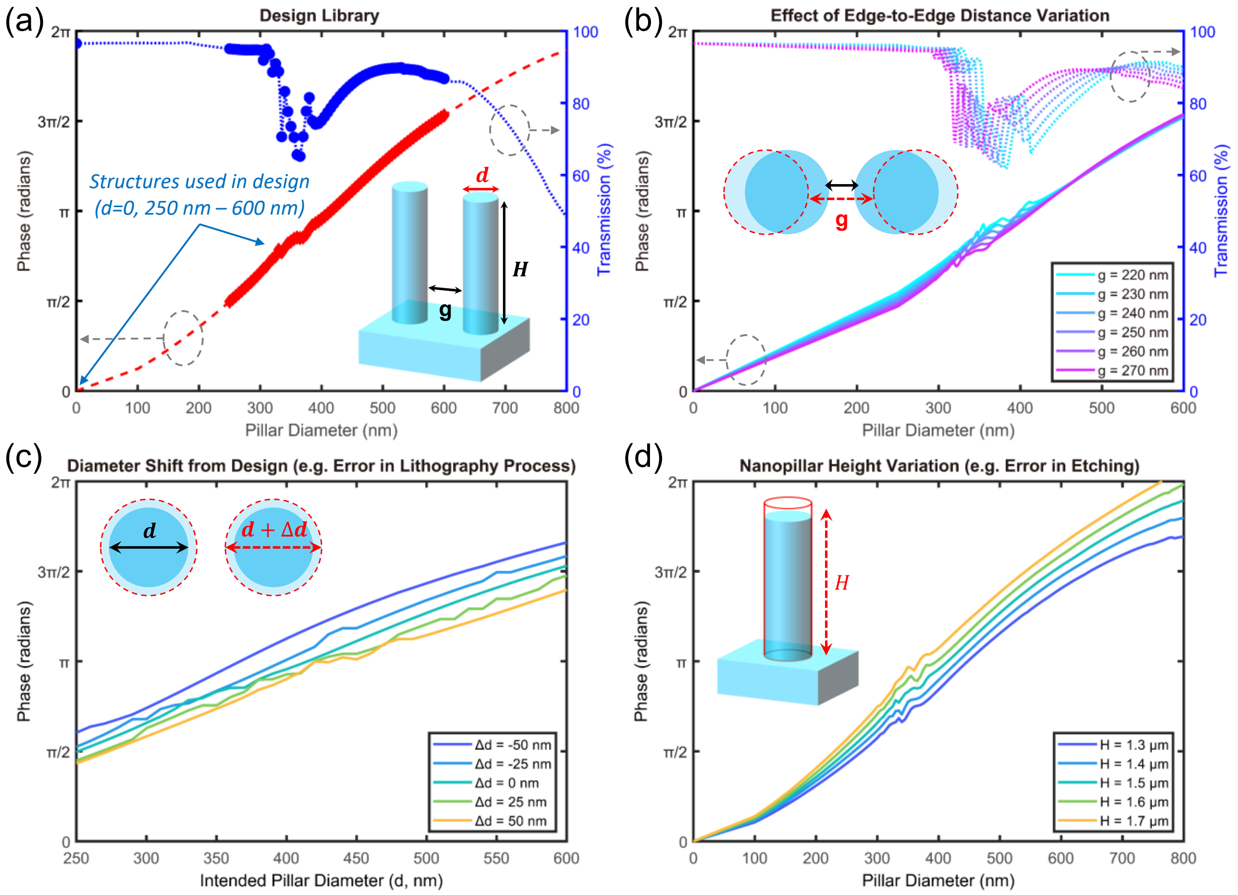

Fig. S2. Simulated library of fused silica nanopillars.

(a) Library of nanopillars used in the metalens design. Nanopillar height H=$1.5\ \mu m$ and edge-to-edge gap g=250 nm is used for while sweeping the diameters from 0 to 800 nm. Only the structures with diameters between 250 nm and 600 nm, and 0 nm (empty area) are selected to be included in the metalens design. (b) Effect of edge-to-edge gap variance on the nanopillar's transmitted phase and intensity. (c) Effect of diameter shift while maintaining the same center-to-center distance to model the size error which could occur during fabrication. (d) Effect of overall nanopillar's height shift on the transmitted phase to model the height error which could occur during fabrication.

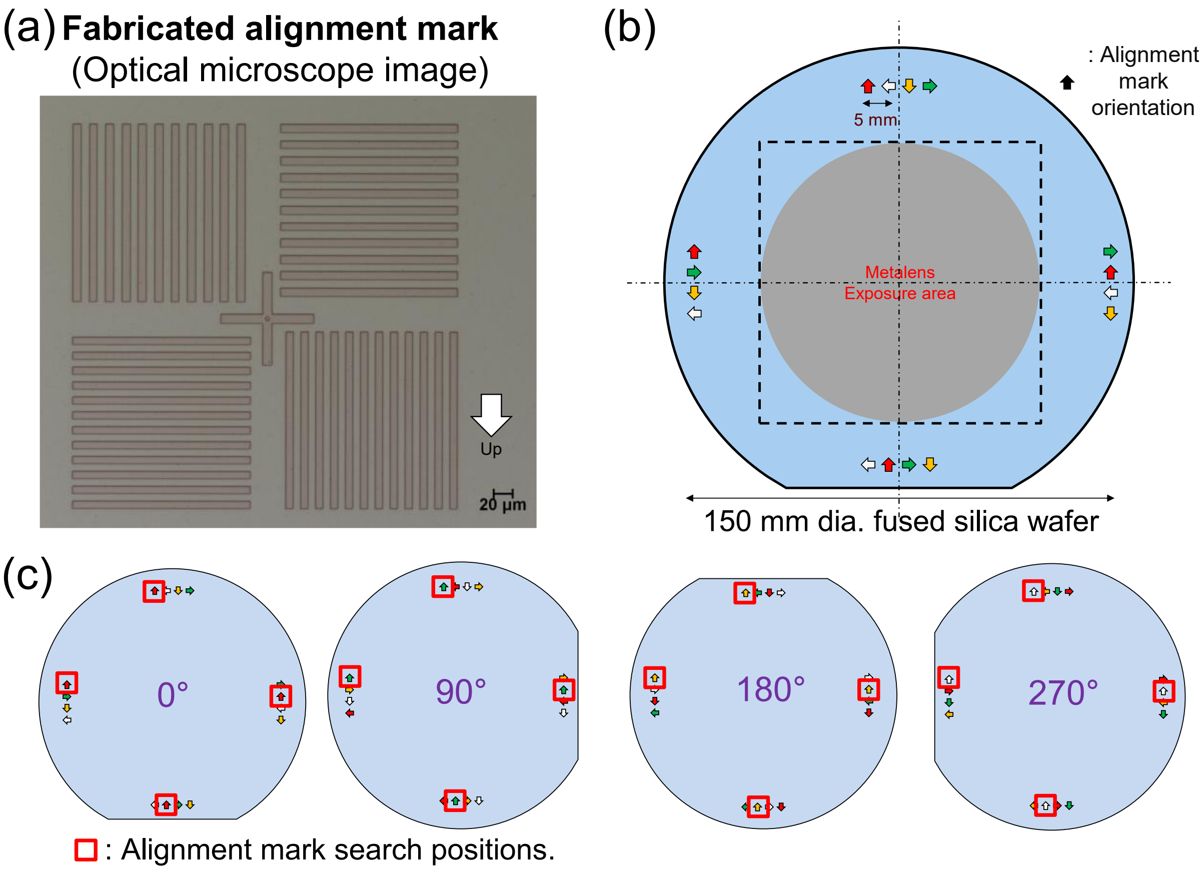

Fig. S3. Rotation and alignment strategy.

(a) Optical microscope image of the alignment marks for the ASML PAS5500/300C DUV stepper. After coating DUV-42P ARC (Brewer Science, anti-reflection coating) and UV210 resist (Shipley, positive DUV resist), the alignment patterns are exposed on the 150 nm thick aluminum film on a 150 mm diameter fused silica wafer using the DUV stepper system (Fig. 1(a)). After post-exposure bake and development of the alignment marks, the patterns are transferred to the Al film using wet etching. The alignment marks are etched to 120 nm in depth to satisfy the phase-contrast detection system in the DUV stepper. (b) Schematic of alignment mark placements. An arrow indicates a mark's position, and the orientation of the mark at each position is indicated by the direction of the arrow. The marks are placed outside the metalens exposure area and are used as global alignment keys during the projection lithography processes for each reticle. The marks are placed in a way that the DUV tool can search the same location for the four alignment marks at each wafer rotation of 0°, 90°, 180°, and 270°, respectively, for the convenience in exposure schedule programming. All alignment and exposure processes are determined and processed by the stepper control computer according to the pre-programmed exposure scheduling.

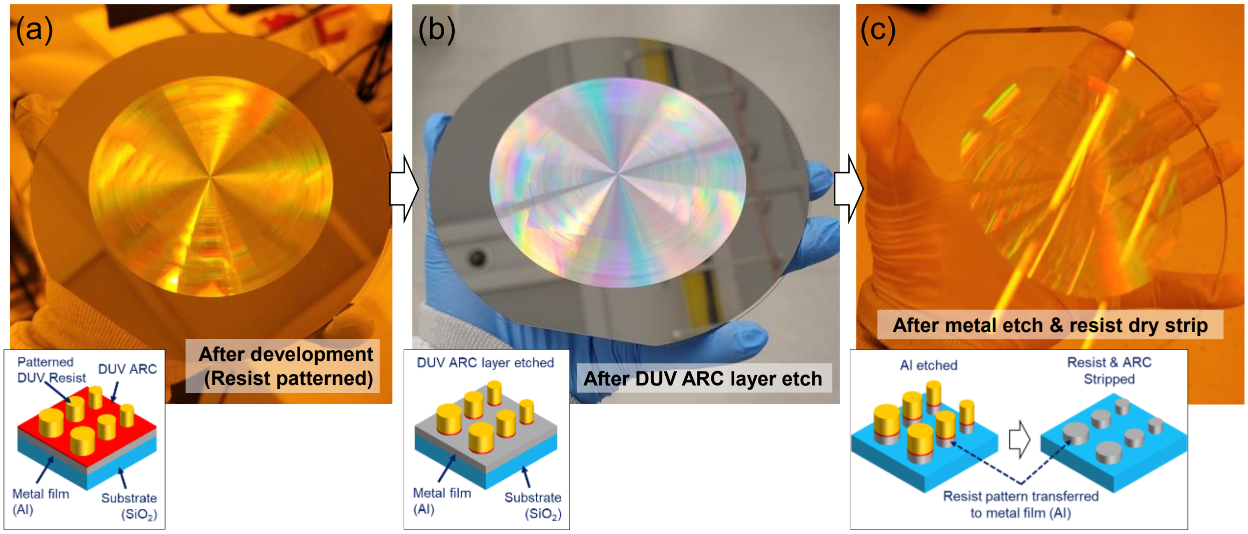

Fig. S4. Photographs of metalens wafer in between fabrication steps.

(a) Photograph of the developed photoresist pattern of the 100 mm metalens. The pattern is formed on the aluminum (Al) film on a 150 mm (6-inch) fused silica wafer, coated with DUV antireflective film (DUV ARC). (b) Using O_2/Ar plasma, the DUV ARC layer is etched so that the metalens pattern is transferred to the ARC layer and expose the Al film below. (c) The 100 mm metalens pattern is then transferred to the Al film using Cl_2 reactive ion etch. The Al on the edge of the wafer is not etched due to the wafer clamping mechanism of the plasma etcher. The residual resist and ARC materials are stripped using downstream plasma ashing.

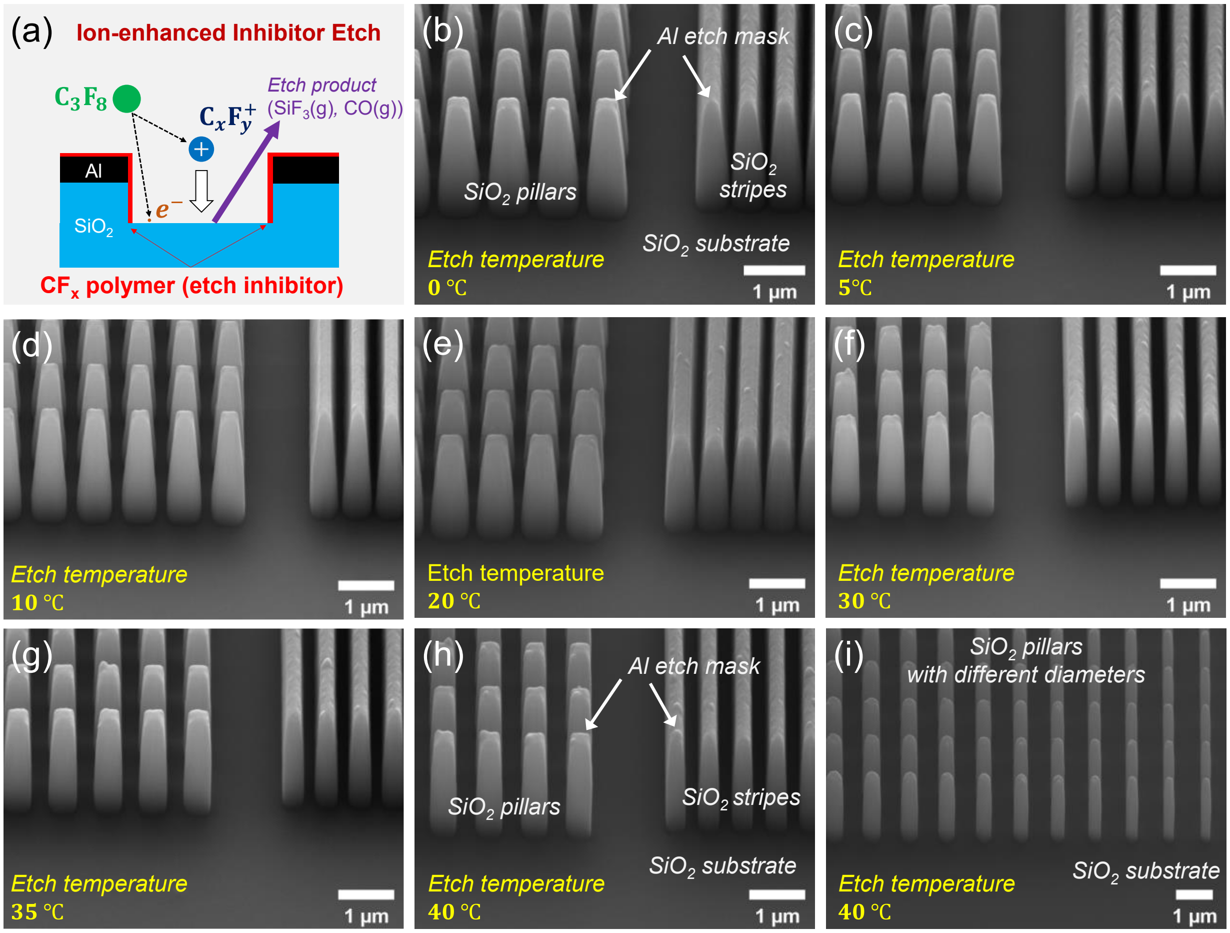

Fig. S5. Optimization of fused silica vertical etching process.

(a) Schematic of ion-enhanced inhibitor etch process. C_3F_8 gas is introduced into the etch chamber and is ionized by RF drive. Light-weight, free electrons are first attracted toward the electrically isolated wafer surface and charge the surface to negative polarity as the electron population is built up. The ionized gas simultaneously forms fluorocarbon polymer on the exposed surface, creating a chemical etch inhibiting film, while the positively charged fluorocarbon ions are accelerated toward the negatively charged surface. The accelerated ions directionally bombard the surface, breaking and removing the fluorocarbon polymer film that faces the surface normal of the substrate. The fluoride radicals present in the plasma diffuse onto the surface and chemically etch the exposed SiO_2, creating volatile SiF_3 and CO as etch products. The polymers deposited on the sidewalls inhibit the lateral etch of SiO_2. The vertical etch of SiO_2 is achieved by balancing the rate of fluorocarbon deposition and the reactive ion etch. Here, we use substrate temperature as the rate control parameter. (b)-(h) Tilted SEM images of the etched SiO_2 pillars and trenches at different etch temperatures. The fixed process temperature for each sample ranges from 0°C to 40°C. As the temperature rises, the polymer deposition speed is slowed and the reactive ion etching is accelerated, that near-vertical sidewalls are reached at 40°C. The samples in the SEM images still have residual Al etch mask on the top of each structure, which accounts for the rough top surface. (i) Tilted SEM image of the vertical-sidewall sidewall etched SiO_2 nanopillars with different diameters. The height of the nanopillars is 1.5 μm. The smooth area at the bottom of the pillars is an etched surface.

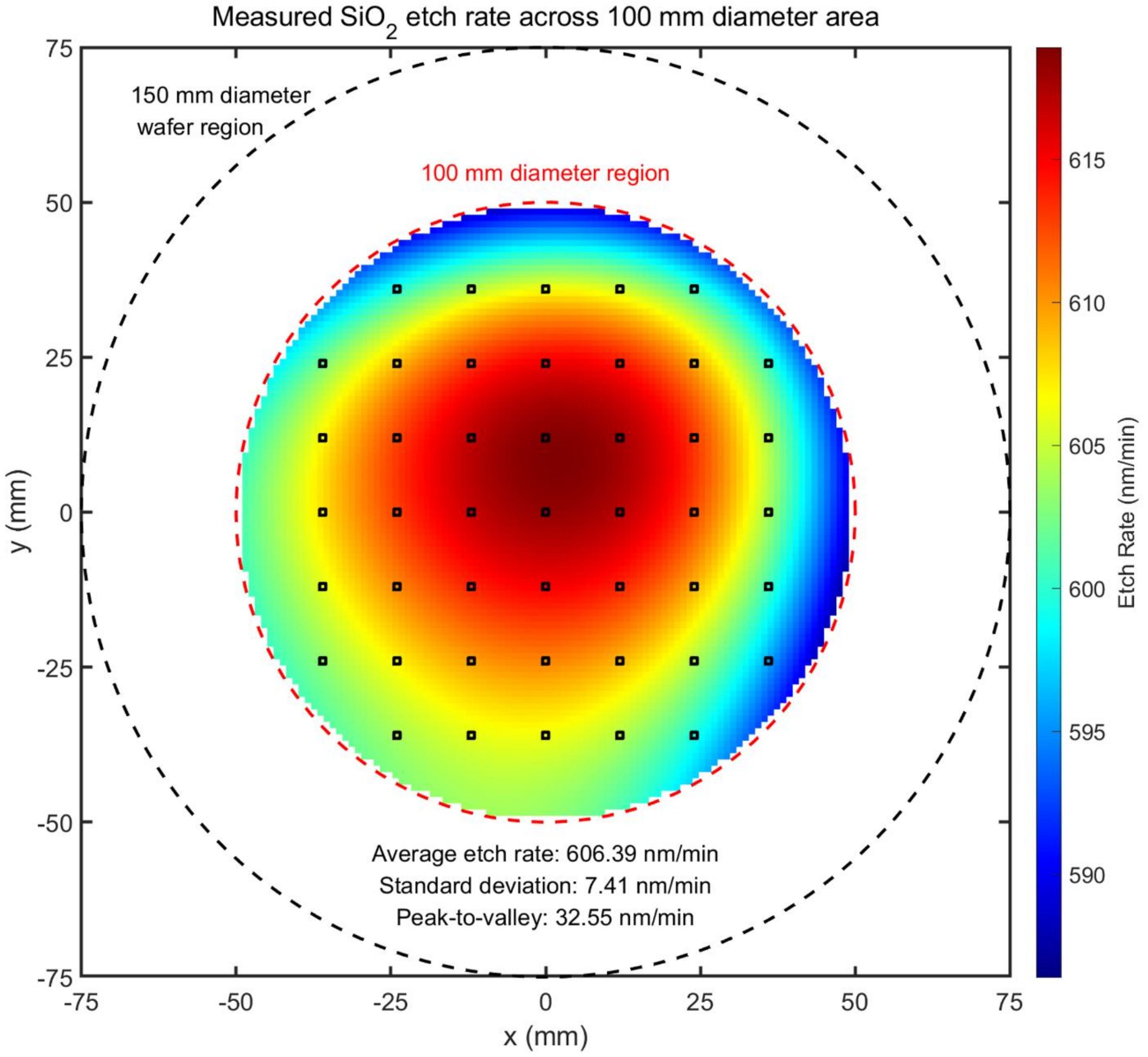

Fig. S6. Measured etch rate uniformity across the 100 mm diameter region.

The SiO_2 etch rate is measured across 100 mm diameter region with stylus profilometry at 45 different positions. The positions of the acquired data are marked in small green squares. With the measurement data, the etch rate across the region of interest is obtained by fitting to a cubic polynomial:

$$f(x,y) = a_0 + a_1x + a_2y + a_3xy + a_4x^2 + a_5y^2 + a_6x^3 + a_7x^2y + a_8xy^2 + a_6y^3.$$

The measured average etch rate across the 100 mm diameter region is 606.4 nm/min, with a standard deviation of 7.41 nm/min. The etch uniformity could be further improved by having better control over the temperature distribution and plasma uniformity in the etch chamber.

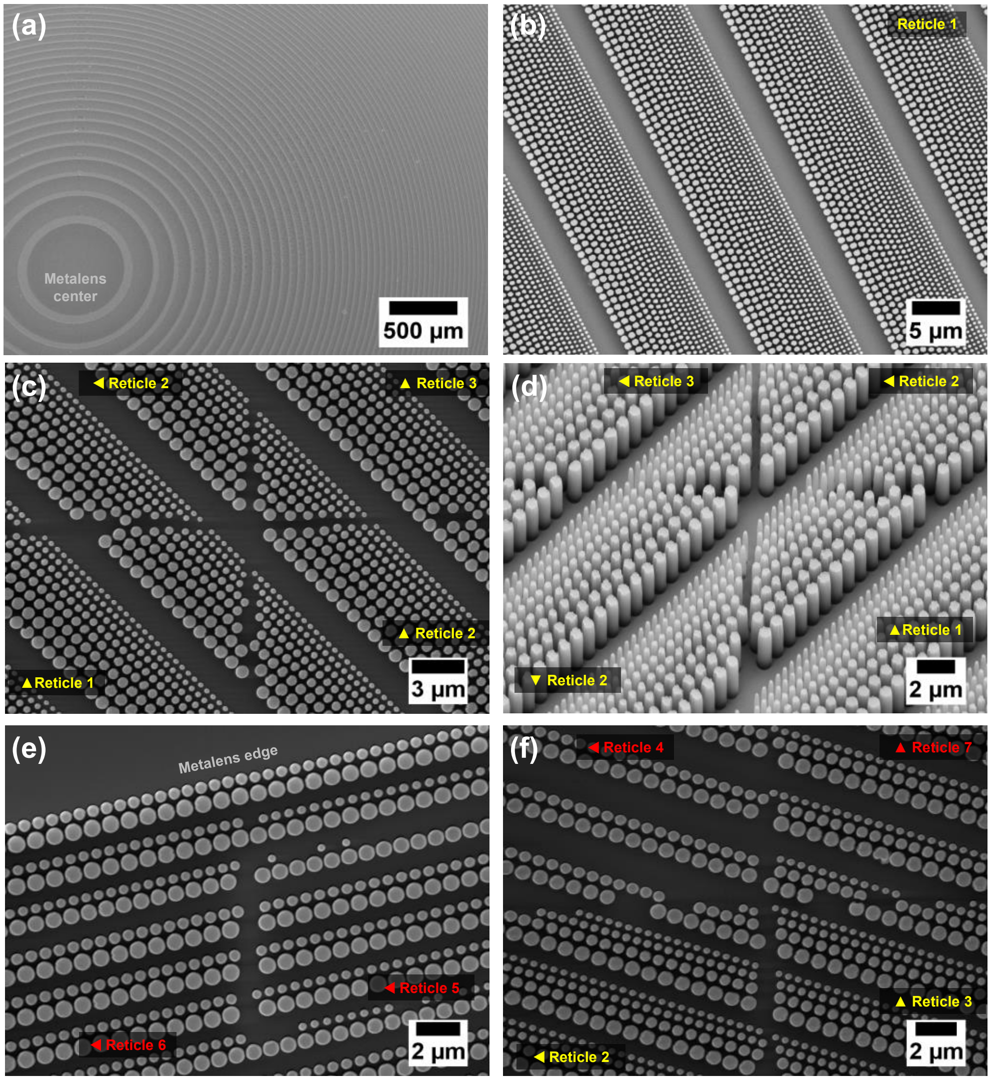

Fig. S7. SEM images of the fabricated 100 mm diameter all-glass metalens.

(a) Top-view SEM image of the metalens center area, corresponding to Reticle #1. (b) Top-view SEM image of the metalens. (c) Top-view SEM image of the metalens where 4 reticles meet; 3 reticles are oriented north, while reticle #2 (top left) is oriented toward west. The missing pillars between the reticles are intentional; they were omitted to avoid possible multiple exposure at the boundary of the sections. (d) Tilted SEM image of metalens where 4 industry-grade reticles meet; reticle #1 is oriented north, while the top two reticles (#2 and #3) are facing west, and lower left corner reticle (#2) is facing south. Note that the vertical sidewalls are consistent for different pillar diameters. (e) Top-view SEM image of the metalens at its edge, between the boundaries of home-made reticles #6 and #5. The missing small pillars are from fabrication error of the reticles, where small pillar structures were not resolved. (f) Top-view SEM image of the metalens where home-made (#4, #7) and industry-grade reticles (#2, #3) meet. Comparing with reticles 2 and 3, reticles 4 and 7 shows slight misalignment, missing small pillars, and incorrect pillar size, originating from the lower-resolution mask writing process developed in-house.

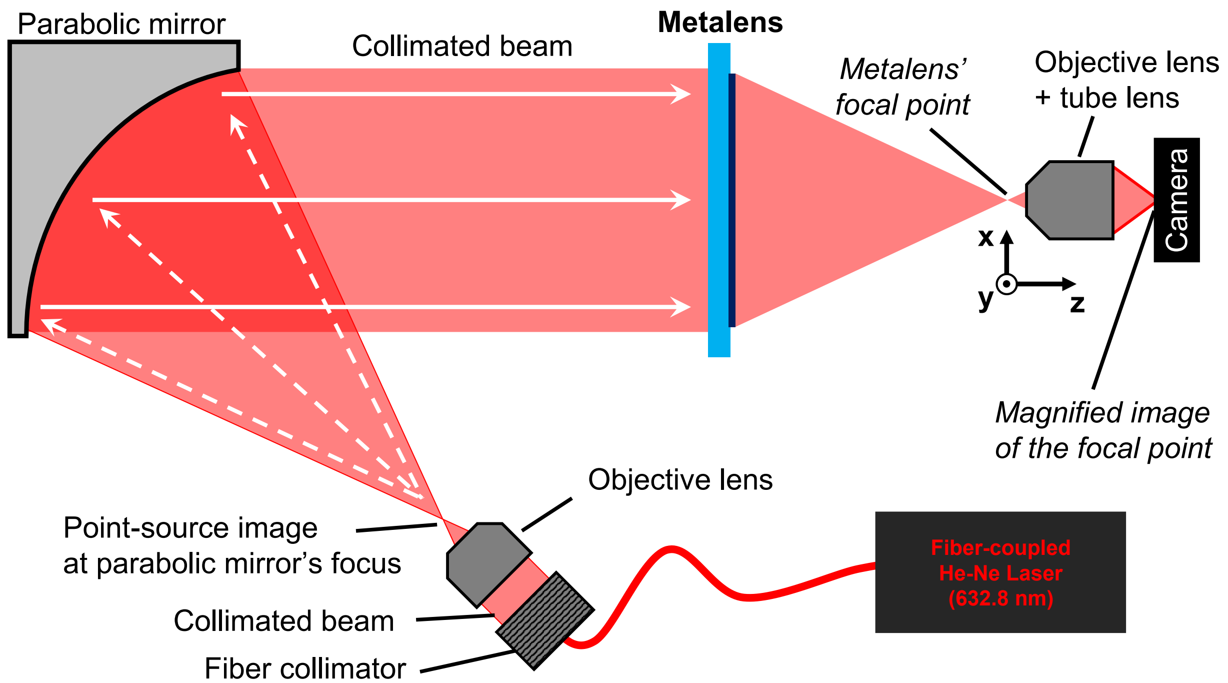

Fig. S8. Schematic of point-spread-function measurement setup.

A fiber-coupled He-Ne laser ($\lambda = 632.8\,nm$, N-LHR-121, *Newport Corporation*) is used as an illumination source. After passing the laser beam through the fiber collimator (F220APC-633, *Thorlabs*), the collimated beam is then sent through an objective lens (EA achro 20X/0.40, W.D. 0.8 mm, *Motic*) from its back side. The focal point of the objective lens is used as the point-source image, which is placed at the focus of a 45° off-axis parabolic mirror (#35-629, *Edmund Optics*). The parabolic mirror then creates a collimated beam with diameter of 101 mm, which is incident on the 100 mm diameter metalens. The image of the focus is 40X magnified and recorded by the microscope camera of Optikos MTF measurement tool (LensCheck, *Optikos Corp.*). The microscope camera is stepped along z-axis to acquire the point-spread-function along the optic axis. The focal plane of the metalens is determined by the plane of maximum on-axis intensity.

For an accurate PSF measurement, a high-quality collimated light incident on a metalens at a normal angle is important. This is relatively easier to achieve for few millimeter aperture optics, where quality optomechanical components are readily available, but not for a hundred-millimeter or larger scale optics: Not only is the creation of large-diameter collimated beam with near-planewave phase front difficult and costly, the alignment of large optics with respect to the collimated beam also requires extensive trial and error. To ensure the validity of the PSF characterization, therefore, a different measurement technique is needed to corroborate the PSF analysis for large-diameter optics, in general. A more commonly used and widely accepted characterization method for large optics is interferometric analysis.

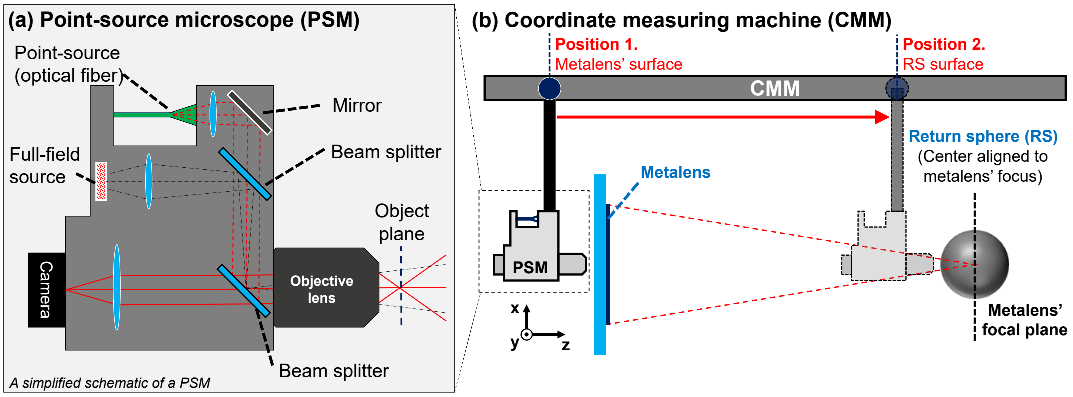

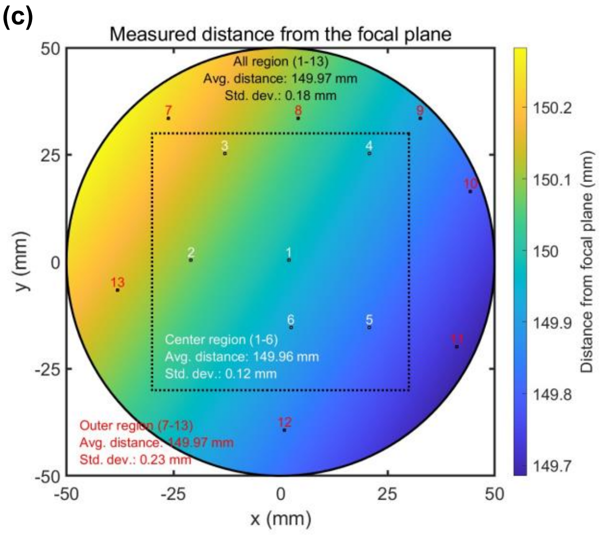

(d)

Center region		Outer region	
#	Measured distance (mm)	#	Measured distance (mm)
1	149.808	7	150.189
2	150.092	8	150.209
3	150.028	9	149.882
4	150.013	10	149.736
5	149.972	11	149.737
6	149.824	12	149.824
		13	150.247

Fig. S9. Focal length measurement using point-source microscope (PSM) and coordinate measurement machine (CMM).

(a) A schematic diagram of a point-source microscope (PSM, *Optical Perspectives Group LLC*). A point source image is projected onto the objective lens' object plane, which is used as a reference to ensure the PSM is correctly focused on the surface of interest or at the center of a sphere. Detailed descriptions and discussions can be found in Ref. 44. (b) Schematic of the focal length measurement apparatus. A PSM is securely mounted on a coordinate measurement machine (CMM, *Tesa Micro-hite 3D, Tesa Technology*), which is first focused on the rear surface of the metalens. After acquiring the x,y,z positions of the PSM at various metalens surface positions, the PSM is then moved to focus on the center of the return sphere (RS), whose center is aligned to the metalens' focus using a Fizeau interferometer. The known thickness of the metalens substrate is subtracted out. (c) Various points of measurements at the surface of the metalens. The positional data are taken at 13 different locations and are labeled with numbers: 1-6 for the inner section of the metalens, and 7-13 for the outer section of the metalens. The measured distance from the focal plane (*i.e.*, metalens' surface to the return sphere's center of curvature) is 149.97 mm with standard deviation of 0.18 mm, which is in good agreement with the designed focal length of 150 mm. (d) List of measured axial (i.e., along the metalens' surface normal) distance values from the return sphere's center of curvature at different positions of the metalens.

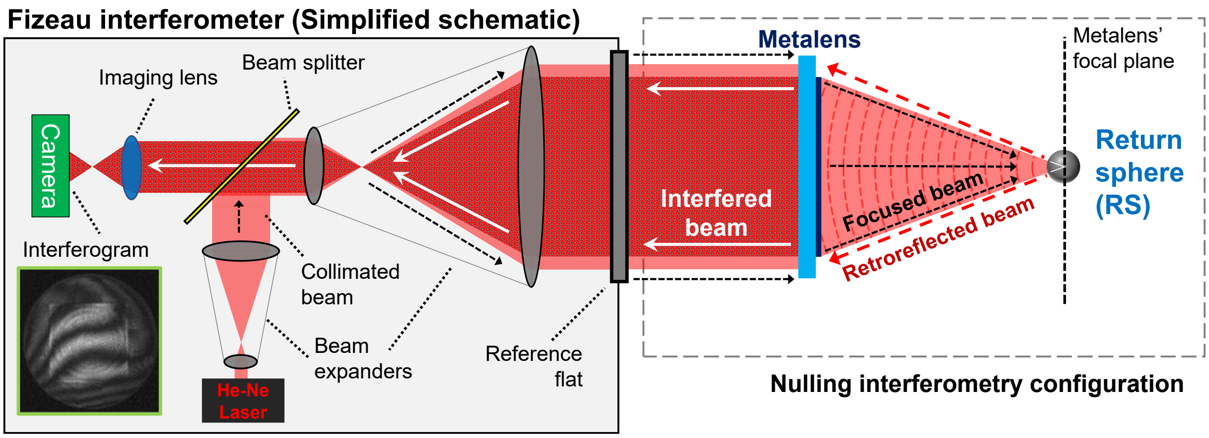

Fig. S10. Schematic of full-aperture interferometry measurement setup.

A laser Fizeau interferometer (Zygo Verifire^{TM} ATZ, *Zygo Corporation*) was used in nulling interferometry double-pass configuration to obtain the full-aperture wavefront measurement. We note that the image depicted on the left is not an exact configuration of the used interferometer but is only a simplified schematic diagram to illustrate the measurement concept. For better understanding, please see Ref. 44. The metalens is illuminated by a collimated beam ($\lambda = 632.8\, nm$) from a Fizeau interferometer, creating the metalens' focus at its focal plane. A return sphere (RS) with a known diameter is placed at the center of the metalens' focus, having the focal plane coincide with the center of the return sphere. The focused beam by the metalens propagates toward the return sphere and is retroreflected from the RS surface, which is re-incident on the metalens. The doubly transmitted (*i.e.*, double-pass) wavefront creates twice of the wavefront aberration function (WAF) deviating from an ideal spherical wavefront; if the wavefront from the metalens is perfectly spherical, the reflected wavefront from the return sphere would also be perfectly spherical, resulting in a null interferogram. The interfered irradiance image is then acquired with a camera. Multiple phase-shifted fringe patterns are used to calculate the phase map. The phase map is unwrapped across the full-aperture to provide the wavefront error map. After removing artifacts from misalignment (*i.e.*, piston, tip, tilt, and power) of the metrology configuration (*i.e.*, interferometer, metalens, and the return sphere) we obtain the metalens' wavefront error.

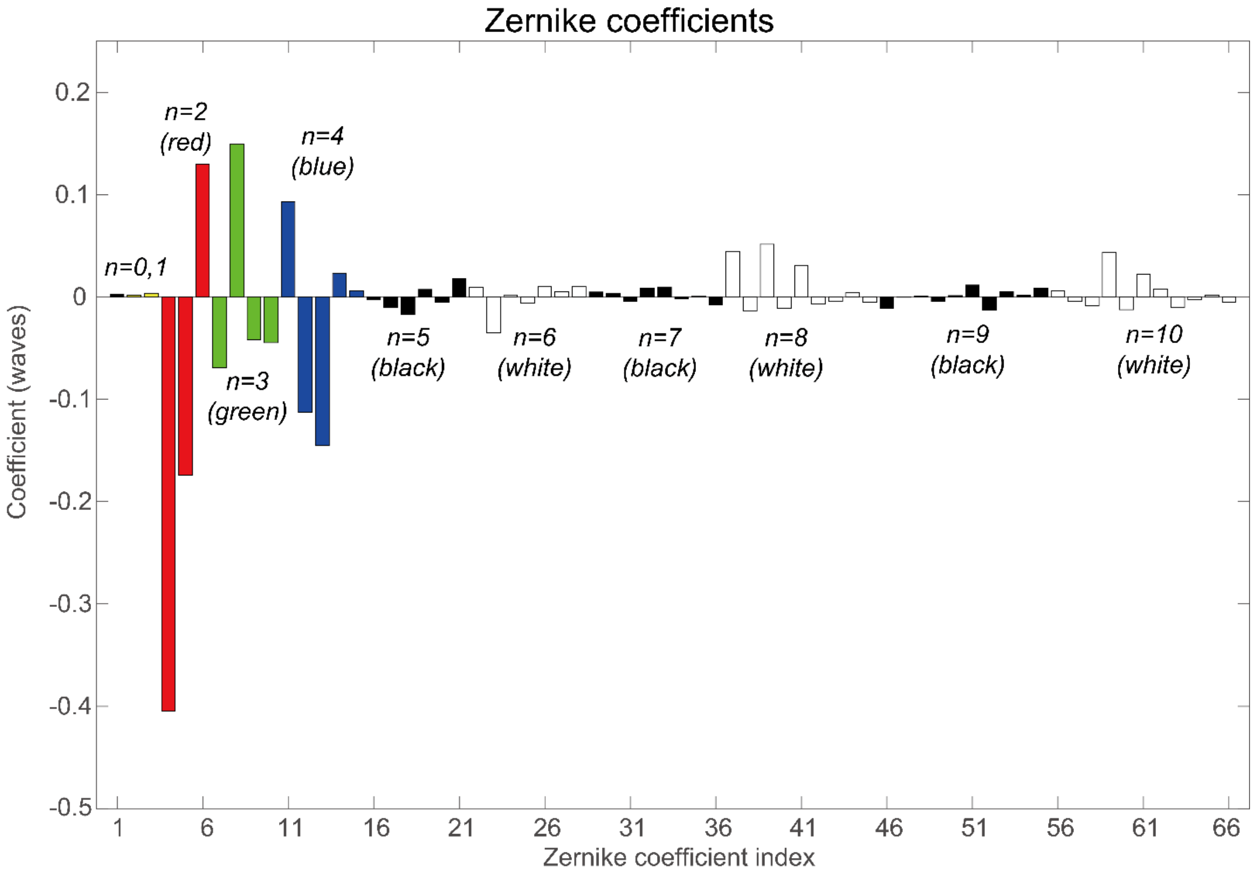

Fig. S11. Fitted coefficients of Zernike polynomials of the measured WAF up to 66th order (10th degree)

The bar plots correspond to the fitted WAF coefficient values in Table S2. The bars are colored by the radial degree (*n*) of Zernike polynomial for visual discrimination. Major contribution to aberration comes from the 2nd (colored red, index no. 4-6) and the 4th (colored blue, index no. 11-15) radial degrees. The large contribution from the horizontal coma (colored green, 3rd degree, index no. 8) is likely from the field-dependent aberration due to the misalignment of the metalens in the interferometry setup.

Index	Degree (n)	Order (m)	Coefficient (waves)	Common name
1	0	0	0.002	Piston
2	1	1	0.002	Horizontal (x) tilt
3		-1	0.003	Vertical (y) tilt
4	2	**2**	**-0.405**	**Vertical astigmatism**
5		**0**	**-0.174**	**Defocus**
6		**-2**	**0.130**	**Oblique astigmatism**
7	3	3	-0.069	Oblique trefoil
8		**1**	**0.149**	**Horizontal coma**
9		-1	-0.041	Vertical coma
10		-3	-0.045	Vertical trefoil
11	4	**4**	**0.093**	**Vertical quadrafoil**
12		**2**	**-0.113**	**Vertical secondary astigmatism**
13		**0**	**-0.145**	**Primary spherical**
14		-2	0.023	Oblique secondary astigmatism
15		-4	0.006	Oblique quadrafoil
16	5	5	-0.003	
17		3	-0.010	
18		1	-0.017	Horizontal secondary coma
19		-1	0.007	Vertical secondary coma
20		-3	-0.005	
21		-5	0.018	
22	6	6	0.009	
23		4	-0.035	
24		2	0.002	
25		0	-0.006	Secondary Spherical
26		-2	0.010	
27		-4	0.005	
28		-6	0.010	
29	7	7	0.005	
30		5	0.004	
31		3	-0.004	
32		1	0.009	
33		-1	0.010	
34	7	-3	-0.002	
35		-5	0.001	
36		-7	-0.008	
37	8	8	**0.044**	
38		6	**-0.013**	
39		4	**0.052**	
40		2	**-0.011**	
41		0	**0.030**	
42		-2	**-0.007**	
43		-4	**-0.005**	
44		-6	**0.004**	
45		-8	**-0.005**	
46	9	9	**-0.011**	
47		7	**0.000**	
48		5	**0.001**	
49		3	**-0.004**	
50		1	**0.001**	
51		-1	**0.012**	
52		-3	**-0.013**	
53		-5	**0.005**	
54		-7	**0.002**	
55		-9	**0.008**	
56	10	10	**0.006**	
57		8	**-0.005**	
58		6	**-0.008**	
59		4	**0.044**	
60		2	**-0.012**	
61		0	**0.022**	
62		-2	**0.008**	
63		-4	**-0.010**	
64		-6	**-0.003**	
65		-8	**0.002**	
66		-10	**-0.005**	

Table. S2. Fitted coefficients of Zernike polynomials of the measured WAF up to 66th order (10th degree)

The Zernike polynomial basis is expressed as:

$$\begin{cases} Z_n^m(\rho,\varphi) = R_n^m(\rho)\cos(m\varphi) & (\text{for } m \geq 0) \\ Z_n^m(\rho,\varphi) = R_n^m(\rho)\sin(m\varphi) & (\text{for } m < 0) \end{cases} \quad \text{(Eq. S2)}$$

Where the radial polynomial $R_n^m(\rho)$ is,

$$R_n^m(\rho) = \sum_{s=0}^{(n-m)/2} (-1)^s \frac{(n-s)!}{s!\left(\frac{n+m}{2}-s\right)!\left(\frac{n-m}{2}-s\right)!}\rho^{n-2s}. \quad \text{(Eq. S3)}$$

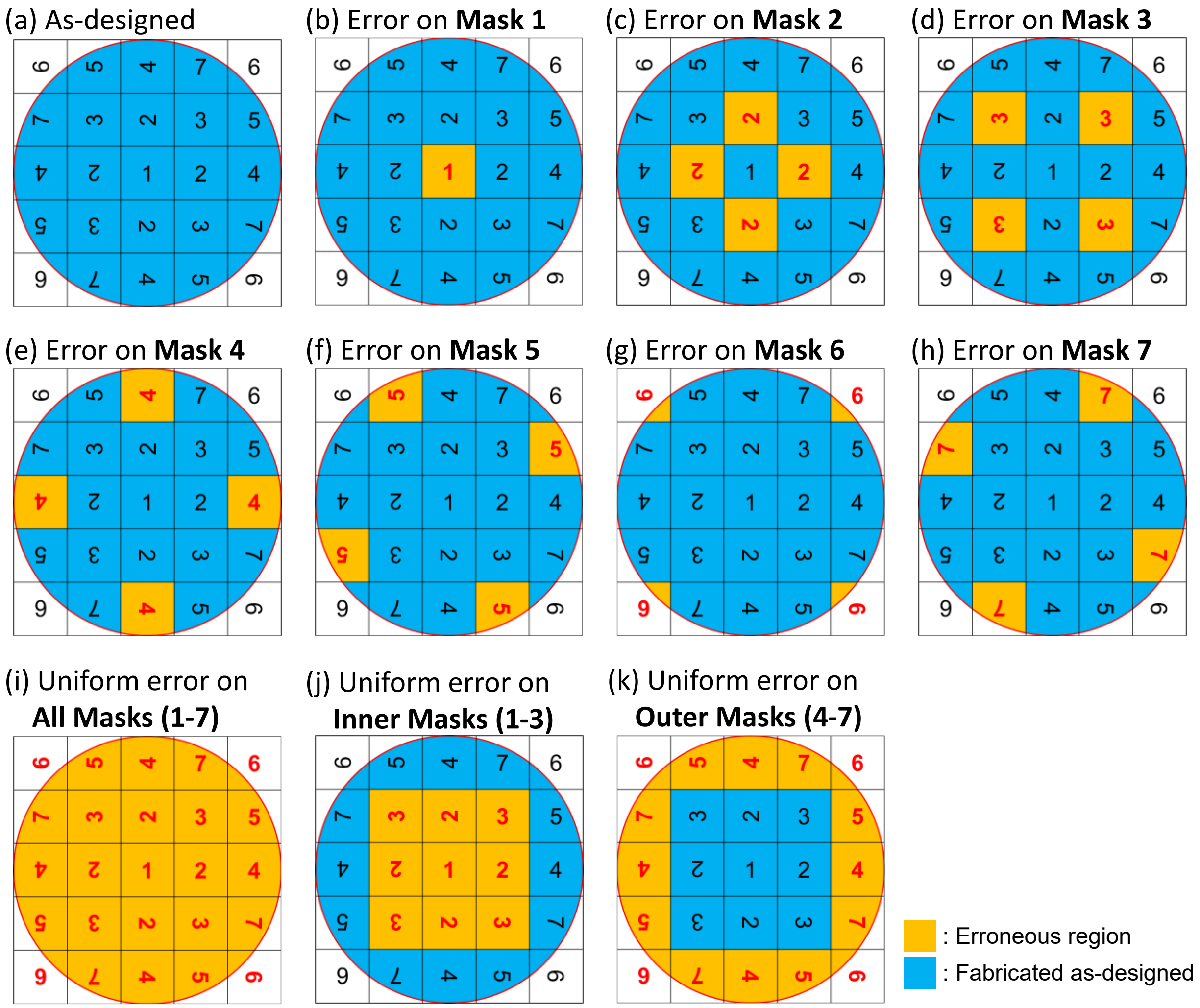

Fig. S12. Schematic of considered fabrication error scenarios.

The considered error scenarios in the simulation-based study are as follows: (1) Small pillars are not resolved (not meeting critical dimension criterion), (2) shift in nanopillar diameters and (3) error in nanopillar height (due to error during etching). (a) Distribution of reticle patterns of an as-designed metalens, having no fabrication error. Reticle (Mask) 1 is placed at the center and Mask 2-7 are distributed at each quadrant rotated by 0°, 90°, 180° and 270°, respectively. (b)-(h) Distribution of an erroneous mask across the metalens, when Mask 1 to Mask 7, respectively, is wrongly fabricated. (i) Distribution of erroneous masks when all 7 are erroneous. Distribution of erroneous masks when (j) Masks 1-3 (inner sections) and (k) Masks 4-7 (outer sections) have error. The dominating fabrication error of the measured metalens in the manuscript corresponds to the case of (k) with error scenario (1).

Non-ideality	Fields affected	Error sources	Focusing efficiency	Strehl ratio
**Smaller pillars missing**	All	Poor reticle quality (Low-resolution reticle writer)	**> 6%** (Min. pillar D < 550 nm)	**~1** (Min. pillar D < 550 nm)
	Inner fields only	Inner reticles: Low-resolution Outer reticles: High-resolution	**> 44%** (Min. pillar D < 550 nm)	**> 0.67** (Min. pillar D < 550 nm)
	Outer fields only	Inner reticles: High-resolution Outer reticles: Low-resolution	**>39%** (Min. pillar D < 550 nm)	**> 0.64** (Min. pillar D < 550 nm)
**Pillar diameter shift**	All	Fabrication error	**> 68%** ($D \pm 50$ nm)	**~1** ($D \pm 50$ nm)
	Individual fields	Reticle quality control	**> 77%** ($D \pm 50$ nm)	**> 0.98** ($D \pm 50$ nm)
	Inner fields only	Inner reticles: Low-resolution Outer reticles: High-resolution	**> 74%** ($D \pm 50$ nm)	**> 0.96** ($D \pm 50$ nm)
	Outer fields only	Inner reticles: High-resolution Outer reticles: Low-resolution	**> 73%** ($D \pm 50$ nm)	**> 0.96** ($D \pm 50$ nm)
**Pillar height shift**	All	Fabrication error (error in etch rate)	**> 48%** ($H \pm 500$ nm)	**~1** ($H \pm 500$ nm)
**Non-uniform illumination**	All	Measurement setup error	N/A	**> 0.99** (Gaussian w > 38 mm)

Table. S3. Summary of simulation results on various fabrication and characterization error scenarios.
High-resolution and low-resolution reticles correspond to industry-grade and in-house fabricated reticles, respectively. The main contributing factor to the aberrated focal spot obtained in experiment is the missing smaller pillars in the outer fields.

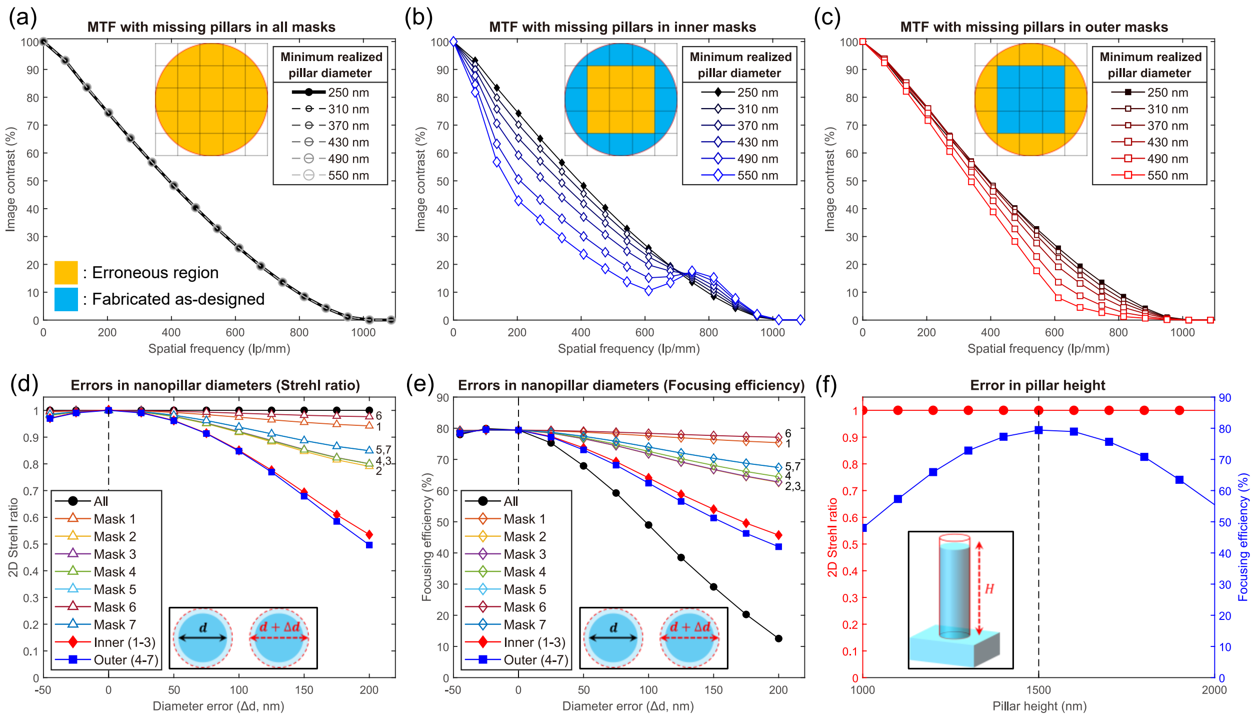

Fig. S13. Effects of fabrication errors on the metalens imaging quality (simulation results). MTF contrast curves with respect to minimum resolved nanopillar diameter in (a) all sections, (b) only the inner sections (reticles 1-3), and only the outer sections (reticles 4-7). All MTF curves are normalized to their zero-frequency values, respectively. The MTF degradation of (c) resembles that of the experimental data provided in the main text, which corroborates the SEM images and the diffracted intensity data. Effect of uniform offset in nanopillar diameters in all sections, individual sections, inner sections, and outer sections, respectively, on (d) Strehl ratio and (e) focusing efficiency, are shown, respectively. The metalens behaves in a diffraction limited manner (Strehl ratio > 0.8) even if the error in diameter is around 100 nm. This is due to nanopillars having a near-linear phase change with respect to their diameter (Fig. S2a), that each section experiences low phase aberration despite the overall diameter error. However, as the diameter offset between sections induce phase offsets between the sections (Fig. S2c), the focusing efficiency suffers due to out-of-phase interference at the focal plane between the sections. (f) shows effect of having overall error in nanopillar height on the Strehl ratio and focusing efficiency of the metalens. As the phase response of nanopillars with respect to height changes almost linearly (Fig. S2d), the Strehl ratio remains near unity even at with ±33% error or ±500 nm. However, when the height of the nanopillars deviates from the designed height of 1500 nm, the relative phase between the nanopillars and the empty area no longer meets the design, leading to a loss in focusing efficiency due to out-of-phase interference. We expect that if we can fill the metalens entirely with nanopillars, such loss of focusing efficiency by height error can be minimized.

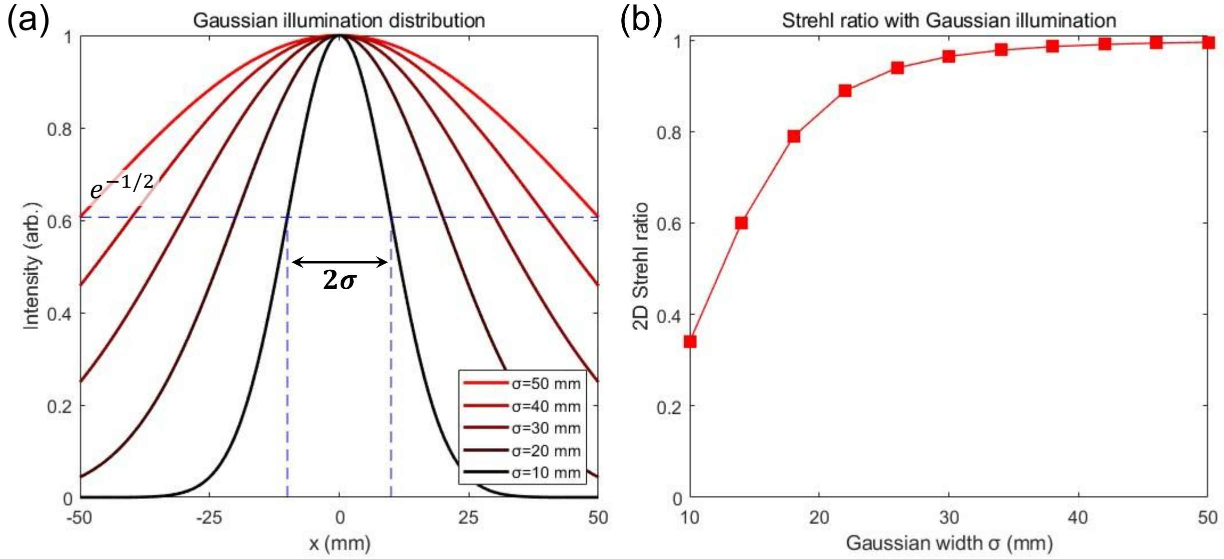

Fig. S14. Effects of non-uniform illumination intensity on the metalens imaging quality (simulation results).

We investigate the effect of non-uniform illumination intensity by modelling the incident intensity profile using a cylindrically symmetric Gaussian intensity profile with a flat phase front. The Gaussian width w is defined so that the radial intensity profile has the form $I(\rho) = I_0 \exp(-\frac{\rho^2}{w^2})$. The Strehl ratio drops below the diffraction limit (0.8) when the Gaussian width of the incident beam is less than 40% of the total aperture size.

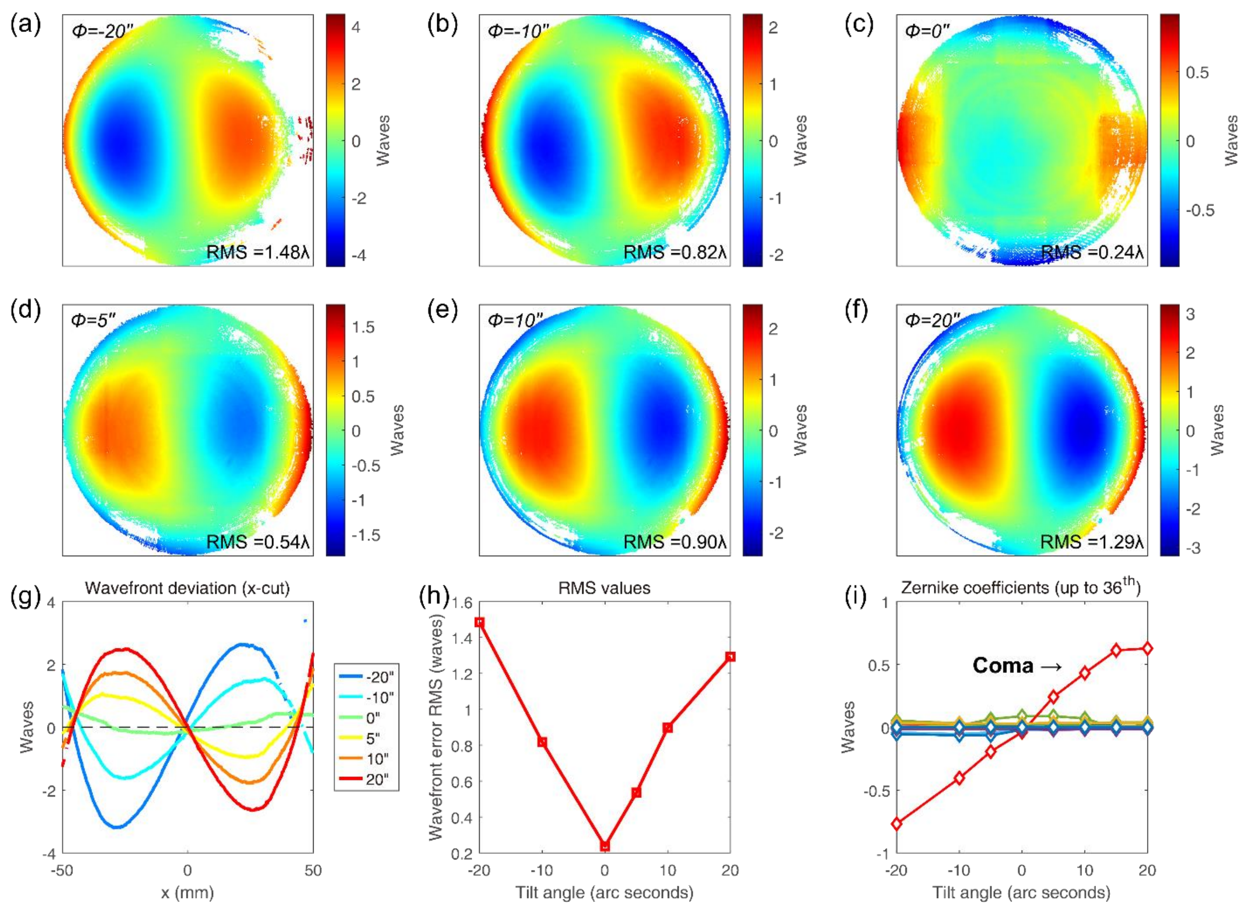

Fig. S15. Measured wavefront error map with respect to various tilted incidence angles. Measured wavefront aberration from null interferometry configuration (Fig. S10) when metalens is tilted from normal incidence. This data represents and confirms the expected field-dependent aberration from a single optical component. Also, this measurement provides alignment tolerance in tilt when the metalens is placed in an imaging system. (a)-(f) Wavefront error of the metalens at various tilt angles ranging from -20” (arc seconds) to +20” after piston, tip, tilt, and power are removed. Due to low diffraction efficiencies at the outer region, interference information from certain points is not resolved. (g) Plots of wavefront error along the x-axis of the metalens at each measured incidence angles, respectively. (h) RMS of the wavefront error with respect to metalens tilt angles. (i) Plots of fitted Zernike polynomial coefficients up to 36th order with respect to the metalens tilt angles. Due to the lack of information at the edge of the metalens, fittings were performed using data from the central 80 mm diameter aperture area. The coma aberration is the dominant wavefront error with respect to lens tilt.

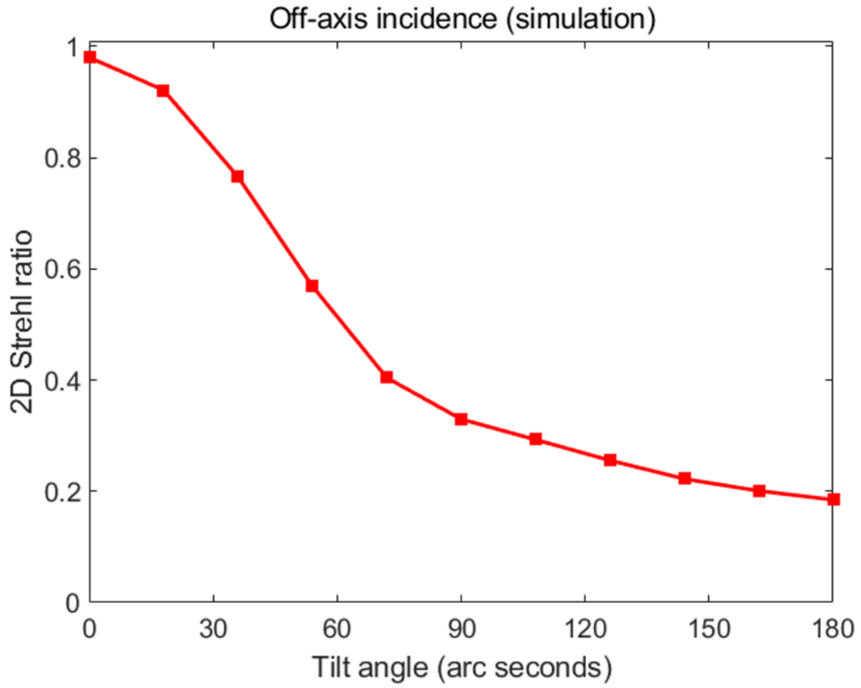

Fig. S16. Simulated Strehl ratio of the 100 mm diameter metalens at various incident angles. For this tilt study, the metalens focal spot is sampled on a 601 × 601 square grid around the tilted focal spot center up to a transverse diameter of 18 Airy disks. This large sampling region is required to capture the spatial patterns associated with strongly aberrated focal spots. The incident tilt angle is defined as the angle of the incident wavefront in air before it hits the first air/glass interface on the back face of the metalens. The Strehl ratio falls below the diffraction limit (~0.8) at a 30" tilt.

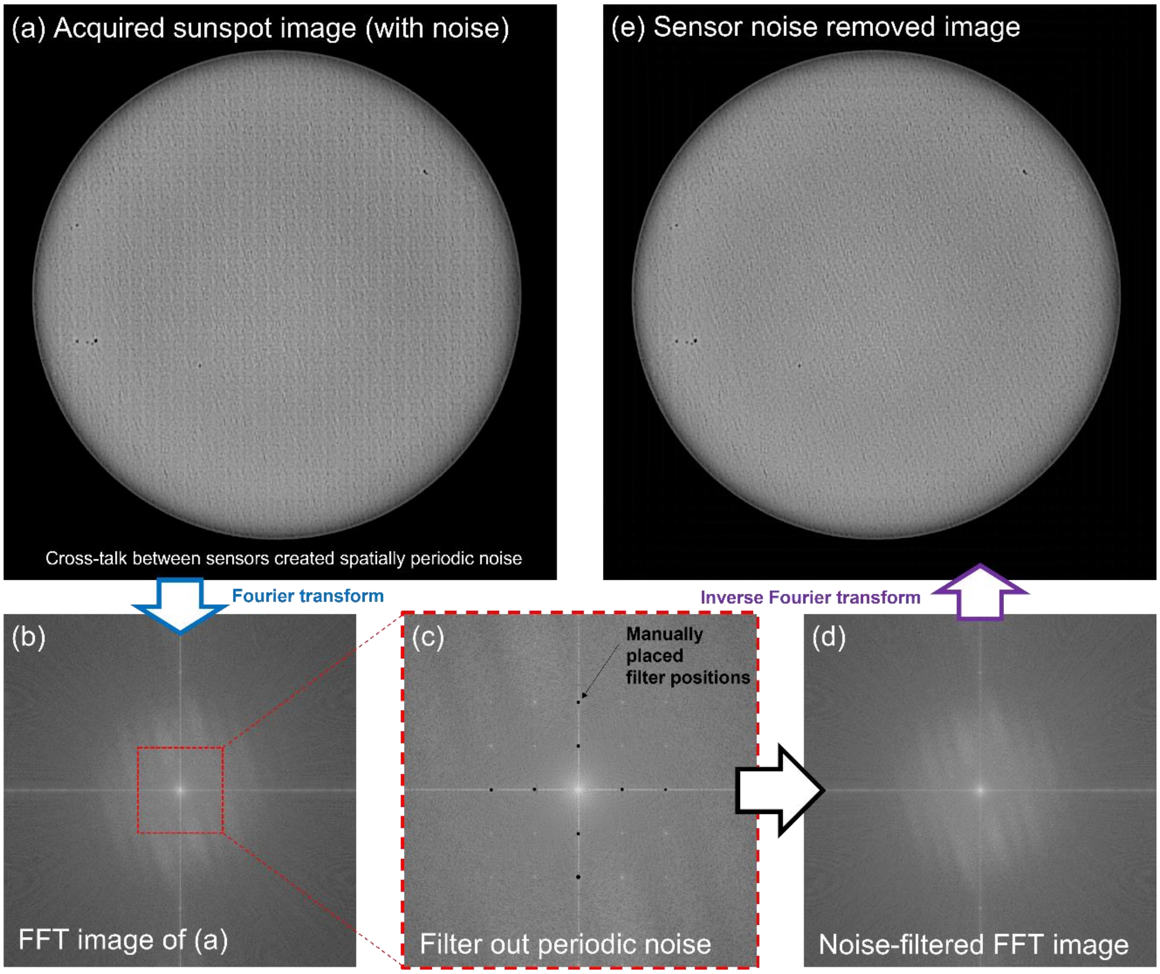

Fig. S17. Sensor-induced noise removal with FFT for Sunspot imaging.

(a) Strong intensity incidence from the Sun caused crosstalk between pixels of the camera sensors due to the on-sensor microlens array. Image taken on Feb. 1, 2023, 12:37 PM, on the rooftop of the Science Center building, Harvard University, Cambridge, Massachusetts. The meta-astroimager was mounted on a ZWO AM5 equatorial mount in sun tracking mode. Two filters, a 1 nm bandwidth $\lambda = 632.8\,nm$ bandpass filter (FL632.8-1, *Thorlabs*) and a neutral density filter of optical density (OD) 3.0, were placed in front of the image sensor. Imaging was performed using a ZWO ASI183mm-pro cooled CMOS imaging sensor (monochrome, 13.2 mm × 8.8 mm sensor, 2.4μm × 2.4μm pixel size) cooled to 10.5℃, with 8 ms exposure time per frame in RAW16 format and with a gain value of 85. Pixel binning was not used. A total of 1000 frames were used to process the image, using Astrosurface software (http://astrosurface.com/ U2 2023-01-25) with following parameters: Algo C quality estimator, 50 pixel tracking shift max, Planet/Disk target, 50% (500 frames) stack, stacking method by mean, global alignment mode, and sharpening using Wavelet-Deconvolution algorithm built in the Astrosurface software. (b) Fast Fourier transform (FFT) image obtained from (a) using ImageJ software (https://imagej.net). (c) Filter positions in *k*-space image to reduce low-order periodic noise. (d) FFT image after the noise filtering is applied. (e) Inverse-FFT image of (d), having some of the pixel noise removed. The sunspot image taken by public sources on the same date can be found online for comparison (https://soho.nascom.nasa.gov/data/synoptic/sunspots_earth/sunspots_512_20230201.jpg).

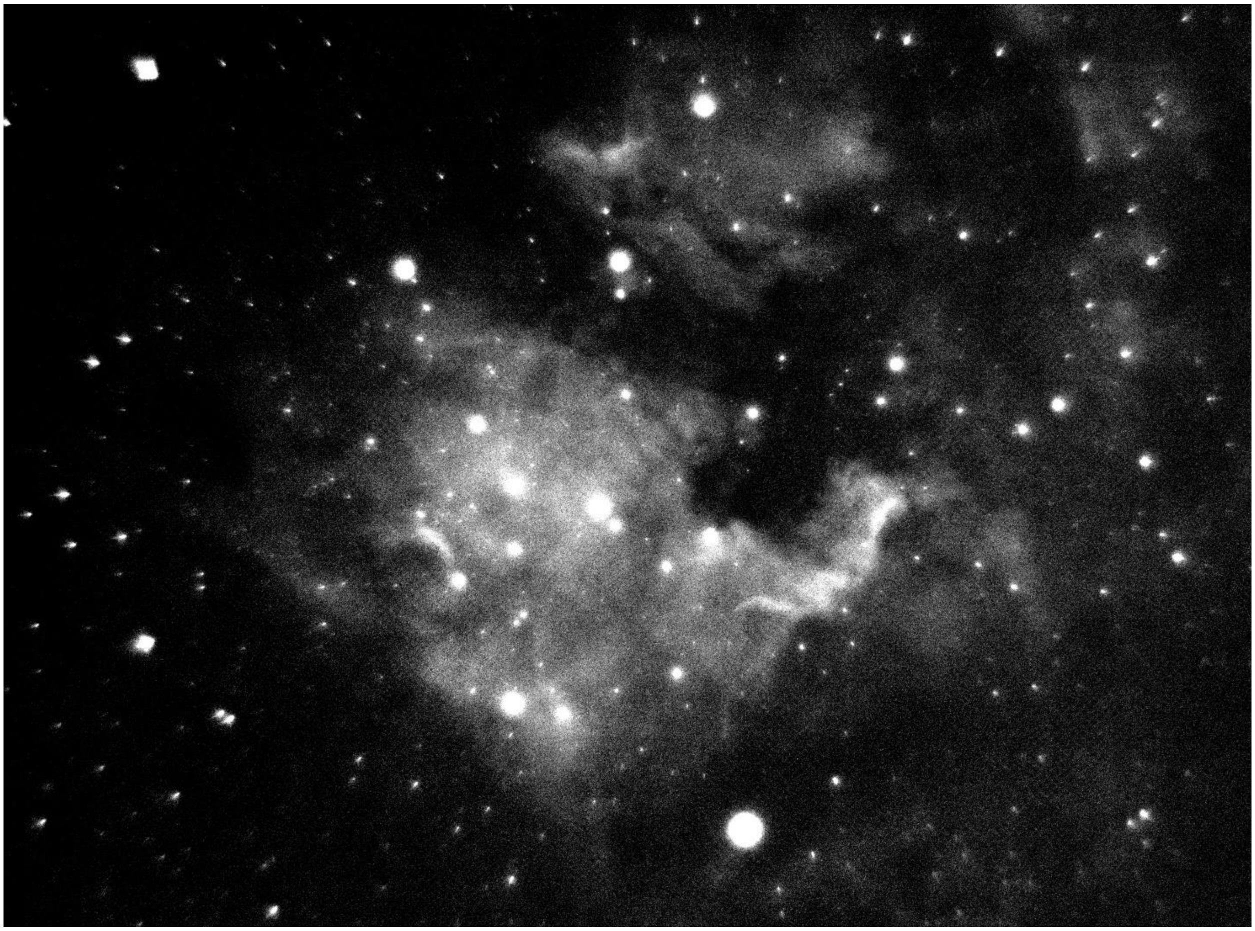

Fig. S18. Acquired high-resolution image of the North America Nebula (NGC7000).

Image of the North America Nebula (NGC7000) acquired from rooftop of the Science Center Building, Harvard University, Cambridge, Massachusetts, USA, on May 13, 2022. The used metalens astro-imager comprises a 100 mm diameter metalens, SVBONY 2-inch, 7 nm bandwidth H-alpha filter, and a ZWO ASI1600mm-pro cooled CMOS mono camera (4656 × 6520 pixels, 3.8 μm × 3.8 μm pixel size), mounted on a Celestron AVX equatorial mount with optical tracking provided from a QHY Mini Guide Scope mounted to a guide-camera (ASI178mm, ZWO). The image sensor was cooled to 0.5 °C, and 11 light frames and 4 dark frames were acquired with 120 second exposure time per frame in RAW12 format with a gain value of 200. Pixel binning was not used. The acquired images are then processed using Deep Sky Stacker (http://deepskystacker.free.fr/) software, with standard mode (Light: average, Dark: median, Alignment: Automatic).

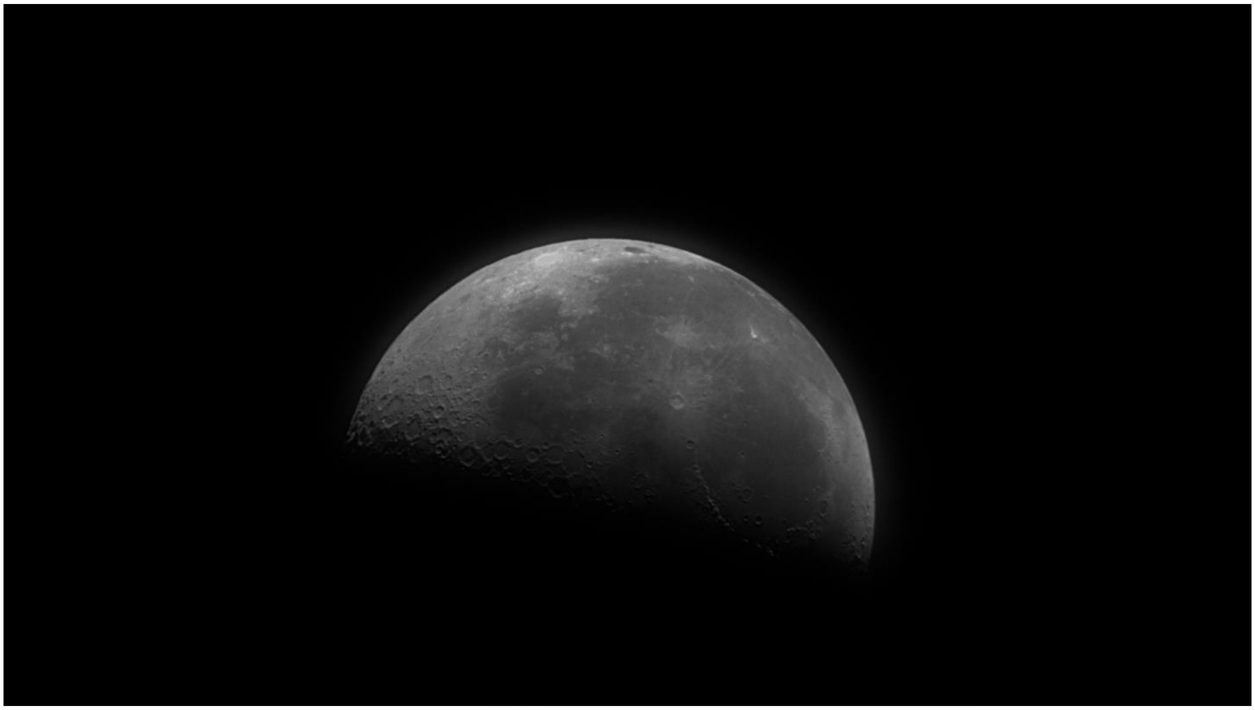

Fig. S19. Acquired high-resolution image of the moon.

Image of the Moon acquired from rooftop of the Science Center Building, Harvard University, Cambridge, Massachusetts, USA, on August 18, 2022. The used metalens astro-imager comprises a 100 mm diameter metalens, a 1 nm bandwidth bandpass filter at $\lambda = 632.8\ nm$ (FL632.8-1, Thorlabs), and a ZWO ASI183mm-pro cooled CMOS mono camera (monochrome, 13.2 mm × 8.8 mm sensor, 2.4 µm × 2.4 µm pixel size) with sensor temperature at -0.5℃, mounted on an iOptron ZEQ25 equatorial mount controlled by ZWQ AsiAir Plus controller. Total of 413 frames were acquired with 442 ms exposure time per frame in RAW8 format and a gain value of 111. Pixel binning was not used. The acquired images are processed using Autostakkert software (https://www.autostakkert.com/, Image stabilization: Planet (COG), Dynamic background, Quality estimator: Local (AP), Frames to stack: 46% (189 frames), Drizzle 1.5x) and wavelet deconvolution was performed with Registax 6 software (https://www.astronomie.be/registax/).

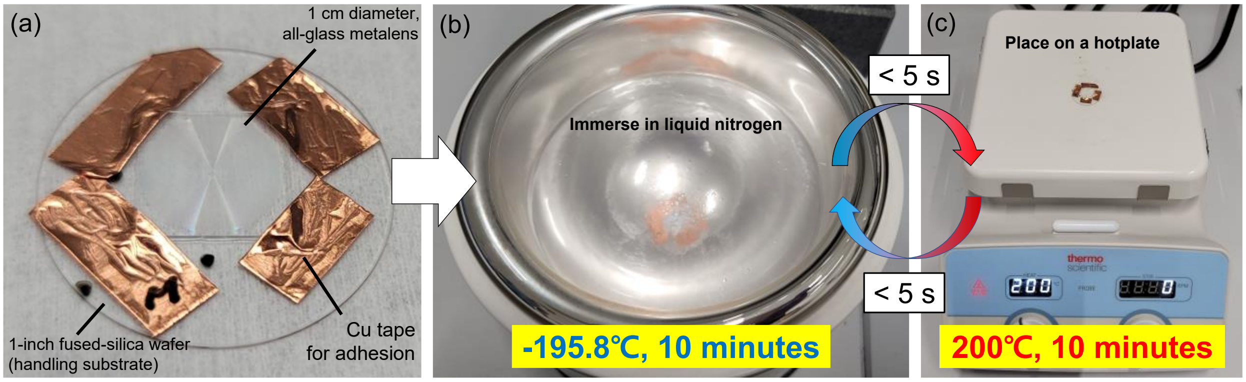

Fig. S20. Extreme environment testing (thermal stress cycles) with 1 cm diameter all-glass metalens.

A 1 cm diameter all-glass metalens (Ref. 1) was used as a proxy to test the glass nanopillar metalens' robustness against extreme temperature shocks that may occur when deployed in an extreme environment such as space. The shock test presented here approximates the United States military specifications (MIL-STD-883F METHOD 1011.9 and 1010.8), in which the sample goes through several cycles of thermal shock between hot and cold thermal reservoirs. (a) A 1 cm diameter metalens, diced into 1.1 × 1.1 cm square, is mounted on a 0.5 mm thick 1-inch diameter fused silica substrate (JGS2) for handling purposes. Copper (Cu) tapes were used to hold the metalens in place. (b) Metalens immersed in liquid nitrogen (-195.8°C) which serves as a cold reservoir. The sample remained in liquid nitrogen bath for 10 minutes to ensure reaching thermal equilibrium. (c) The cooled sample from (b) is then put on a hot plate (200°C) within 5 seconds after being taken out of the cold reservoir. The sample is kept on the hot plate for 10 minutes to ensure reaching thermal equilibrium. The sample is then put back in the liquid nitrogen bath within 5 seconds after it has been taken off the hot reservoir, where the process is repeated for total of 10 cycles, following the MIL-STD-883F METHOD 1011.9.

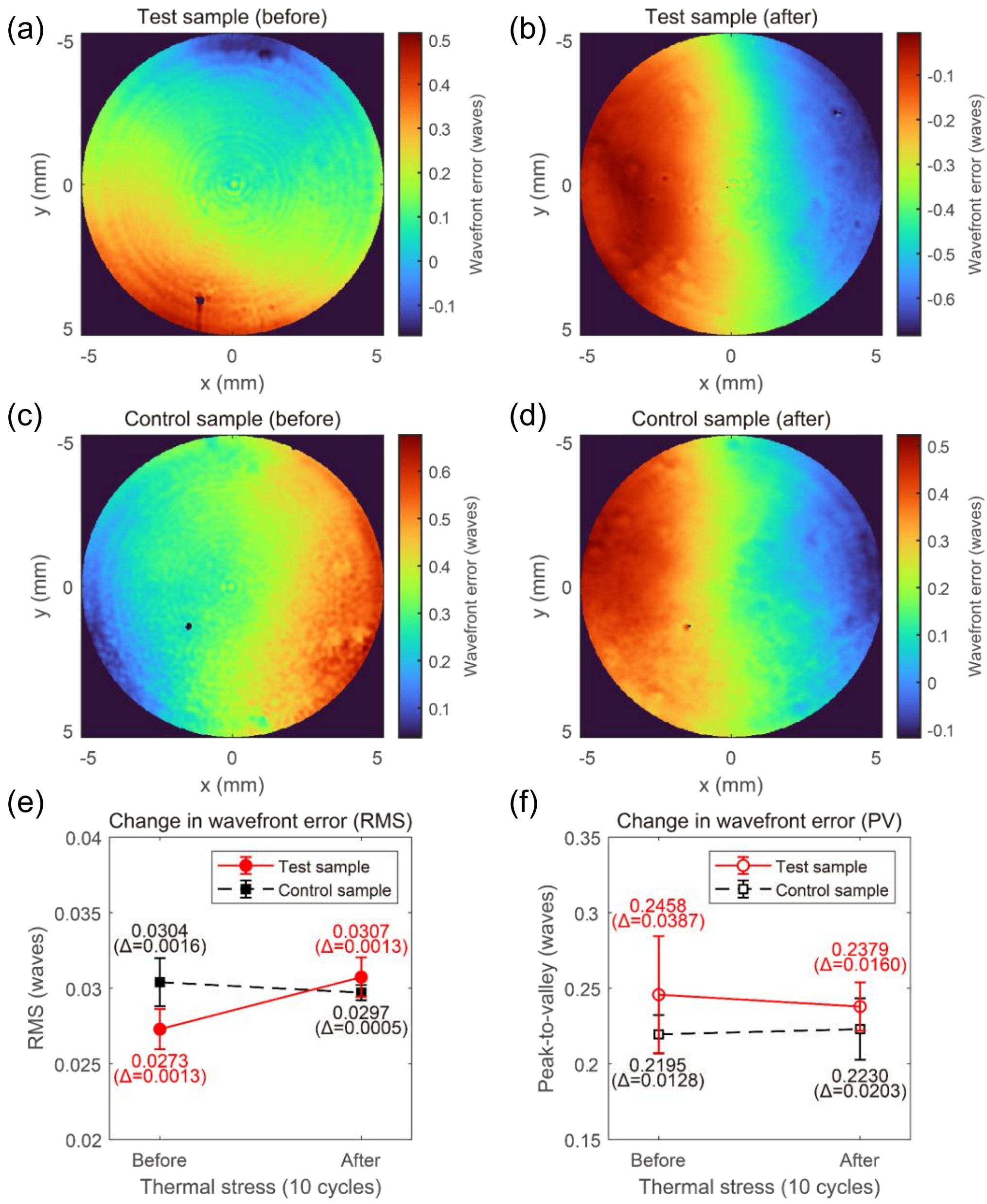

Fig. S21. Result of extreme environment testing (thermal stress) with 1 cm diameter all-glass metalens.

The measured wavefront error of the 1 cm diameter metalens before and after 10 cycles of thermal shock test in Fig. S20 are presented. Wavefront error of the metalens (a) before and (b) after thermal shock cycles. (c) and (d) show wavefront error of another 1 cm diameter metalens (control sample), which did not go through the thermal shock test. (e) Change in measured wavefront error's root-mean-squared (RMS) values before and after thermal shock, compared with that of the control sample. The plotted values are the mean and the standard deviation of 5 independent measurements for each data point, respectively. The RMS value appears to increase slightly, but it is within measurement error. (f) Measured peak-to-valley (PV) value of the wavefront error before and after thermal shock test compared with that of the control sample.

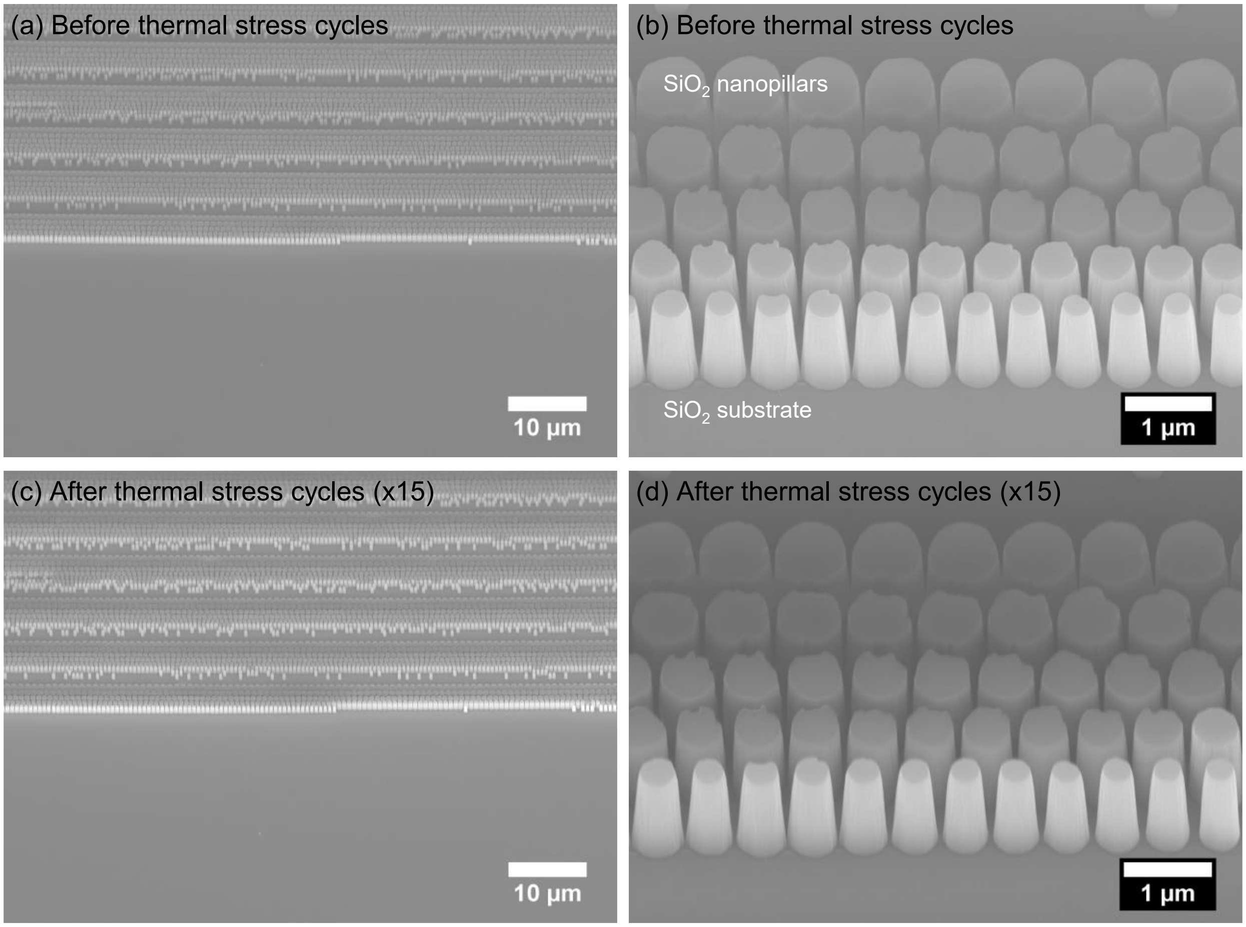

Fig. S22. SEM images of 1 cm diameter all-glass metalens before and after extreme environment testing (thermal stress).

(a) and (b) show the SEM images of the edge of the 1 cm diameter all-glass metalens, before going through the thermal stress cycles. (c) and (d) show the SEM images taken at the same region shown in (a) and (b). No apparent physical damage was observed even after 15 cycles of the thermal shock cycles. Gaps in the rings are where the nanopillars were not fabricated due to photomask resolution limitations. We do not observe evidence of fabricated nanopillars being damaged as they leave a telltale mark at the base when they are broken off from the substrate.

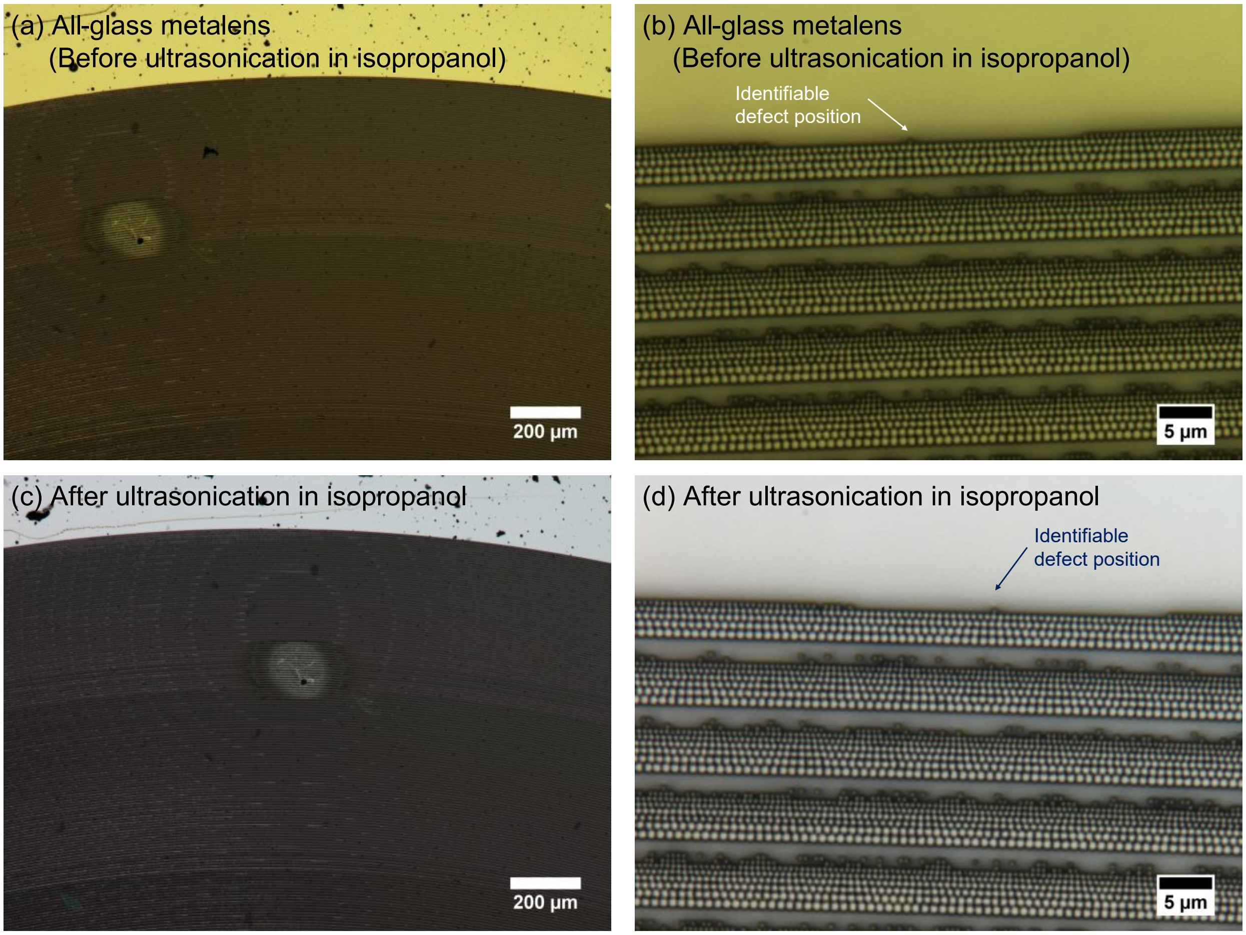

Fig. S23. Optical microscope images of 1 cm diameter all-glass metalens before and after extreme environment testing (vibrational stress).

A 1 cm diameter metalens was immersed in isopropanol and put in an ultrasonication bath (Ultra Clean Equipment, Inc.) for 20 minutes at the highest power setting to see effects of the vibrational stress on the nanopillars. From optical microscope images taken (a), (b) before and (c), (d) after ultrasonication, the nanopillars seem to remain intact even after prolonged exposure to sonic waves. The optical images are taken at a similar location to show that the nanopillars remain intact. Gaps in the rings are where the nanopillars were not fabricated due to photomask resolution limitations. We do not observe evidence of fabricated nanopillars being damaged as they leave a telltale mark at the base when they are broken off from the substrate.

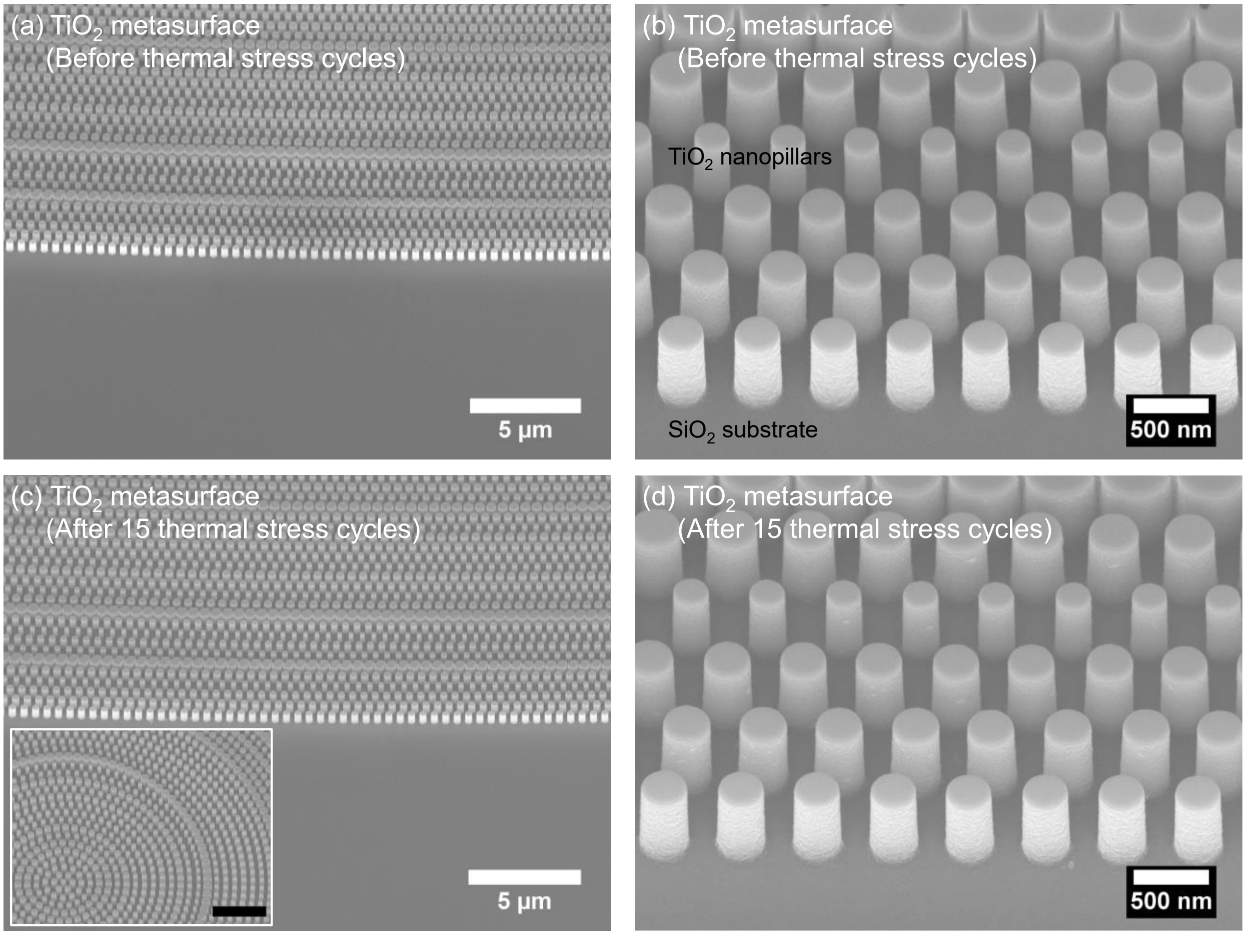

Fig. S24. SEM images of 1 mm diameter TiO_2 metasurface before and after extreme environment testing (thermal stress).

A 1 mm diameter TiO_2 metasurface (700 nm tall, Ref. 45) on a 1-in. diameter fused silica wafer went through the same thermal shock cycles, illustrated in Fig. S20, showing their robustness against rapid temperature fluctuations. The scalebar in the inset of (c) is 3 µm. More in-depth and controlled environmental testing will need to be performed to further confirm the robustness.

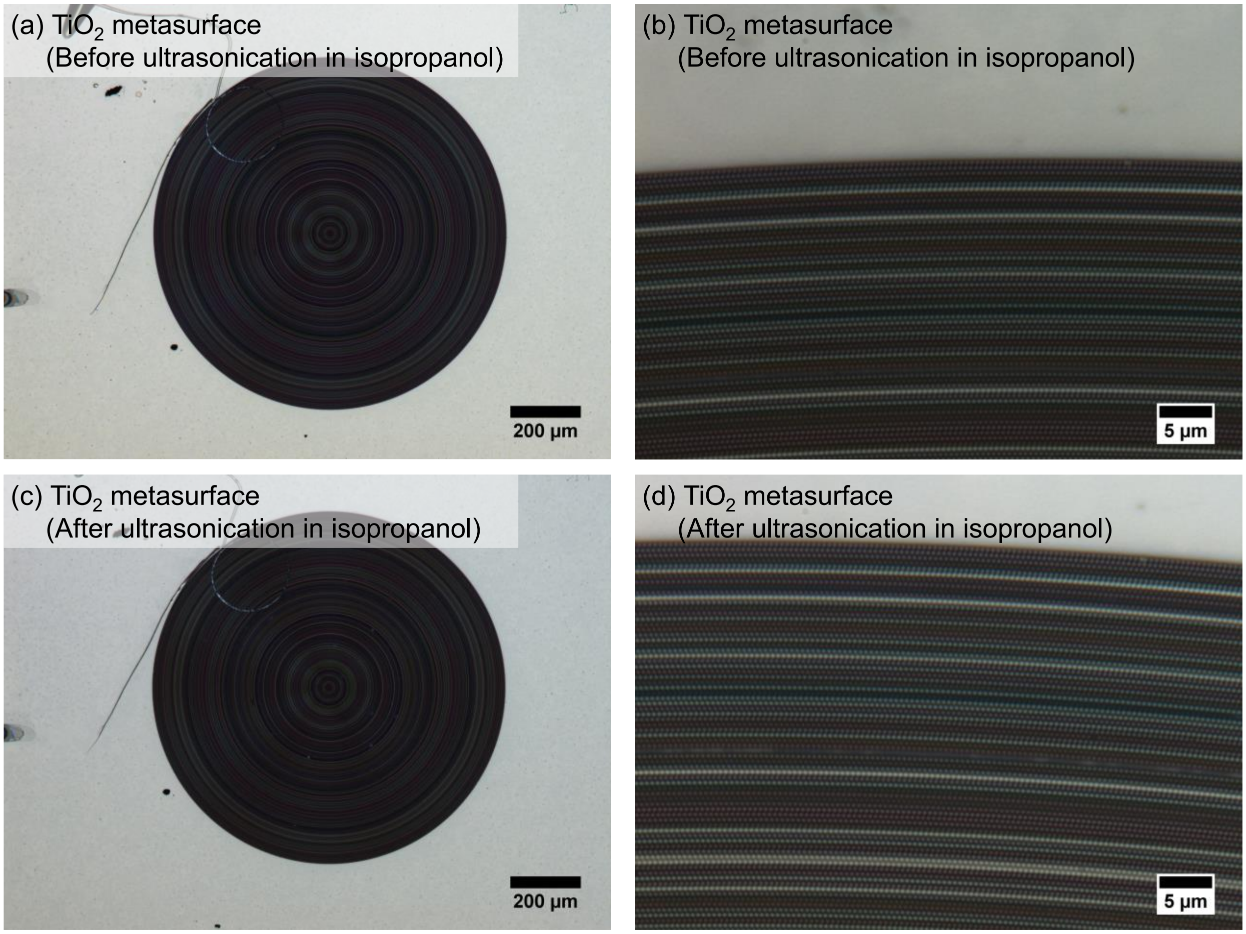

Fig. S25. Optical microscope images of 1 mm diameter TiO_2 metasurface before and after extreme environment testing (vibrational stress).

A 1 mm diameter TiO_2 metasurface (700 nm tall, Ref. 45) fabricated with e-beam lithography on a 1-in. diameter fused silica wafer went through the same 20 minute ultrasonication as illustrated in Fig. S23, which show their robustness against sonic waves. More in-depth and controlled environmental testing will need to be performed to further confirm their robustness.

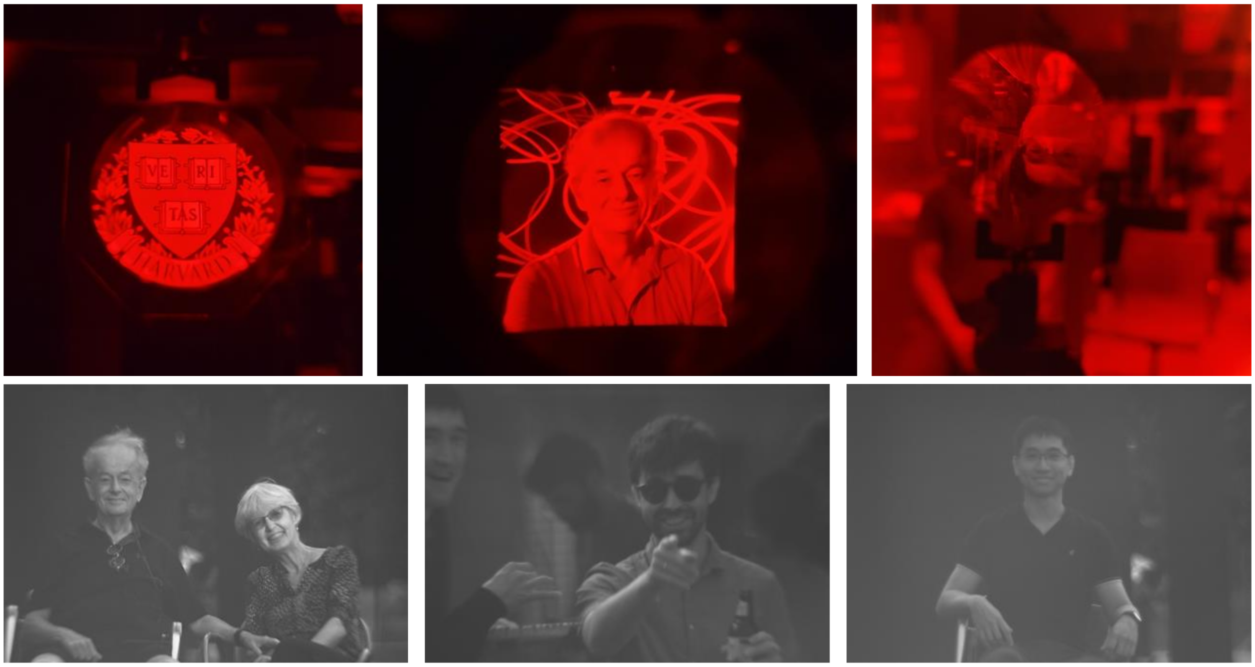

Fig. S26. Other acquired images with the metalens (top row) and the meta-astrophotography apparatus (bottom row).

(Top row) Images through the 100 mm metalens taken with a smartphone camera (Galaxy Note 20 Ultra, *Samsung*) with a 10 nm bandwidth 633 nm bandpass filter. Left two images are projected from a digital monitor (Galaxy Tab S7 Plus, *Samsung*). Image on the right shows an inverted image of the person (Soon Wei Daniel Lim) sitting on the other side of the lens. (Bottom row) Images taken with the meta-astrophotography apparatus, with adjusted focus using the helical focuser. Left photo is Federico and Paola Capasso. Middle photo is Arman Amirzhan (left) and Vincent Ginis (right), and right photo is Soon Wei Daniel Lim. All images are taken by Joon-Suh Park.

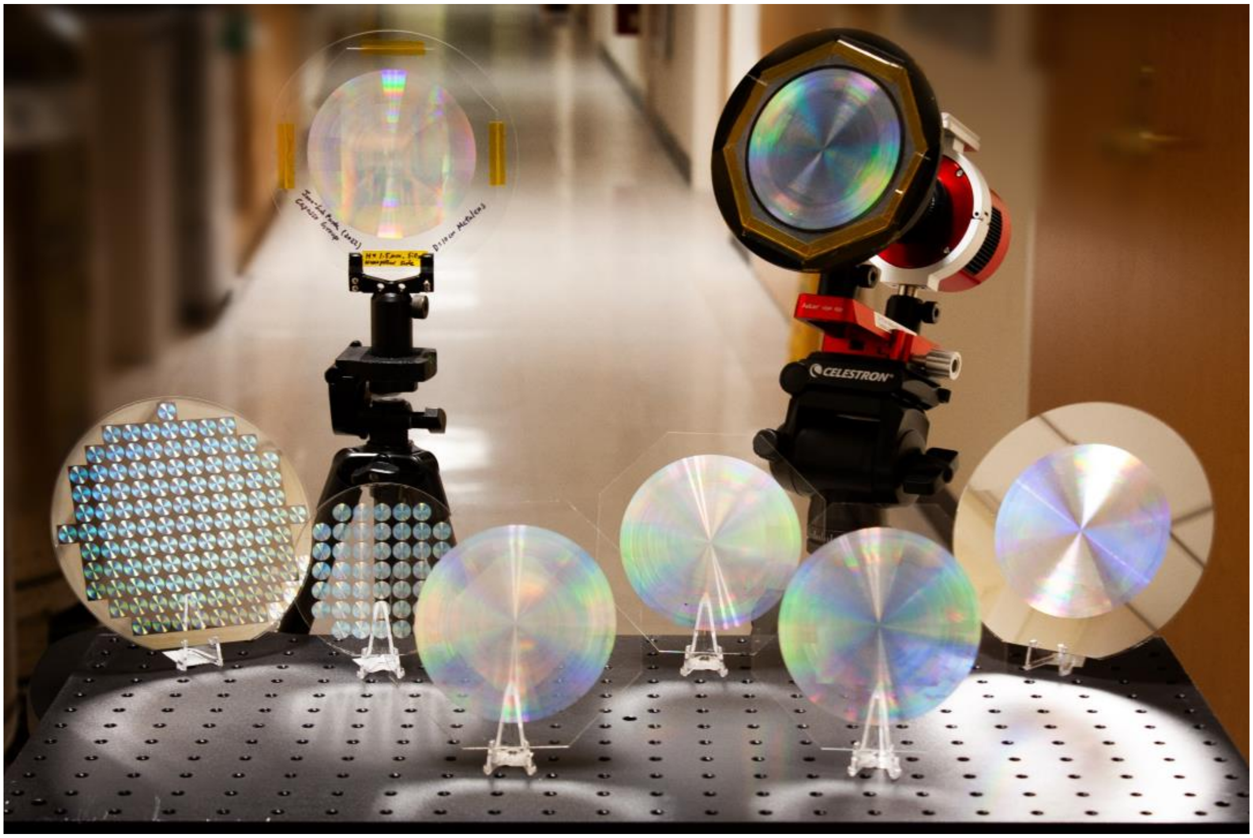

Fig. S27. Mass-producible, DUV fabricated all-glass metalenses.

Photograph of the fabricated 10 mm diameter[1], and 100 mm diameter all-glass metalenses. Total of ten 100 mm metalenses were fabricated, and five of the fabricated lenses are shown in the picture. The wafer on the bottom right shows the 100 mm diameter metalens photoresist pattern on an aluminum coated 150 mm fused silica wafer. The metalens mounted on the meta-astrophotography apparatus is on the top right. The 150 mm and 100 mm diameter wafers on the bottom left corner of the photograph are 10 mm diameter all-glass metalenses[1].

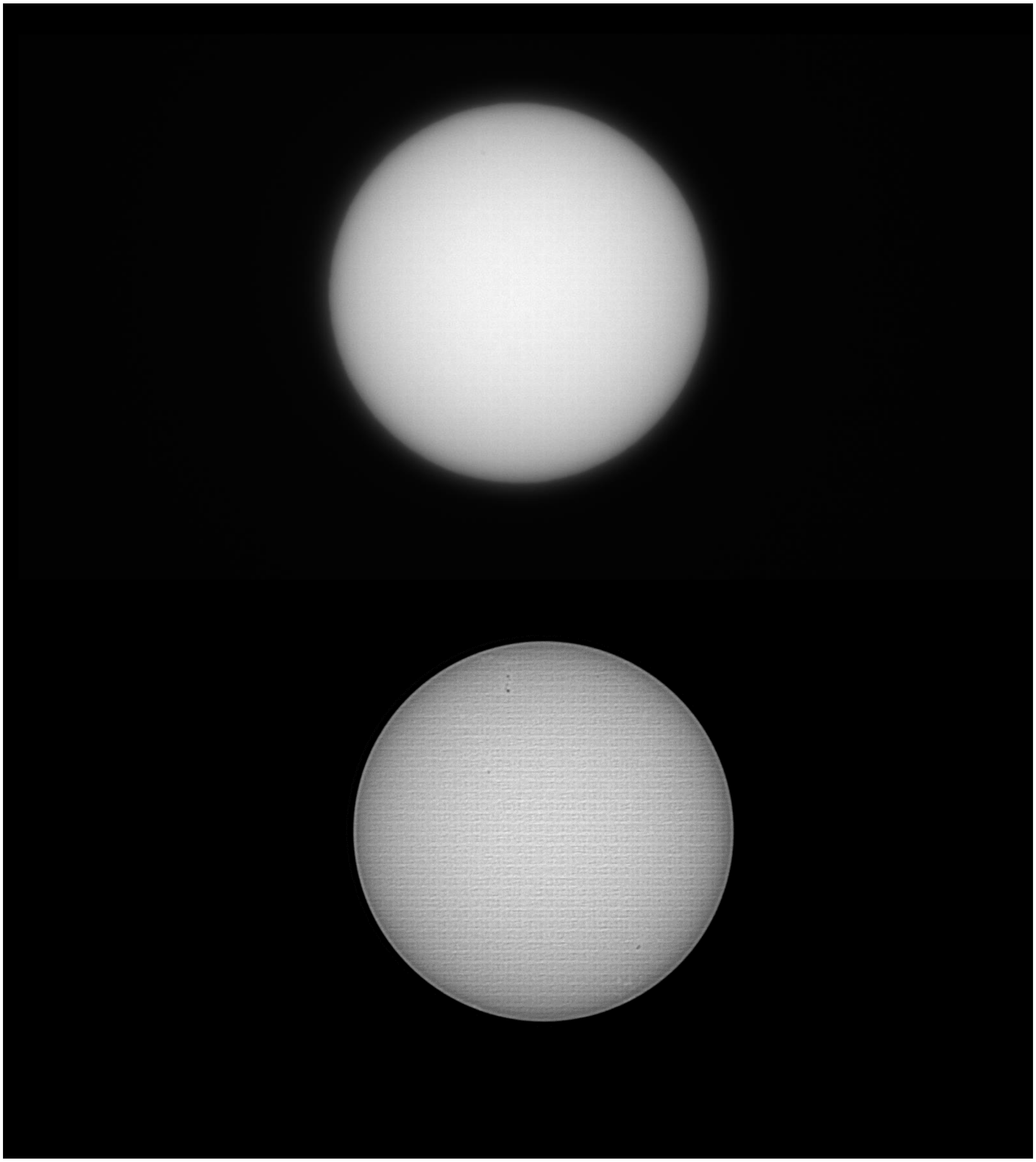

Fig. S28. A raw image frame extracted from the video recording of the Sun prior to image stacking and processing, and a raw stacked image from the video before processing.

(Top) A raw image frame extracted from the video recording of the Sun prior to image processing. The final processed image is a 90° rotated image of the raw image for orientation purposes. Details of the video recording conditions can be found in Fig. S17. (Bottom) A raw form of stacked image of the Sun comprising 1000 frames from the acquired video. The full raw video recording and a processed image can be found at: http://doi.org/10.6084/m9.figshare.24531058.

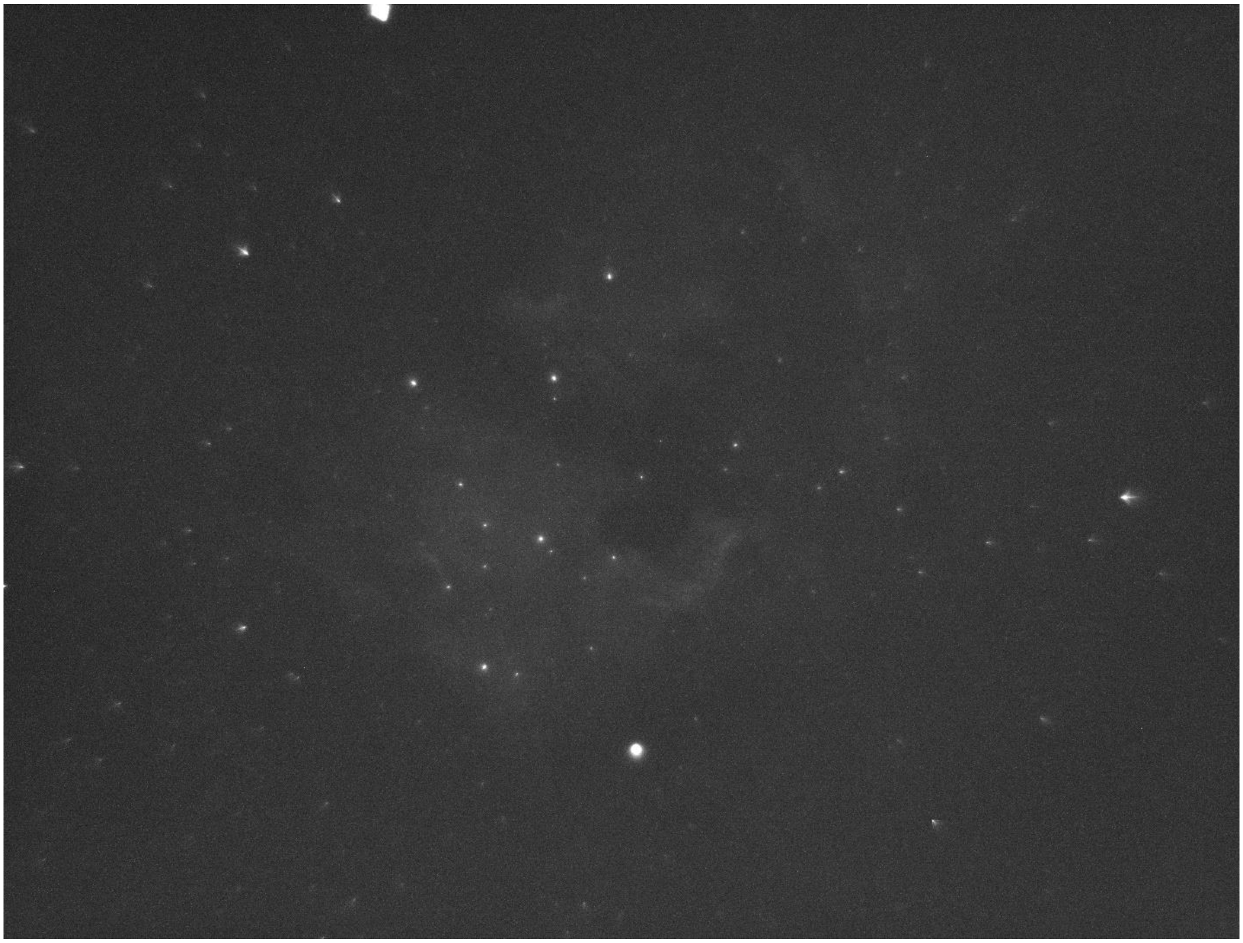

Fig. S29. A raw image of the North America Nebula before noise processing.

Total of 11 exposed images and 4 “dark” images (for pixel dark noise correction, taken with the aperture closed) were used to stack and obtain the figure in Fig. 5 (c). Image acquisition method is detailed in Fig. S18. The full set of the raw images and a processed image can be found at: http://doi.org/10.6084/m9.figshare.24531058.

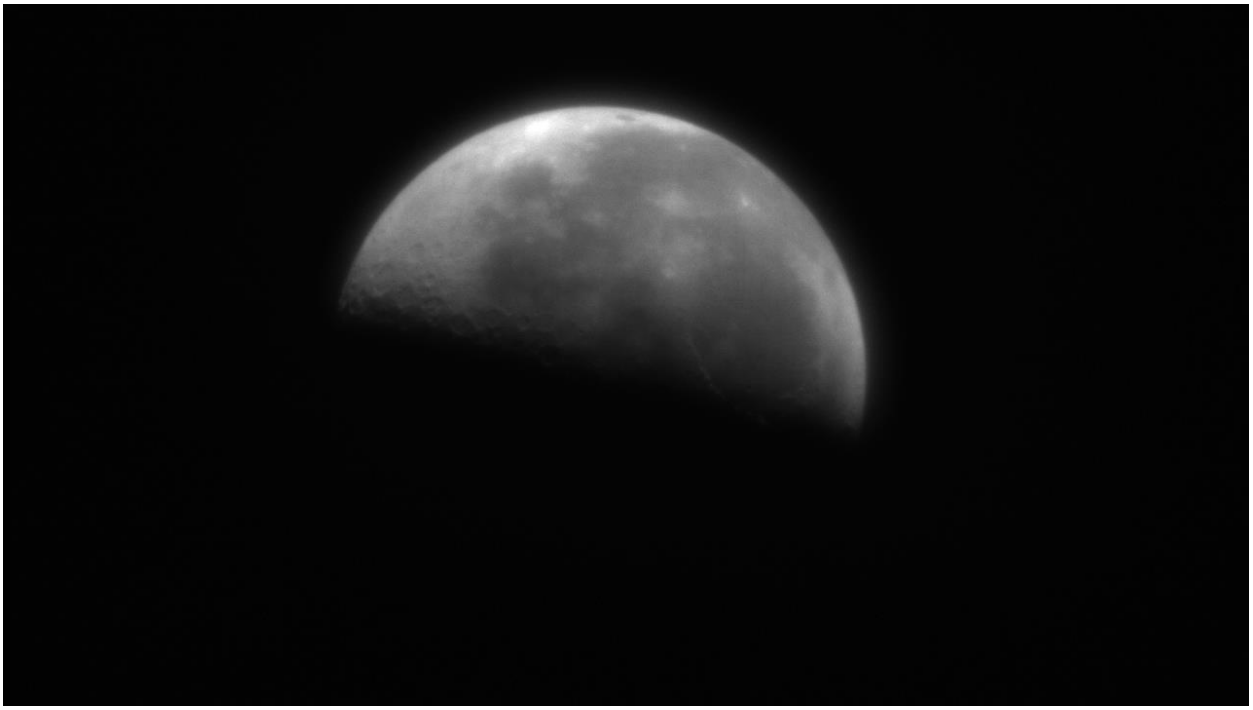

Fig. S30. A raw image frame extracted from the video recording of the Moon prior to image stacking and processing.

A raw image frame extracted from the video recording of the Moon prior to image stacking and processing. Details of the video acquisition conditions can be found in Fig. S19. The full raw video and a processed image can be found at: http://doi.org/10.6084/m9.figshare.24531058.

References

(1) Park, J.-S.; Zhang, S.; She, A.; Chen, W. T.; Lin, P.; Yousef, K. M. A.; Cheng, J.-X.; Capasso, F. All-Glass, Large Metalens at Visible Wavelength Using Deep-Ultraviolet Projection Lithography. *Nano Lett.* **2019**, *19* (12), 8673–8682. https://doi.org/10.1021/acs.nanolett.9b03333.

(2) Aieta, F.; Genevet, P.; Kats, M. A.; Yu, N.; Blanchard, R.; Gaburro, Z.; Capasso, F. Aberration-Free Ultrathin Flat Lenses and Axicons at Telecom Wavelengths Based on Plasmonic Metasurfaces. *Nano Lett.* **2012**, *12* (9), 4932–4936. https://doi.org/10.1021/nl302516v.

(3) Smith, W. *Modern Lens Design*, 2nd edition.; McGraw Hill: New York, 2004.

(4) Maréchal, A. Mechanical Integrator for Studying the Distribution of Light in the Optical Image. *JOSA* **1947**, *37* (5), 403_1-404. https://doi.org/10.1364/JOSA.37.0403_1.

(5) Pestourie, R.; Pérez-Arancibia, C.; Lin, Z.; Shin, W.; Capasso, F.; Johnson, S. G. Inverse Design of Large-Area Metasurfaces. *Opt. Express* **2018**, *26* (26), 33732–33747. https://doi.org/10.1364/OE.26.033732.

(6) Hugonin, J. P.; Lalanne, P. RETICOLO Software for Grating Analysis. arXiv January 8, 2023. https://doi.org/10.48550/arXiv.2101.00901.

(7) Malitson, I. H. Interspecimen Comparison of the Refractive Index of Fused Silica. *JOSA* **1965**, *55* (10), 1205–1209. https://doi.org/10.1364/JOSA.55.001205.

(8) Marathay, A. S.; McCalmont, J. F. Vector Diffraction Theory for Electromagnetic Waves. *JOSA A* **2001**, *18* (10), 2585–2593. https://doi.org/10.1364/JOSAA.18.002585.

(9) Chen, F. T.; Craighead, H. G. Diffractive Lens Fabricated with Mostly Zeroth-Order Gratings. *Opt. Lett.* **1996**, *21* (3), 177–179. https://doi.org/10.1364/OL.21.000177.

(10) Lalanne, P.; Astilean, S.; Chavel, P.; Cambril, E.; Launois, H. Design and Fabrication of Blazed Binary Diffractive Elements with Sampling Periods Smaller than the Structural Cutoff. *JOSA A* **1999**, *16* (5), 1143–1156. https://doi.org/10.1364/JOSAA.16.001143.

(11) Ni, X.; Ishii, S.; Kildishev, A. V.; Shalaev, V. M. Ultra-Thin, Planar, Babinet-Inverted Plasmonic Metalenses. *Light Sci. Appl.* **2013**, *2* (4), e72–e72. https://doi.org/10.1038/lsa.2013.28.

(12) Ding, X.; Monticone, F.; Zhang, K.; Zhang, L.; Gao, D.; Burokur, S. N.; de Lustrac, A.; Wu, Q.; Qiu, C.-W.; Alù, A. Ultrathin Pancharatnam–Berry Metasurface with Maximal Cross-Polarization Efficiency. *Adv. Mater.* **2015**, *27* (7), 1195–1200. https://doi.org/10.1002/adma.201405047.

(13) Khorasaninejad, M.; Chen, W. T.; Devlin, R. C.; Oh, J.; Zhu, A. Y.; Capasso, F. Metalenses at Visible Wavelengths: Diffraction-Limited Focusing and Subwavelength Resolution Imaging. *Science* **2016**, *352* (6290), 1190–1194. https://doi.org/10.1126/science.aaf6644.

(14) Zuo, H.; Choi, D.-Y.; Gai, X.; Ma, P.; Xu, L.; Neshev, D. N.; Zhang, B.; Luther-Davies, B. High-Efficiency All-Dielectric Metalenses for Mid-Infrared Imaging. *Adv. Opt. Mater.* **2017**, *5* (23), 1700585. https://doi.org/10.1002/adom.201700585.

(15) Jia, D.; Tian, Y.; Ma, W.; Gong, X.; Yu, J.; Zhao, G.; Yu, X. Transmissive Terahertz Metalens with Full Phase Control Based on a Dielectric Metasurface. *Opt. Lett.* **2017**, *42* (21), 4494–4497. https://doi.org/10.1364/OL.42.004494.

(16) She, A.; Zhang, S.; Shian, S.; Clarke, D. R.; Capasso, F. Large Area Metalenses: Design, Characterization, and Mass Manufacturing. *Opt. Express* **2018**, *26* (2), 1573–1585. https://doi.org/10.1364/OE.26.001573.

(17) Paniagua-Domínguez, R.; Yu, Y. F.; Khaidarov, E.; Choi, S.; Leong, V.; Bakker, R. M.; Liang, X.; Fu, Y. H.; Valuckas, V.; Krivitsky, L. A.; Kuznetsov, A. I. A Metalens with a Near-Unity Numerical Aperture. *Nano Lett.* **2018**, *18* (3), 2124–2132. https://doi.org/10.1021/acs.nanolett.8b00368.

(18) She, A.; Zhang, S.; Shian, S.; Clarke, D. R.; Capasso, F. Adaptive Metalenses with Simultaneous Electrical Control of Focal Length, Astigmatism, and Shift. *Sci. Adv.* **2018**, *4* (2), eaap9957. https://doi.org/10.1126/sciadv.aap9957.

(19) Shi, Z.; Khorasaninejad, M.; Huang, Y.-W.; Roques-Carmes, C.; Zhu, A. Y.; Chen, W. T.; Sanjeev, V.; Ding, Z.-W.; Tamagnone, M.; Chaudhary, K.; Devlin, R. C.; Qiu, C.-W.; Capasso, F. Single-

Layer Metasurface with Controllable Multiwavelength Functions. *Nano Lett.* **2018**, *18* (4), 2420–2427. https://doi.org/10.1021/acs.nanolett.7b05458.
(20) Zhang, L.; Ding, J.; Zheng, H.; An, S.; Lin, H.; Zheng, B.; Du, Q.; Yin, G.; Michon, J.; Zhang, Y.; Fang, Z.; Shalaginov, M. Y.; Deng, L.; Gu, T.; Zhang, H.; Hu, J. Ultra-Thin High-Efficiency Mid-Infrared Transmissive Huygens Meta-Optics. *Nat. Commun.* **2018**, *9* (1), 1481. https://doi.org/10.1038/s41467-018-03831-7.
(21) Fan, Z.-B.; Shao, Z.-K.; Xie, M.-Y.; Pang, X.-N.; Ruan, W.-S.; Zhao, F.-L.; Chen, Y.-J.; Yu, S.-Y.; Dong, J.-W. Silicon Nitride Metalenses for Close-to-One Numerical Aperture and Wide-Angle Visible Imaging. *Phys. Rev. Appl.* **2018**, *10* (1), 014005. https://doi.org/10.1103/PhysRevApplied.10.014005.
(22) Lee, G.-Y.; Hong, J.-Y.; Hwang, S.; Moon, S.; Kang, H.; Jeon, S.; Kim, H.; Jeong, J.-H.; Lee, B. Metasurface Eyepiece for Augmented Reality. *Nat. Commun.* **2018**, *9* (1), 4562. https://doi.org/10.1038/s41467-018-07011-5.
(23) Wang, A.; Chen, Z.; Dan, Y. Planar Metalenses in the Mid-Infrared. *AIP Adv.* **2019**, *9* (8), 085327. https://doi.org/10.1063/1.5124074.
(24) Miragliotta, J. A.; Shrekenhamer, D. B.; Strikwerda, A. C.; Zgrabik, C. J.; Currano, L. J.; Bonnema, G. T. Multifunctional Infrared Metasurfaces for Polarization Analysis. In *Metamaterials, Metadevices, and Metasystems 2019 (eds Engheta, N., Noginov, M. A., Zheludev, N. I.)*; SPIE, 2019; Vol. 11080, pp 35–42. https://doi.org/10.1117/12.2528854.
(25) Guo, Q.; Shi, Z.; Huang, Y.-W.; Alexander, E.; Qiu, C.-W.; Capasso, F.; Zickler, T. Compact Single-Shot Metalens Depth Sensors Inspired by Eyes of Jumping Spiders. *Proc. Natl. Acad. Sci.* **2019**, *116* (46), 22959–22965. https://doi.org/10.1073/pnas.1912154116.
(26) Hu, T.; Zhong, Q.; Li, N.; Dong, Y.; Xu, Z.; Fu, Y. H.; Li, D.; Bliznetsov, V.; Zhou, Y.; Lai, K. H.; Lin, Q.; Zhu, S.; Singh, N. CMOS-Compatible a-Si Metalenses on a 12-Inch Glass Wafer for Fingerprint Imaging. *Nanophotonics* **2020**, *9* (4), 823–830. https://doi.org/10.1515/nanoph-2019-0470.
(27) Andrén, D.; Martínez-Llinàs, J.; Tassin, P.; Käll, M.; Verre, R. Large-Scale Metasurfaces Made by an Exposed Resist. *ACS Photonics* **2020**, *7* (4), 885–892. https://doi.org/10.1021/acsphotonics.9b01809.
(28) Shen, Z.; Zhou, S.; Li, X.; Ge, S.; Chen, P.; Hu, W.; Lu, Y. Liquid Crystal Integrated Metalens with Tunable Chromatic Aberration. *Adv. Photonics* **2020**, *2* (3), 036002. https://doi.org/10.1117/1.AP.2.3.036002.
(29) Shalaginov, M. Y.; An, S.; Yang, F.; Su, P.; Lyzwa, D.; Agarwal, A. M.; Zhang, H.; Hu, J.; Gu, T. Single-Element Diffraction-Limited Fisheye Metalens. *Nano Lett.* **2020**, *20* (10), 7429–7437. https://doi.org/10.1021/acs.nanolett.0c02783.
(30) Li, Z.; Lin, P.; Huang, Y.-W.; Park, J.-S.; Chen, W. T.; Shi, Z.; Qiu, C.-W.; Cheng, J.-X.; Capasso, F. Meta-Optics Achieves RGB-Achromatic Focusing for Virtual Reality. *Sci. Adv.* **2021**, *7* (5), eabe4458. https://doi.org/10.1126/sciadv.abe4458.
(31) Shalaginov, M. Y.; An, S.; Zhang, Y.; Yang, F.; Su, P.; Liberman, V.; Chou, J. B.; Roberts, C. M.; Kang, M.; Rios, C.; Du, Q.; Fowler, C.; Agarwal, A.; Richardson, K. A.; Rivero-Baleine, C.; Zhang, H.; Hu, J.; Gu, T. Reconfigurable All-Dielectric Metalens with Diffraction-Limited Performance. *Nat. Commun.* **2021**, *12* (1), 1225. https://doi.org/10.1038/s41467-021-21440-9.
(32) Baracu, A. M.; Dirdal, C. A.; Avram, A. M.; Dinescu, A.; Muller, R.; Jensen, G. U.; Thrane, P. C. V.; Angelskår, H. Metasurface Fabrication by Cryogenic and Bosch Deep Reactive Ion Etching. *Micromachines* **2021**, *12* (5), 501. https://doi.org/10.3390/mi12050501.
(33) Einck, V. J.; Torfeh, M.; McClung, A.; Jung, D. E.; Mansouree, M.; Arbabi, A.; Watkins, J. J. Scalable Nanoimprint Lithography Process for Manufacturing Visible Metasurfaces Composed of High Aspect Ratio TiO2 Meta-Atoms. *ACS Photonics* **2021**, *8* (8), 2400–2409. https://doi.org/10.1021/acsphotonics.1c00609.
(34) Lim, S. W. D.; Meretska, M. L.; Capasso, F. A High Aspect Ratio Inverse-Designed Holey Metalens. *Nano Lett.* **2021**, *21* (20), 8642–8649. https://doi.org/10.1021/acs.nanolett.1c02612.

(35) Ogawa, C.; Nakamura, S.; Aso, T.; Ikezawa, S.; Iwami, K. Rotational Varifocal Moiré Metalens Made of Single-Crystal Silicon Meta-Atoms for Visible Wavelengths. *Nanophotonics* **2022**, *11* (9), 1941–1948. https://doi.org/10.1515/nanoph-2021-0690.
(36) Chen, M. K.; Chu, C. H.; Liu, X.; Zhang, J.; Sun, L.; Yao, J.; Fan, Y.; Liang, Y.; Yamaguchi, T.; Tanaka, T.; Tsai, D. P. Meta-Lens in the Sky. *IEEE Access* **2022**, *10*, 46552–46557. https://doi.org/10.1109/ACCESS.2022.3171351.
(37) Li, Z.; Pestourie, R.; Park, J.-S.; Huang, Y.-W.; Johnson, S. G.; Capasso, F. Inverse Design Enables Large-Scale High-Performance Meta-Optics Reshaping Virtual Reality. *Nat. Commun.* **2022**, *13* (1), 2409. https://doi.org/10.1038/s41467-022-29973-3.
(38) Zhang, L.; Chang, S.; Chen, X.; Ding, Y.; Rahman, M. T.; Yao; Duan; Terekhov, P.; Ni, X. Wafer-Scale Single-Aperture near-Infrared Metalens Fabricated by Deep UV Photolithography. In *Conference on Lasers and Electro-Optics (2022), paper FF2D.4*; Optica Publishing Group, 2022; p FF2D.4. https://doi.org/10.1364/CLEO_QELS.2022.FF2D.4.
(39) Zhang, L.; Chang, S.; Chen, X.; Ding, Y.; Rahman, M. T.; Duan, Y.; Stephen, M.; Ni, X. High-Efficiency, 80 Mm Aperture Metalens Telescope. *Nano Lett.* **2023**, *23* (1), 51–57. https://doi.org/10.1021/acs.nanolett.2c03561.
(40) Hou, M.; Chen, Y.; Yi, F. Lightweight Long-Wave Infrared Camera via a Single 5-Centimeter-Aperture Metalens. In *Conference on Lasers and Electro-Optics (2022), paper FM4F.4*; Optica Publishing Group, 2022; p FM4F.4. https://doi.org/10.1364/CLEO_QELS.2022.FM4F.4.
(41) Li, J.; Wang, Y.; Liu, S.; Xu, T.; Wei, K.; Zhang, Y.; Cui, H. Largest Aperture Metalens of High Numerical Aperture and Polarization Independence for Long-Wavelength Infrared Imaging. *Opt. Express* **2022**, *30* (16), 28882–28891. https://doi.org/10.1364/OE.462251.
(42) Liu, X.; Chen, M. K.; Chu, C. H.; Zhang, J.; Leng, B.; Yamaguchi, T.; Tanaka, T.; Tsai, D. P. Underwater Binocular Meta-Lens. *ACS Photonics* **2023**. https://doi.org/10.1021/acsphotonics.2c01667.
(43) Kim, J.; Seong, J.; Kim, W.; Lee, G.-Y.; Kim, S.; Kim, H.; Moon, S.-W.; Oh, D. K.; Yang, Y.; Park, J.; Jang, J.; Kim, Y.; Jeong, M.; Park, C.; Choi, H.; Jeon, G.; Lee, K.; Yoon, D. H.; Park, N.; Lee, B.; Lee, H.; Rho, J. Scalable Manufacturing of High-Index Atomic Layer–Polymer Hybrid Metasurfaces for Metaphotonics in the Visible. *Nat. Mater.* **2023**, *22* (4), 474–481. https://doi.org/10.1038/s41563-023-01485-5.
(44) Karrfalt, K.; Parks, R. E.; Kim, D. Evaluating SMR Positioning with an Autostigmatic Microscope. In *Optical Manufacturing and Testing XIV (eds Kim, D., Choi, H, Ottevaere, H., Rascher, R.)*; SPIE, 2022; Vol. 12221, pp 216–225. https://doi.org/10.1117/12.2633238.
(45) Lim, S. W. D.; Park, J.-S.; Kazakov, D.; Spägele, C. M.; Dorrah, A. H.; Meretska, M. L.; Capasso, F. Point Singularity Array with Metasurfaces. *Nat. Commun.* **2023**, *14* (1), 3237. https://doi.org/10.1038/s41467-023-39072-6.
